# T_H_17 cells regulate chemokine expression in epithelial cells through C/EBPβ and dictate host sensitivity to colitis and cancer immunity

**DOI:** 10.1126/sciadv.ads3530

**Published:** 2025-08-01

**Authors:** Changsheng Xing, Tianhao Duan, Linfeng Li, Lang Chen, Pengfei Zhang, Yang Du, Siyao Liu, Nihal Annaparthi, Shuo Wang, Chen Qian, Helen Y. Wang, Rong-Fu Wang

**Affiliations:** ^1^ Department of Medicine, Keck School of Medicine, University of Southern California, Los Angeles, CA 90033, USA.; ^2^ Center for Inflammation and Epigenetics, Houston Methodist Research Institute, Houston, TX 77030, USA.; ^3^ Department of Thoracic Surgery, Xiangya Hospital, Central South University, Changsha 410008, China.; ^4^ Department of General Surgery, Third Xiangya Hospital, Xiangya School of Medicine, Central South University, Changsha 410013, China.; ^5^ Department of Molecular Microbiology and Immunology, Keck School of Medicine, University of Southern California, Los Angeles, CA 90033, USA.; ^6^ Department of Pediatrics, Children’s Hospital Los Angeles, Keck School of Medicine, University of Southern California, Los Angeles, CA 90027, USA.; ^7^ Norris Comprehensive Cancer Center, Keck School of Medicine, University of Southern California, Los Angeles, CA 90033, USA.

## Abstract

T_H_17 cells play a critical role in inflammation, cancer development, and antitumor immunity in a context-dependent manner, but detailed mechanisms and their downstream signaling events remain poorly understood. Here, we describe that T_H_17 cytokines strongly inhibit expression of critical chemokines in epithelial tissues, which leads to blocking infiltration of proinflammatory immune cells into the colon, rendering resistance to DSS-induced colitis and colon cancer. We show that key chemokine expression dictates the sensitivity of WT mice to DSS treatment. Mechanistically, we identified C/EBPβ and STAT3 as negative regulators of key chemokine expression following IL-17 and IL-22 stimulation. Knockout of either C/EBPβ or STAT3 in mouse epithelial cells abolished the protective function of T_H_17 cytokines and converted resistant to sensitive phenotype. C/EBPβ ablation in cancer cells markedly enhanced chemokine expression, thus sensitizing cancer cells for anti–PD-1 immunotherapy. Overall, our findings have identified a previously unrecognized critical gap between T_H_17 cytokines, epithelial chemokine expression, and immune cell infiltration through a C/EBPβ-mediated pathway.

## Teaser

T_H_17 cells regulate epithelial chemokine expression through C/EBPβ and dictate host sensitivity to colitis and cancer immunity.

## INTRODUCTION

T helper 17 (T_H_17) cells are constitutively present in the intestine due to microbial flora activation and play an important role in controlling epithelial inflammation and cancer development (*1*, *2*). Colorectal cancer (CRC) is tightly regulated by the local immune system and gut microbiota (*3*), thus becoming an ideal model for investigating the role of T_H_17 cells in cancer immunity. Notably, the functions of T_H_17 cells in cancer immunity are usually context dependent, probably due to different cytokine milieu, tumor models, and disease stages. Regarding the cytokine environment, T_H_17 cells could acquire a pathogenic proinflammatory phenotype with the presence of interleukin-23 (IL-23) or serum amyloid A proteins, while nonpathogenic T_H_17 cells could be induced by IL-6 and transforming growth factor–β (TGF-β) (*4*, *5*). T_H_17 cells generated in different environments may further secrete distinct cytokines and show proinflammatory or immune-suppressive functions (*2*). In different preclinical tumor models, T_H_17 cells are pathogenic in genetically induced sporadic CRC (*6*–*8*) but inhibit the development of colitis-associated CRC (CAC) (*9*–*11*). Similarly, in patients with CRC, T_H_17 cells are increased and associated with tumor progression (*12*–*14*); however, tumor-infiltrated T_H_17 cells positively correlate with improved survival (*15*). In our recent publication (*16*), we showed that microbiota-induced T_H_17 cells protect mice from chemically induced acute colitis and CRC. Therefore, T_H_17 cells exhibit distinct roles in different cancer models, and the underlined mechanisms remain unclear.

Among all T_H_17 cytokines, IL-17A and IL-22 are the most important modulators in CRC development. Although IL-17A contributes to colitis and cancer development (*17*, *18*), it is also critical for intestinal tissue repair, epithelial permeability, and barrier function (*19*–*21*). Similarly, IL-22 enhances barrier function and tissue damage repair (*22*) and protects mice against chemical-induced or T cell–dependent colitis and CRC (*23*–*25*). For example, blockade of IL-17A was ineffective and even exacerbated inflammatory bowel disease in some patients, due to the critical role that IL-17A plays in the maintenance of epithelial barrier homeostasis (*26*). Conversely, in CAC and spontaneous CRC, IL-17A expression is elevated and worsens disease progression (*27*). IL-22 plays a dual role in intestinal health, exhibiting both protective and pathogenic effects depending on the context. In acute injuries, IL-22 contributes to maintaining the epithelial barrier by promoting tissue repair and enhancing epithelial cell survival, thereby limiting infection-induced gut immunopathology (*28*). However, in chronic inflammatory conditions like colitis, IL-22 can exacerbate disease progression. Elevated IL-22 levels have been associated with increased neutrophil recruitment and enhanced inflammation in the colon, suggesting a pathogenic role in chronic colitis (*29*). Therefore, IL-17A and IL-22 have dual roles in maintaining epithelial integrity and promoting inflammation, with their effects varying depending on the disease context. Despite the critical protective roles of T_H_17 cells and their cytokines (IL-17A and IL-22) in tissue integrity and damage repair, the precise regulatory mechanisms of T_H_17 cells and their cytokines in chemokine expression, immune cell trafficking, and the induction of local inflammation remain largely unknown.

Cancer immunotherapy, such as immune checkpoint blockade (ICB) therapy, has shown an impressive and durable clinical response in patients with cancer. However, most patients with cancer do not respond to ICB therapy due to the lack of tumor-infiltrating T cells at tumor sites (so-called “cold tumor”) (*30*, *31*). As key components of the tumor microenvironment, tumor-infiltrating immune cells are critical for patient prognosis and immunotherapeutic efficacy (*31*, *32*). Various chemokines play essential roles in guiding the trafficking of both tumor-activating and tumor-suppressive immune cell types (*33*, *34*). Local chemokines play an important role in mediating the tumor infiltration of antitumor immune cells, thus regulating the efficacy of cancer immunotherapy. The key question is how local chemokine expression is regulated and whether their expression is controlled by T_H_17 cells and the produced cytokines IL-17A and IL-22.

TGF-β–activated kinase-1 (TAK1), encoded by *MAP3K7*, is an essential component in both innate and adaptive immune signaling, but its function is cell-type dependent (*35*). In T cells, TAK1 is an essential positive regulator for cell development and survival (*36*). However, in myeloid cells such as macrophages and neutrophils, TAK1 is a negative regulator of nuclear factor κB (NF-κB) and mitogen-activated protein kinase (MAPK) signaling pathways (*37*). Myeloid-specific Tak1-deficient mice develop splenomegaly and lymphadenopathy, produce large amounts of proinflammatory cytokines and develop severe septic shock after lipopolysaccharide treatment (*37*). Recently, we report that the ablation of TAK1 in myeloid lineage (*Tak1^flox/flox^;Lyz2-Cre^+/+^*, or *Tak1*^Δ*M/*Δ*M*^) renders mice complete resistance to dextran sulfate sodium (DSS)–induced colitis and azoxymethane (AOM)/DSS-induced CRC, mainly through the expansion of protective T_H_17 cells (*16*). *Tak1*^Δ*M/*Δ*M*^ mice have an altered composition of gut microbiota and enhanced production of IL-1β and IL-6, which, in turn, promote T_H_17 cell development in the intestine (*16*). T_H_17 cell depletion converts the resistant to sensitive phenotype in *Tak1*^Δ*M/*Δ*M*^ mice, while adoptive transfer of T_H_17 cells in WT mice renders them resistant to DSS treatment (*16*), indicating that the elevated percentages of T_H_17 cells are required for the resistance of *Tak1*^Δ*M/*Δ*M*^ mice to colitis and for generating protective immunity against cancer.

C/EBPβ (CCAAT/enhancer binding protein β) is a central transcription factor that can be activated by multiple inflammatory stimuli, including IL-17A. In response to IL-17A, the IL-17RA/IL-17RC complex recruits and activates ACT1 (also known as CIKS, encoded by *TRAF3IP2*), which, in turn, phosphorylates tumor necrosis factor receptor–associated factor 6 (TRAF6) and triggers TRAF6-dependent transcription of C/EBPβ and other downstream targets (*38*). C/EBPβ knockout (KO) mice are resistant to T_H_17-dependent experimental autoimmune encephalomyelitis, with reduced expression of T_H_17 cytokines (*39*). Signal transducer and activator of transcription 3 (STAT3) is a critical transcription factor for early development of T_H_17 cells upon the induction by proinflammatory cytokines IL-6 and IL-23 (*40*). STAT3 also serves as a key downstream signaling molecule for IL-22, which is a classic T_H_17 cytokine (*41*). It has been reported that STAT3 directly interacts with C/EBPβ and controls its expression under cytokine stimulation (*42*). However, the precise mechanism of how C/EBPβ, STAT3, and their signaling pathways negatively regulate key chemokine expression, immune cell trafficking, and antitumor immunity remains largely unknown.

In this study, we investigated the mechanisms by which T_H_17 cells and their cytokines (IL-17A and IL-22) control local inflammation and render resistance to DSS-induced colitis and CRC development. We found that T_H_17 cells and their IL-17A and IL-22 render resistance to DSS-induced colitis and CRC development through blocking immune cell infiltration into the local epithelial tissues, which is associated with chemokine expression in epithelial tissues. We provided key evidence that T_H_17 cells and their IL-17A and IL-22 inhibited expression of several key chemokines (CCL5, CXCL5, and CXCL10). To substantiate these findings, we further showed that mice deficient in CCL5 or CXCL10 could ameliorate the in vivo pathogenesis of colitis and CRC. Mechanistically, we demonstrated that IL-17A and IL-22 activated C/EBPβ and STAT3, respectively, which, in turn, functioned as key negative regulators to inhibit chemokine expression in epithelial tissues. KO of either C/EBPβ or STAT3 in colon epithelial cells abolished the protective functions of IL-17A and IL-22 against DSS-induced colitis. Furthermore, C/EBPβ KO could promote chemokine expression in multiple types of cancer cells, thus potentially enhancing the sensitivity of programmed cell death protein 1 (PD-1) antibody (Ab)–mediated cancer immunotherapy. Overall, our findings have provided important molecular insights into the mechanisms by which T_H_17 cells and their cytokines render resistance to DSS-induced colitis through blocking immune cell infiltration and chemokine expression. We have also identified C/EBPβ as a critical regulatory molecule and therapeutic target for cancer immunotherapy, thus providing significant scientific and concept advances.

## RESULTS

### Infiltration of immune cells into colon tissues is closely linked to the sensitivity of mice to DSS treatment

**Fig. 1.**
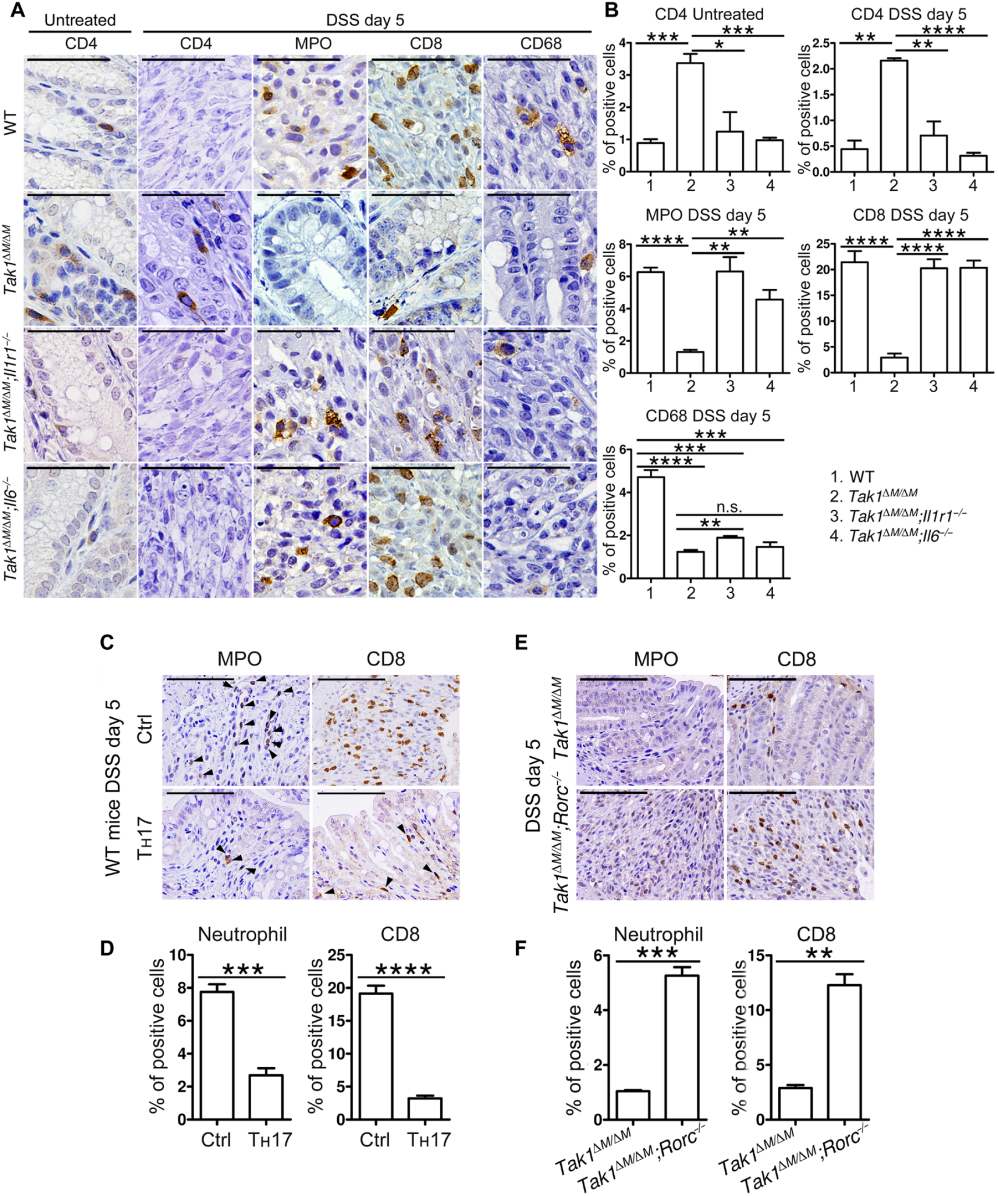
Infiltration of immune cells into colon tissues is closely linked to the sensitivity of mice to DSS treatment. (**A** and **B**) IHC staining of CD4, CD8, CD68 (macrophage/monocyte), and MPO (neutrophil) (40×) on colon sections from WT, *Tak1*^Δ*M/*Δ*M*^, and DKO mice with or without DSS treatment (scale bars, 100 μm), and the analyses of positive cell percentage. Representative data from three independent experiments, means ± SEM. (**C** and **D**) Intestinal LPLs were purified from *Tak1*^Δ*M/*Δ*M*^*;Il17-GFP^+^* mice. Control CD4^+^ (CD4^+^/GFP^−^) and T_H_17 cells (CD4^+^/GFP^+^) were sorted and injected intraperitoneally to WT mice. Mice were then treated with 5% DSS for 5 days. (C) IHC staining of MPO and CD8 (40×) on colon sections collected on day 5 (scale bars, 100 μm). (D) Analyses of positive cell percentage. Combined data from two independent experiments, means ± SEM. (**E** and **F**) *Tak1*^Δ*M/*Δ*M*^ and *Tak1*^Δ*M/*Δ*M*^*;Rorc^−/−^* mice were treated with 5% DSS for 5 days. (E) IHC staining of MPO and CD8 (40×) on colon sections collected on day 5 (scale bars, 100 μm). (F) Analyses of positive cell percentage. Representative data from two independent experiments, means ± SEM. Statistical analyses: Student’s unpaired *t* test (B, D, and F). *P < 0.05; **P < 0.01; ***P < 0.001; ****P < 0.0001. n.s., not significant; Ctrl, control.

We previously demonstrated that the accumulated CD4^+^ T_H_17 cells in colon tissues are critical to protect *Tak1*^Δ*M/*Δ*M*^ mice against colitis and CRC (*16*). However, how CD4^+^ T_H_17 cells maintain the tissue integrity and mediate protection against DSS-induced colitis and tissue damage remains largely unknown. It has been known that colon-infiltrating immune cells, including neutrophils, macrophages, and CD8^+^ cells, could regulate inflammation and tissue damage in response to DSS treatment (*43*, *44*). To address this issue, we examined immune cell populations in colitis-sensitive and colitis-resistant mouse models by immunohistochemistry (IHC) staining of myeloperoxidase (MPO; for neutrophils), CD68 (for macrophages/monocytes), CD4, and CD8 on colon sections from different mouse strains. We found that the number of tissue-resident CD4^+^ cells in the colons of *Tak1*^Δ*M/*Δ*M*^ mice (colitis resistant) was much higher than in wild-type (WT) and double KO (DKO) mice (*Tak1*^Δ*M/*Δ*M*^*;ll1r1^−/−^* and *Tak1*^Δ*M/*Δ*M*^*;ll6*^−/−^) (colitis sensitive) before DSS treatment and remained at a significantly higher level even after DSS treatment (Fig. 1, A and B). By contrast, the numbers of colon-infiltrating neutrophils, macrophages, and CD8^+^ cells were markedly increased in WT mice, compared with those in *Tak1*^Δ*M/*Δ*M*^ mice after DSS treatment. Neutrophils and CD8^+^ cells, but not macrophages, were markedly increased in DSS-treated *Tak1*^Δ*M/*Δ*M*^*;ll1r1^−/−^* and *Tak1*^Δ*M/*Δ*M*^*;ll6^−/−^* mice, compared with those in *Tak1*^Δ*M/*Δ*M*^ mice after DSS treatment (Fig. 1, A and B), suggesting that the colon infiltration of neutrophils and CD8^+^ cells may be closely linked to the sensitivity of mice to DSS treatment. The lower-magnification IHC images were shown for better visualization (fig. S1A).

To further substantiate these findings, we performed flow cytometry analysis in the layer of the colon lamina propria (LP). We showed that the percentages of neutrophils and macrophages were relatively low in both WT and *Tak1*^Δ*M/*Δ*M*^ mice before DSS treatment (fig. S1, B and C). After the treatment, neutrophils and macrophages were increased in both WT and *Tak1*^Δ*M/*Δ*M*^ mice; however, their levels in *Tak1*^Δ*M/*Δ*M*^ mice were significantly lower than those in WT mice (fig. S1, B and C). We further found that the CD4^+^ cells in colon LP remained at a low level in WT mice with or without DSS treatment (fig. S1, D and E). By contrast, significantly elevated percentages of CD4^+^ cells in *Tak1*^Δ*M/*Δ*M*^ mice were maintained before and after DSS treatment (fig. S1, D and E), consistent to the IHC staining results. In addition, the percentage of CD8^+^ population was slightly increased in WT mice after DSS treatment (fig. S1, D and E). However, the CD8^+^ cells in the colon LP of *Tak1*^Δ*M/*Δ*M*^ mice were constitutively higher than those of WT mice before DSS treatment and remained unchanged after DSS treatment (fig. S1, D and E). It should be noted that the CD8^+^ cell levels showed discrepancies between LP lymphocytes (LPLs) and intraepithelial lymphocytes (IELs). IELs (examined by IHC staining) are in the epithelial layer and located between the epithelial cells lining the gut, while LPLs (examined by flow cytometry) are in LP, the connective tissue layer beneath the epithelium. At IEL level (IHC staining), CD8^+^ cell infiltration was significantly decreased in *Tak1*^Δ*M/*Δ*M*^ mice after DSS treatment. However, a higher percentage of CD8^+^ cells was found at LPL level (flow cytometry) in *Tak1*^Δ*M/*Δ*M*^ mice, with or without DSS treatment, suggesting that the high levels of CD4^+^ T cells (particularly CD4^+^ T_H_17 cells) within the epithelium may inhibit CD8^+^ T cell infiltration from LP into the epithelial layer, even before DSS treatment. Because this study mainly focused on the chemokines produced by the epithelial cells, the IEL levels are more relevant to determining the immune cell infiltration.

We further investigated the potential correlation of proinflammatory immune cells with the sensitivity of host to DSS treatment in additional mouse models. In our previous study, we found that the adoptive transfer of protective T_H_17 cells from *Tak1*^Δ*M/*Δ*M*^ mice into WT recipient renders partial resistance to colitis, while the depletion of T_H_17 cell by retinoic acid receptor–related orphan receptor-γt (RORγt) KO in colitis-resistant *Tak1*^Δ*M/*Δ*M*^ mice turns the mice completely sensitive (*16*). Here, we showed that the colon-infiltrated neutrophils and CD8^+^ cells were significantly reduced in T_H_17 cell–transferred WT mice, compared with those in WT controls (Fig. 1, C and D). Furthermore, the immune populations were significantly increased in *Tak1*^Δ*M/*Δ*M*^*;Rorc^−/−^* mice, which were completely sensitive in the colitis model (*16*), compared with those in *Tak1*^Δ*M/*Δ*M*^ mice (Fig. 1, E and F). The infiltration patterns of these immune cells were closely correlated with the sensitivity of different mouse strains to DSS-induced colitis. Together, our results suggest that the infiltration of proinflammatory immune cells into the colon tissues likely increases the sensitivity to DSS-induced inflammation and tissue destruction.

### Chemokine expression is inhibited in the colon tissues of colitis-resistant mice

**Fig. 2.**
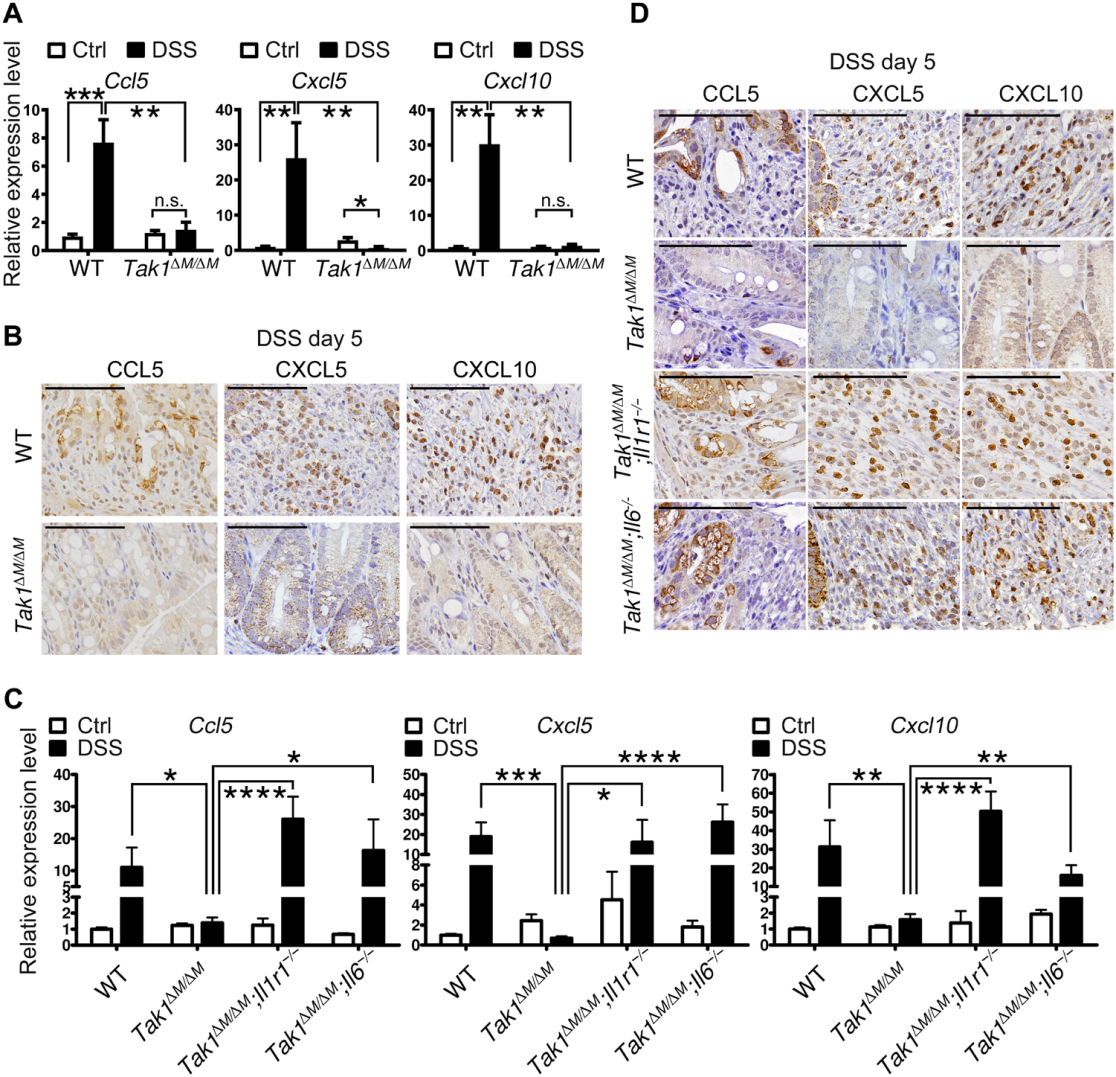
Chemokine expression is inhibited in the colon tissues of colitis-resistant mice. (**A**) WT and *Tak1*^Δ*M/*Δ*M*^ mice were treated with water control or 2.5% DSS for 5 days. On day 8, chemokine expression in colon was measured by qPCR. Representative data from three independent experiments, means ± SEM. (**B**) IHC staining of CCL5, CXCL5, and CXCL10 (40×) on colon sections collected on day 5 after 5% DSS treatment (scale bars, 100 μm). Representative data from three independent experiments. (**C**) WT, *Tak1*^Δ*M/*Δ*M*^, *Tak1*^Δ*M/*Δ*M*^*;Il1r1*^−/−^ and *Tak1*^Δ*M/*Δ*M*^*;Il6*^−/−^ mice were treated with water control or 2.5% DSS for 5 days. On day 8, chemokine expression in colon was measured by qPCR. Representative data from three independent experiments, means ± SEM. (**D**) IHC staining of CCL5, CXCL5, and CXCL10 (40×) on colon sections from WT, *Tak1*^Δ*M/*Δ*M*^, *Tak1*^Δ*M/*Δ*M*^*;Il1r1*^−/−^, and *Tak1*^Δ*M/*Δ*M*^*;Il6*^−/−^ mice collected on day 5 after 5% DSS treatment (scale bars, 100 μm). Representative data from three independent experiments. Statistical analyses: Student’s unpaired *t* test (A and C). *P < 0.05; **P < 0.01; ***P < 0.001; ****P < 0.0001. n.s., not significant; Ctrl, control.

Next, we sought to determine the potential key factors that control immune cell infiltration into the colon tissues after DSS treatment. Chemokines and chemokine receptors play important roles in regulating immune cell trafficking and gut inflammation (*45*). To determine whether chemokines are involved in the immune infiltration, we performed experiments to assess chemokine expression in (DSS-sensitive) WT and (DSS-resistant) *Tak1*^Δ*M/*Δ*M*^ mice before and after DSS treatment by quantitative polymerase chain reaction (qPCR) analysis. Our chemokine screening revealed a unique expression pattern of colon chemokines in *Tak1*^Δ*M/*Δ*M*^ mice. DSS treatment could induce significantly increased expression of 13 chemokine genes in WT mice (Fig. 2A and fig. S2A). By contrast, their expression levels in DSS-treated *Tak1*^Δ*M/*Δ*M*^ mice were significantly reduced, compared to those in DSS-treated WT mice (Fig. 2A and fig. S2A). Other chemokine genes did not meet these criteria (fig. S2B) and were not selected for further analysis. While most chemokines remained the same expression levels in *Tak1*^Δ*M/*Δ*M*^ mice before and after DSS treatment, *Cxcl5*, *Ccl2*, and *Cxcl11* showed statistically significant changes. However, their overall expression levels remained very low, particularly compared with those in DSS-treated WT mice (Fig. 2A and fig. S2A). Because mRNA expression level does not always correlate with the protein level, we further examined the chemokine expression by IHC staining. We found that the protein levels of CCL5, CXCL5, and CXCL10 were notably increased in the colon tissue of WT mice after DSS treatment but were lower in *Tak1*^Δ*M/*Δ*M*^ mice than those in WT controls (Fig. 2B and fig. S3A). There were no appreciable differences at the protein expression levels of other chemokines in colon tissues between DSS-treated WT and *Tak1*^Δ*M/*Δ*M*^ mice (fig. S3B). On the basis of these findings, we focused on CCL5, CXCL5, and CXCL10 in our subsequent studies.

Ablation of the *Il1r1* or *Il6* gene in *Tak1*^Δ*M/*Δ*M*^ mice restored the expression of these three chemokines in the colon to levels that were similar to or higher than those in WT mice after DSS treatment (Fig. 2, C and D), suggesting that expression of CCL5, CXCL5, and CXCL10 reversely correlates with the sensitivity of different mouse strains to DSS treatment. Previous studies have reported that CXCL5 mediates neutrophil chemotaxis (*46*), whereas CCL5 and CXCL10 could attract CD8^+^ T cells (*47*, *48*). Thus, our results suggest that the inhibition of CCL5, CXCL5, and CXCL10 chemokine expression in the colon may be responsible for the observed blocking of neutrophil and CD8^+^ T cell trafficking into DSS-treated colon tissues in *Tak1*^Δ*M/*Δ*M*^ mice.

### T_H_17 cells and their cytokines inhibit key chemokine expression in the colon after DSS treatment

**Fig. 3.**
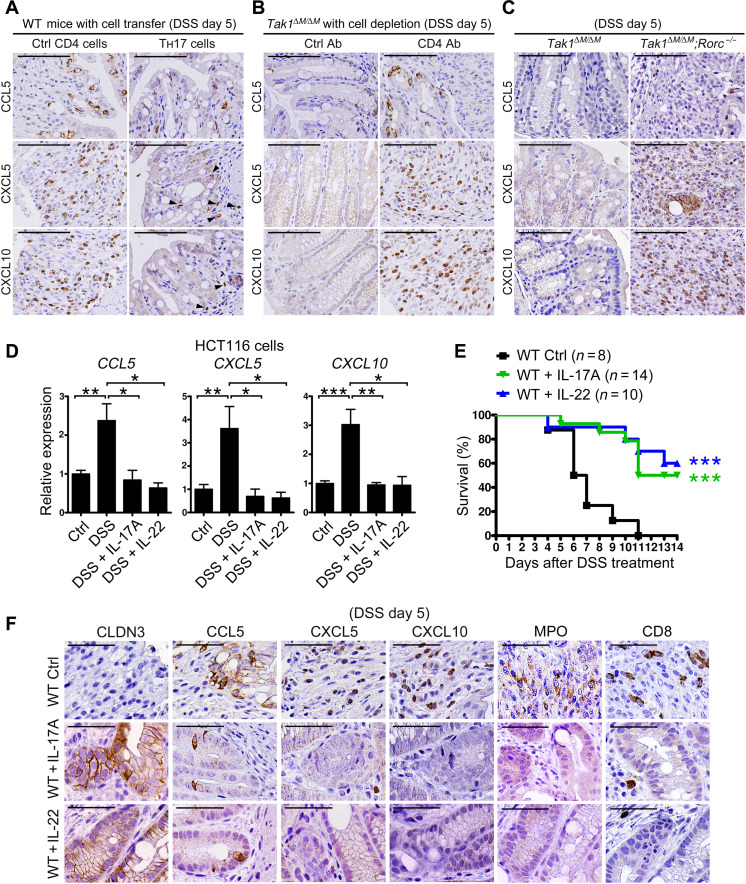
T_H_17 cells and their cytokines inhibit key chemokine expression in the colon after DSS treatment. (**A** to **C**) IHC staining of CCL5, CXCL5, and CXCL10 (40×) on colon sections from control CD4^+^ or T_H_17 cell–injected WT mice (A), control immunoglobulin G or CD4-depleting Ab–injected *Tak1*^Δ*M/*Δ*M*^ mice (B), or *Tak1*^Δ*M/*Δ*M*^*;Rorc^−/−^* mice (C). Tissues were collected on day 5 after 5% DSS treatment (scale bars, 100 μm). Representative data from two or three independent experiments. (**D**) HCT116 cells were pretreated with BSA control, recombinant IL-17A, or IL-22, followed by overnight 2% DSS treatment. Control buffer and cytokines were added when replacing the medium. Chemokine expression was tested by qPCR on day 4. Representative data from two independent experiments, means ± SEM. (**E**) WT mice were intraperitoneally injected with BSA buffer as control (*n* = 8) or recombinant IL-17A (*n* = 14) and IL-22 (*n* = 10) and treated with 5% DSS for 5 days. Survival curves were observed. Combined data from two independent experiments. (**F**) IHC staining of CCL5, CXCL5, CXCL10, CLDN3, MPO, and CD8 (40×) on colon sections collected on day 5 after DSS treatment (scale bars, 50 μm). Representative data from two independent experiments. Statistical analyses: Student’s unpaired *t* test (D) and Mantel-Cox log-rank test (E). *P < 0.05; **P < 0.01; ***P < 0.001. Ctrl, control.

The next key question is how expression of CCL5, CXCL5, and CXCL10 chemokines is inhibited in *Tak1*^Δ*M/*Δ*M*^ mice, compared with that in WT mice. To our knowledge, it has not been reported that T_H_17 cells and their cytokines (such as IL-17A and IL-22) could inhibit CCL5, CXCL5, and CXCL10 expression in the colon tissues. We hypothesized that T_H_17 cells and their cytokines could inhibit CCL5, CXCL5, and CXCL10 chemokine expression in colon tissues. To test this possibility, we sorted the T_H_17 cells from *Tak1*^Δ*M/*Δ*M*^*;Il17-GFP^+^* mice [expressing transgenic green fluorescent protein (GFP) in IL-17A–producing cells], transferred the sorted T_H_17 cells into WT mice, and then treated the mice with DSS for 5 days. IHC staining revealed that CCL5, CXCL5, and CXCL10 expression in colon tissues from T_H_17 cell–transferred WT mice was substantially reduced, compared with that in colon tissues from control T cell–transferred WT mice (Fig. 3A), suggesting the inhibition of DSS-induced chemokine expression in the colon tissues by T_H_17 cells. By contrast, Ab depletion of CD4^+^ cells in *Tak1*^Δ*M/*Δ*M*^ mice and genetic depletion of IL-17–producing cells in *Tak1*^Δ*M/*Δ*M*^*;Rorc^−/−^* mice led to increased expression of colon chemokines (CCL5, CXCL5, and CXCL10) upon DSS treatment (Fig. 3, B and C), suggesting that T_H_17 cells from *Tak1*^Δ*M/*Δ*M*^ mice are directly responsible for suppressing chemokine expression in colon tissues after DSS treatment. The chemokine levels in these IHC images were further measured by the “H-score,” which captures both the staining intensity and the proportion of biomarkers. We found that all the differences were statistically significant (fig. S4, A to C).

To further determine which T_H_17 cytokines are responsible for inhibiting chemokine expression and protect the *Tak1*^Δ*M/*Δ*M*^ mice against colitis and CRC, we performed intracellular staining of key T_H_17 cytokines (IL-17A, IL-17F, IL-21, and IL-22) in CD4^+^ cells from intestinal LP. The flow cytometry analysis revealed that the gated T_H_17 cells (CD4^+^/IL-17A^+^) produced IL-22 in 40% of this subset but did not produce IL-17F or IL-21 (fig. S4D). IL-22 is another protective T_H_17-produced cytokine that protects against chemically induced colitis and CRC (*23*, *24*). Consistently, besides the increased IL-17A level (*16*), the serum level of IL-22 was significantly increased in *Tak1*^Δ*M/*Δ*M*^ mice, whereas IL-17F and IL-21 levels remained low, similar to the levels in WT mice (fig. S4E).

To further define the role of key T_H_17 cytokines in inhibiting chemokine expression, we treated human epithelial colon cancer cells (HCT116) with recombinant IL-17A and IL-22 and then determined the chemokine expression after DSS treatment. We found that the expression of *CCL5*, *CXCL5*, and *CXCL10* was markedly increased in HCT116 cells after DSS treatment, but the addition of either exogenous IL-17A or IL-22 strongly inhibited the expression of CCL5, CXCL5, and CXCL10 (Fig. 3D). By contrast, other cytokines such as IL-1β and IL-6 produced in *Tak1*^Δ*M/*Δ*M*^ mice had no effect on the chemokine expression in HCT116 cells after DSS treatment (fig. S4F). These data suggest that IL-17A and IL-22 directly inhibit chemokine expression in intestinal epithelial cells, thus preventing the infiltration of immune cells into colon tissues after DSS treatment.

To substantiate the in vivo function of IL-17A and IL-22 in the inhibition of chemokine expression in colon epithelia, we treated WT mice with exogenous IL-17A, IL-22, or bovine serum albumin (BSA) control and then followed by 5% DSS treatment. We found that IL-17A– or IL-22–treated mice had significantly extended survival, compared to BSA-treated control mice (Fig. 3E), with an ameliorated colon structure and increased expression of claudin 3 (Fig. 3F), which is a key tight junction protein in the intestine for barrier function. Consistently, the chemokine expression in colon tissues from T_H_17 cytokine–treated mice was much lower than in BSA-treated control mice at day 5 after DSS treatment (Fig. 3F). The colon infiltration of neutrophils and CD8^+^ cells was significantly decreased in IL-17A– and IL-22–treated mice, compared with that in the BSA-treated control mice (Fig. 3F and fig. S4G). These results suggest that T_H_17 cells and their cytokines (IL-17A and IL-22) markedly inhibit CCL5, CXCL5, and CXCL10 expression in the colon tissues after DSS treatment.

### Key chemokines are required for immune cell infiltration to promote colitis and colon cancer

**Fig. 4.**
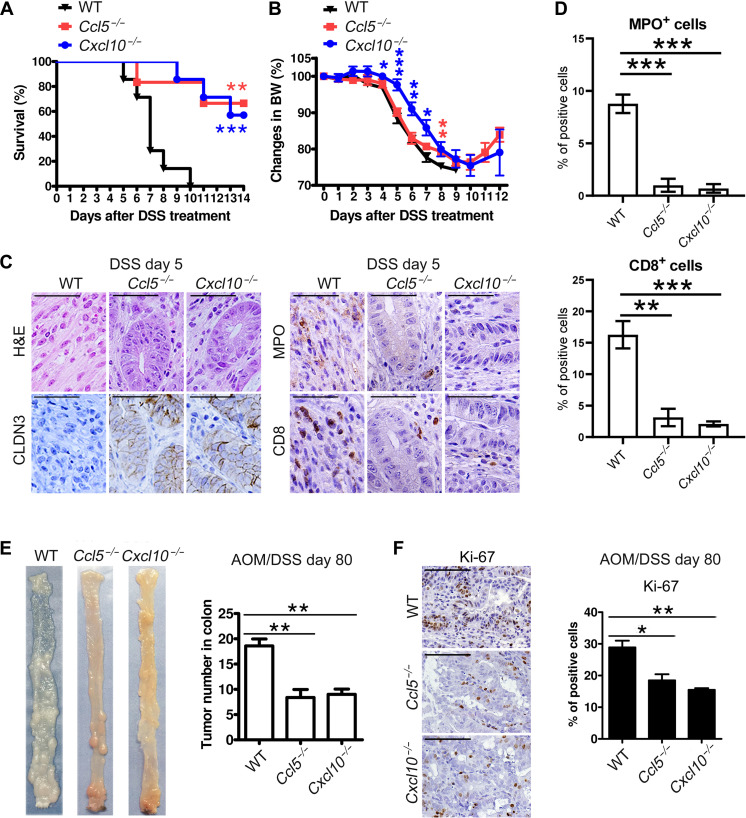
Key chemokines are required for immune cell infiltration to promote colitis and colon cancer. (**A** and **B**) Survival curves and body weights (BW) of WT (*n* = 7), *Ccl5^−/−^* (*n* = 6), and *Cxcl10^−/−^* (*n* = 7) mice with 5% DSS treatment for 5 days. Combined data from two independent experiments. (**C** and **D**) Hematoxylin and eosin (H&E) staining and IHC staining of CLDN3, MPO, and CD8 on colon sections collected on day 5 (scale bars, 50 μm), and the analyses of positive immune cell percentage. Representative data from two independent experiments, means ± SEM. (**E**) Colon tumors in WT (*n* = 5), *Ccl5^−/−^* (*n* = 5), and *Cxcl10^−/−^* (*n* = 4) mice on day 80 after AOM/DSS treatment (left), and the analysis of tumor numbers (right). Representative data from two independent experiments, means ± SEM. (**F**) IHC staining of Ki-67 (40×) on colon sections collected on day 80 after AOM/DSS treatment (left; scale bars, 100 μm), and the analysis of positive cell percentage (right). Representative data from two independent experiments, means ± SEM. Statistical analyses: Mantel-Cox log-rank test (A), ANOVA and student’s unpaired *t* test (B), Student’s unpaired *t* test (D; E, right; and F, right). *P < 0.05; **P < 0.01; ***P < 0.001.

To determine whether CCL5 and CXCL10 are required for DSS-induced colitis, we obtained WT, *Ccl5*- and *Cxcl10*-deficient mice from the Jackson Laboratory and treated them with 5% DSS. We found that WT mice died within 10 days, but *Ccl5^−/−^* and *Cxcl10^−/−^* mice had significantly ameliorated body weights and survival, with more than 50% surviving at 14 days (Fig. 4, A and B). While the untreated *Ccl5^−/−^* and *Cxcl10^−/−^* mice did not have any tissue abnormality in colon (fig. S4H), the epithelial structures and CLDN3 staining were only slightly damaged in DSS-treated *Ccl5^−/−^* and *Cxcl10^−/−^* mice, compared with those in WT mice with complete tissue damage and loss of CLDN3 staining (Fig. 4C). The colon infiltrations of neutrophils and CD8^+^ cells were significantly decreased in chemokine-deficient mice (Fig. 4, C and D).

Next, we used AOM/DSS-induced colon cancer model to determine whether CCL5 and CXCL10 are required for CRC development. We found that *Ccl5^−/−^* and *Cxcl10^−/−^* mice developed fewer tumors than did WT mice (Fig. 4E). IHC staining of Ki-67 on colon sections further revealed a marked decrease in colon epithelial cell proliferation in *Ccl5^−/−^* and *Cxcl10^−/−^* mice, compared with Ki-67 staining in the colon epithelial cells of WT mice (Fig. 4F). These results suggest that the ablation of CCL5 and CXCL10 expression can block immune cell infiltration into colon tissues after DSS treatment, thus resisting to DSS-induced colitis and CRC development.

### T_H_17 cytokines regulate chemokine expression through C/EBPβ and STAT3 signaling

**Fig. 5.**
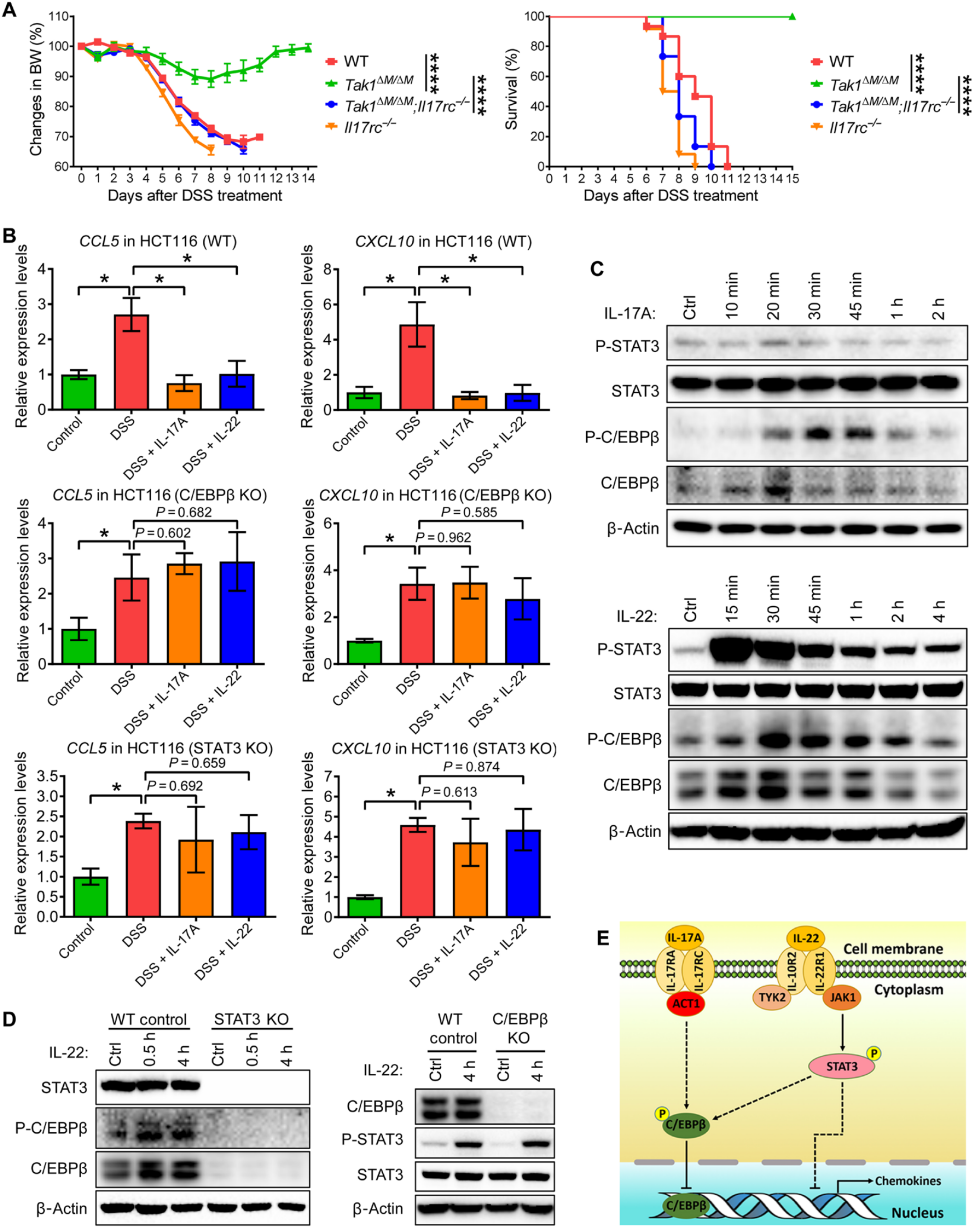
T_H_17 cytokines regulate chemokine expression through C/EBPβ and STAT3 signaling. (**A**) Survival curves and body weight (BW) changes of WT (*n* = 15), *Il17rc^−/−^* (*n* = 12), *Tak1*^Δ*M/*Δ*M*^ (*n* = 8), and *Tak1*^Δ*M/*Δ*M*^*;Il17rc^−/−^* (*n* = 15) mice with 5% DSS treatment for 5 days. Combined data from three independent experiments, means ± SEM. BW, (**B**) HCT116 cells (WT, C/EBPβ KO, and STAT3 KO) were pretreated with BSA control, recombinant IL-17A, or IL-22, followed by overnight 2% DSS treatment. Control buffer and cytokines were added when replacing the medium. Chemokine expression was tested by qPCR on day 4. Representative data from two independent experiments, means ± SEM. (**C**) WT HCT116 cells were treated with recombinant IL-17A or IL-22, and cell lysates were harvested at different time points. Western blotting was performed to determine the expression and activation of C/EBPβ and STAT3. Representative data from three independent experiments. (**D**) HCT116 cells (WT, C/EBPβ KO, and STAT3 KO) were treated with recombinant IL-22, and cell lysates were harvested at different time points. Western blotting was performed to determine the expression and activation of C/EBPβ and STAT3 in the KO cells. Representative data from three independent experiments. (**E**) A diagram showing the putative downstream signaling and key factors for IL-17A and IL-22 to regulate the chemokine expression in colon epithelial cells. Statistical analyses: ANOVA and Student’s unpaired *t* test (A, left), Mantel-Cox log-rank test (A, right), and Student’s unpaired *t* test (B). *P < 0.05; ****P < 0.0001. Ctrl, control; h, hour.

To dissect the molecular mechanisms by which T_H_17 cytokines regulate chemokine expression in colon epithelial cells, we first tested whether key receptor IL-17RC was required for IL-17A–mediated inhibition of CCL5, CXCL5, and CXCL10 expression. To this end, we generated *Tak1*^Δ*M/*Δ*M*^*;Il17rc^−/−^* mice, in which the key receptor IL-17RC was ablated to block IL-17A signaling. After DSS treatment, IL-17RC KO in *Tak1*^Δ*M/*Δ*M*^ mice completely converted the resistant phenotype to the sensitive phenotype (Fig. 5A), suggesting that IL-17RC receptor is required for IL-17A–mediated inhibition of chemokine expression and resistance to DSS-induced colitis. Because ACT1 is the direct adaptor of the IL-17R complex, we next knocked down *ACT1* in HCT116 cells and found that *ACT1* knockdown (KD) abolished the ability of IL-17A to inhibit chemokine expression (fig. S5, A and B), suggesting that IL-17R–ACT1 signaling is required for IL-17–mediated inhibition of CCL5, CXCL5, and CXCL10 expression.

The downstream signaling pathway of IL-17R–ACT1 has not been well-defined. Previous studies suggest that ACT1–phosphatidylinositol 3-kinase (PI3K)–Akt axis, NF-κB signaling, extracellular signal–regulated kinase (ERK)–MAPK signaling, and C/EBPβ are involved in the IL-17A downstream signaling in epithelial cells (*38*, *49*, *50*), while IL-22 and its receptors appear to regulate MAPK and Akt signaling through STAT1, STAT3, and STAT5 molecules (*41*, *51*). To identify the key signaling molecules that could mediate the inhibition of chemokine expression by IL-17A and IL-22, we generated a panel of HCT116 cell lines with deficient in each candidate gene using a CRISPR-Cas9 technology. The KO efficacy of each molecule was validated by Western blotting (fig. S5C). We next treated WT HCT116 and KO cells with IL-17A or IL-22 and then determined chemokine expression after DSS treatment. We found that the expression of *CCL5* and *CXCL10* could not be inhibited by exogenous IL-17A or IL-22 in C/EBPβ KO cells, compared with that in WT cells (Fig. 5B). Similarly, we showed that IL-17A and IL-22 failed to inhibit *CCL5* and *CXCL10* expression in STAT3 KO cells (Fig. 5B). By contrast, the KO of other candidate signaling molecules did not have any effect on *CCL5* and *CXCL10* expression after treatment with IL-17A or IL-22 (fig. S5D). These data suggest that C/EBPβ and STAT3 are responsible for the inhibition of *CCL5* and *CXCL10* expression by IL-17A or IL-22 after DSS treatment.

Next, we sought to determine how C/EBPβ and STAT3 respond to IL-17A or IL-22 treatment in colon epithelial cells. We treated the HCT116 cells with IL-17A or IL-22, harvested the protein samples at different time points, and performed Western blotting to determine the activation (phosphorylation) of C/EBPβ and STAT3. We found that the phosphorylated (P-) C/EBPβ level was markedly increased at 20 min and peaked at 30 min after IL-17A stimulation, while the STAT3 levels (P-STAT3 and total STAT3) were not changed (Fig. 5C). The phosphorylated C/EBPβ was reduced at 1 to 2 hours after IL-17A treatment. Upon IL-22 treatment, the phosphorylated STAT3 was rapidly induced and peaked at 15 min and then gradually reduced but maintained activation for several hours, while the total STAT3 protein maintained at the similar level (Fig. 5C). We also found that phosphorylated C/EBPβ was induced at 30 min (which was slightly later than STAT3 phosphorylation) and maintained at 1 hour after IL-22 treatment. Total C/EBPβ levels were increased at 15 and 30 min after IL-22 treatment (Fig. 5C). Notably, STAT3 has been reported to directly interact with C/EBPβ, stabilize C/EBPβ protein in epithelial cells, and control C/EBPβ expression under cytokine stimulation (*42*, *52*, *53*). We next investigate the potential links between the enhanced C/EBPβ level and STAT3 signaling after IL-22 treatment and notably found that C/EBPβ protein was not detectable in STAT3 KO HCT116 cells (Fig. 5D), strongly suggesting that STAT3 is critically required for the stability of C/EBPβ. By contrast, C/EBPβ KO in HCT116 cells did not affect the P-STAT3 and total STAT3 levels (Fig. 5D). Together, these data suggest that C/EBPβ is a critical negative regulator of chemokine expression in epithelial cells through IL-17A stimulation, while STAT3 can be activated by IL-22, which, in turn, regulates C/EBPβ and subsequent chemokine expression (Fig. 5E).

### T_H_17 cytokines fail to protect C/EBPβ or STAT3 KO mice from DSS-induced mortality

**Fig. 6.**
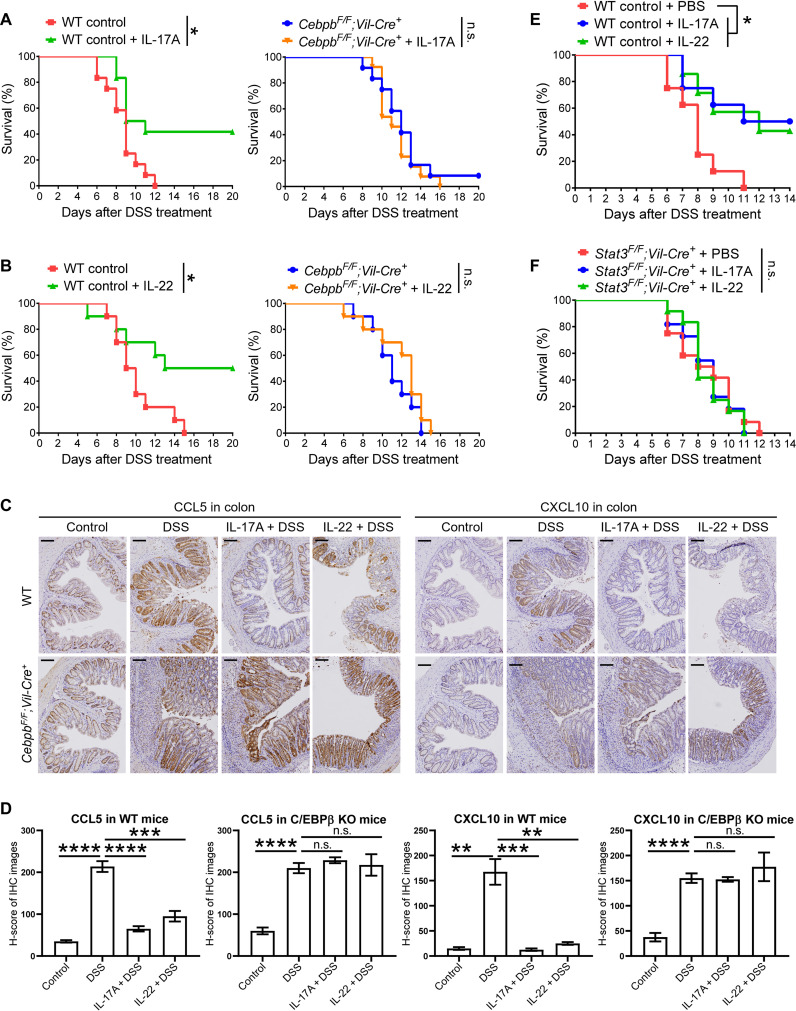
T_H_17 cytokines fail to protect C/EBPβ or STAT3 KO mice against colitis. (**A**) WT and *Cebpb^F/F^;Vil-Cre^+/+^* mice were intraperitoneally injected with BSA buffer as control (WT, *n* = 12; KO, *n* = 12) or recombinant IL-17A (WT, *n* = 12; KO, *n* = 13) and treated with 5% DSS for 5 days. Survival curves were observed. Combined data from three independent experiments. (**B**) WT and *Cebpb^F/F^;Vil-Cre^+/+^* mice were intraperitoneally injected with BSA buffer as control or recombinant IL-22 and treated with 5% DSS for 5 days (*n* = 10 for all groups). Survival curves were observed. Combined data from two independent experiments. (**C** and **D**) IHC staining of CCL5 and CXCL10 (10×) on colon sections collected on day 7 after 2.5% DSS treatment for 5 days (scale bars, 100 μm), with the quantification and statistical analyses of chemokine levels by the H-score of the IHC images. Representative data from two independent experiments, means ± SEM. (**E** and **F**) WT and *Stat3^F/F^;Vil-Cre^+/+^* mice were intraperitoneally injected with BSA buffer as control (WT, *n* = 8; KO, *n* = 12) or recombinant IL-17A (WT, *n* = 8; KO, *n* = 11) or IL-22 (WT, *n* = 7; KO, *n* = 12) and treated with 5% DSS for 5 days. Survival curves were observed. Combined data from two independent experiments. Statistical analyses: Mantel-Cox log-rank test (A, B, E, and F) and Student’s unpaired *t* test (D). *P < 0.05; **P < 0.01; ***P < 0.001; ****P < 0.0001. n.s., not significant.

To substantiate these findings in physiological conditions, we generated *Cebpb^F/F^;Vil-Cre^+^* mice, in which C/EBPβ was specifically deleted in the intestinal epithelial cells by Villin-Cre. We injected the mice with T_H_17 cytokines or BSA control and tested their sensitivity to DSS-induced colitis. As expected, WT control mice were partially resistant to DSS treatment after the administration of IL-17A or IL-22 (Fig. 6, A and B), with markedly ameliorated body weight loss, compared to the control mice (fig. S6, A and B). By contrast, the ablation of C/EBPβ in colon epithelial cells completely abolished the protective effects of T_H_17 cytokines (Fig. 6, A and B), with similar levels of body weight loss with or without cytokine administration (fig. S6, A and B). Furthermore, colon chemokine expression (CCL5 and CXCL10) could be significantly inhibited by T_H_17 cytokines in WT mice, but not in *Cebpb^F/F^;Vil-Cre^+^* mice (Fig. 6, C and D). Consistently, the infiltration of proinflammatory immune cells (neutrophils and CD8^+^ cells) was significantly inhibited by T_H_17 cytokines in WT mice, but not in *Cebpb^F/F^;Vil-Cre^+^* mice (fig. S6, C and D). These data provide the direct in vivo evidence that C/EBPβ is a central negative regulator of chemokine expression mediated by IL-17A and IL-22. The KO efficacy was validated by IHC staining of C/EBPβ in untreated WT and KO mice (fig. S6E). With a similar strategy, we generated *Stat3^F/F^;Vil-Cre^+^* mice and found that administration of IL-17A or IL-22 failed to protect the mice from DSS-induced colitis (Fig. 6, E and F, and fig. S6F). These data further demonstrate the critical roles of C/EBPβ and STAT3 in controlling chemokine expression and acute inflammation through IL-17A and/or IL-22.

### C/EBPβ KO enhances chemokine expression in cancer cells and promotes the efficacy of cancer immunotherapy

**Fig. 7.**
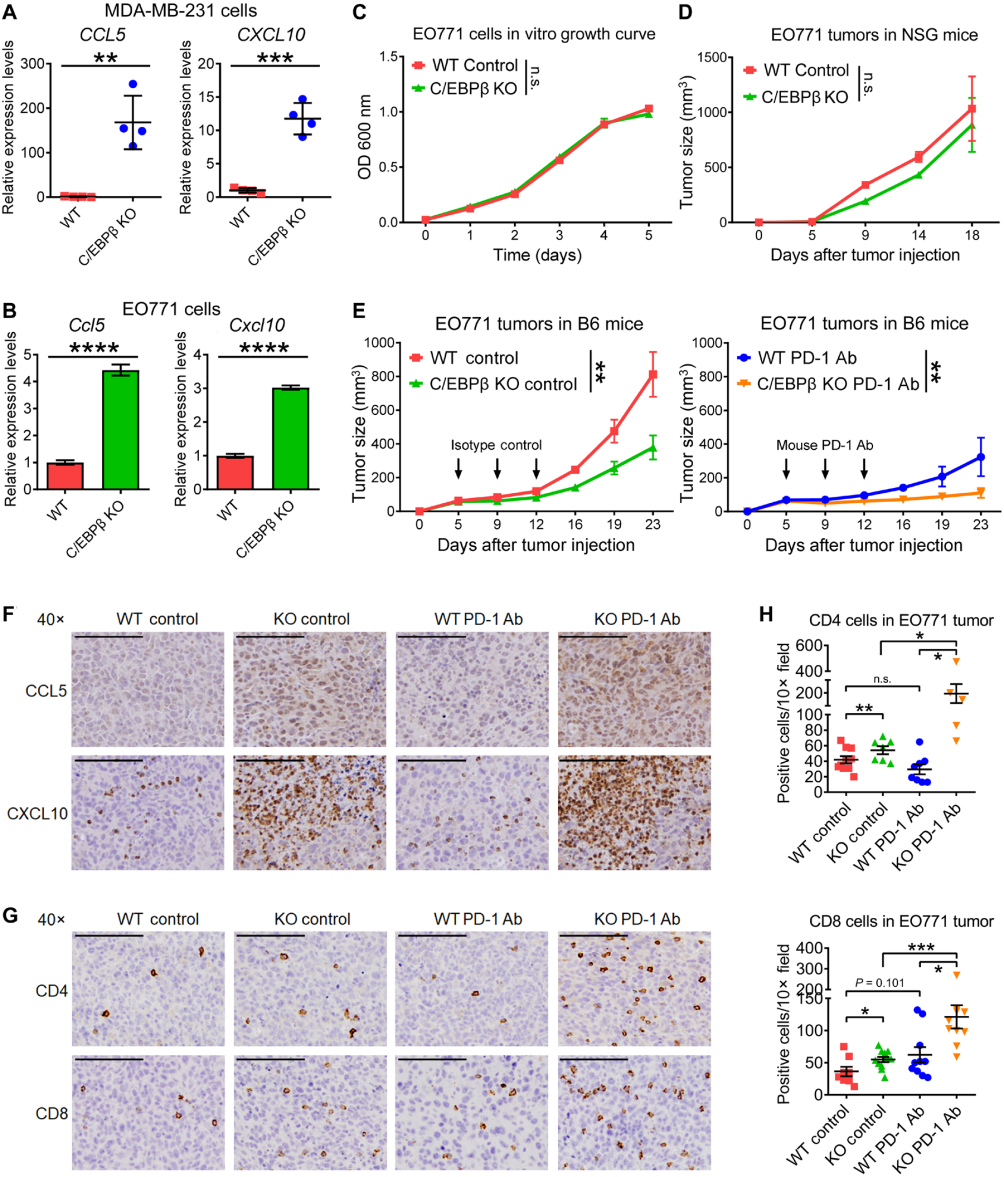
C/EBPβ KO triggers chemokine expression in cancer cells and promotes the efficacy of cancer immunotherapy. (**A** and **B**) C/EBPβ was knocked out in MDA-MB-231 and EO771 cells. The chemokine expression on RNA levels was detected in WT and KO cells by qPCR. Representative data from three independent experiments, means ± SD. (**C**) MTT assay for the in vitro growth curves of WT and C/EBPβ KO EO771 cells [read daily at optical density (OD) at 600 nm]. Representative data from two independent experiments, means ± SEM. (**D**) WT and C/EBPβ KO EO771 cells were injected into the mammary fat pad of NSG mice (*n* = 3). Tumor sizes were observed every 4 to 5 days. Representative data from two independent experiments, means ± SD. (**E**) WT and C/EBPβ KO EO771 cells were injected into the mammary fat pad of WT B6 mice (*n* = 5). Isotype control or PD-1 Ab was intraperitoneally injected twice a week since day 5. Tumor sizes were observed twice a week. The four study groups were conducted in parallel. Individual growth curves for each mouse were shown in fig. S7I. Representative data from two independent experiments, means ± SD. (**F** and **G**) IHC staining of CCL5, CXCL10, and tumor-infiltrated T cells (CD4 and CD8) on WT and C/EBPβ KO EO771 tumor sections from WT B6 mice with or without PD-1 Ab treatment (40×; scale bars, 100 μm). Representative data from two independent experiments. (**H**) Analyses of tumor-infiltrated T cells (positive cell number per 10× field) in WT and C/EBPβ KO EO771 tumors with or without PD-1 Ab treatment. Representative data from two independent experiments, means ± SEM. Statistical analyses: Student’s unpaired *t* test (A, B, and H) and ANOVA and Student’s unpaired *t* test (C to E). *P < 0.05; **P < 0.01; ***P < 0.001; ****P < 0.0001. n.s., not significant.

Because all our findings presented above are based on a DSS or AOM/DSS inflammation-associated model (a tumor prevention model), blocking immune cell infiltration through T_H_17 cells or their cytokines (IL-17 and/or IL-22) markedly reduces inflammation-induced colitis and tumor development. This protective immunity is mediated through C/EBPβ-mediated inhibition of key chemokines in the colon epithelial cells. However, in therapeutic tumor models, inhibition of chemokine expression and exclusion of T cell infiltration have been identified as a major issue for cancer immunotherapy. For example, the so-called cold tumor that has poor responses to cancer immunotherapy is due to the lack of tumor-infiltrating lymphocytes or antigen-specific T cells and other immune cells at tumor sites. Breast cancer generally poorly responds to immune checkpoint therapy (*54*). To address this critical issue, we reasoned that up-regulation of local chemokine expression in the tumor microenvironment may enhance the recruitment of antitumor immune cells to the tumor sites, thus sensitizing cancer cells for immunotherapy. Because our study identified C/EBPβ as a key negative regulator of chemokine expression in epithelial tissues, we hypothesize that KO of C/EBPβ might increase chemokine expression in epithelial-derived cancer cells to recruit T cells to tumor sites. To test this possibility, we knocked out C/EBPβ in MDA-MB-231 cells and found that C/EBPβ KO enhanced the expression of *CCL5* and *CXCL10* by more than 100- and 10-fold, respectively (Fig. 7A and fig. S7A). Similarly, we found that C/EBPβ was highly expressed in mouse EO771, CT26.WT, RM-1, C3.43, and ID-8 cell lines (fig. S7, B and C), and the KO of C/EBPβ markedly enhanced expression of *Ccl5* and *Cxcl10* in these cancer cells (Fig. 7B and fig. S7, D to G).

EO771 cell line is selected for further in vivo study, because it responds to the checkpoint blockade immunotherapy (*55*). We first showed that the in vitro growth rates of WT and C/EBPβ KO EO771 cells were identical (Fig. 7C and fig. S7H). When injected into the immunodeficient NSG (NOD.Cg-*Prkdc^scid^ Il2rg^tm1Wjl^*/SzJ) mice, the growth rates of WT and C/EBPβ KO tumors were also comparable (Fig. 7D). Next, we injected WT and C/EBPβ KO EO771 cells into the mammary fat pad of WT C57BL/6J (B6) mice, with or without PD-1 Ab treatment. Unexpectedly, we found that C/EBPβ KO EO771 tumors grew significantly slower than WT tumors (Fig. 7E), suggesting that C/EBPβ KO EO771 cells are more immunogenic compared with WT EO771 cells. We further monitored the tumor growth for a longer time and found that all the five mice in control WT EO771 group had rapid tumor growth and were euthanized by day 30. By contrast, either C/EBPβ KO or PD-1 Ab treatment could inhibit the tumor growth. Notably, some tumors in these groups (one of five in C/EBPβ KO group and two of five in WT PD-1 Ab treatment group) were shrunk and became very tiny at the endpoint of day 40. The group of mice with C/EBPβ KO EO771 and anti–PD-1 treatment showed three of five tumor regression (fig. S7I). Furthermore, IHC staining of WT and KO EO771 tumors revealed the marked increase in CCL5 and CXCL10 in C/EBPβ KO tumor groups, compared to that in the WT tumor groups (Fig. 7F and fig. S7J), providing key direct evidence that C/EBPβ regulates the in vivo chemokine expression. Consistent with this observation, we also found marked increase in CD4^+^ and CD8^+^ cell infiltration in C/EBPβ KO EO771 tumors, compared to that in the WT tumor groups (Fig. 7, G and H, and fig. S7J). Notably, the combination of C/EBPβ KO and PD-1 Ab treatment resulted in optimal T cell infiltration into the tumors (Fig. 7H). These data suggest that C/EBPβ is a critical negative regulator of chemokine production in tumor cells, and the KO of C/EBPβ up-regulates chemokine expression and T cell recruitment and infiltration, thus sensitizing tumor cells for checkpoint blockade therapy.

### C/EBPβ and STAT3 expression are negatively associated with CCL5 expression, CD8^+^ T cell infiltration, and patient survival across multiple cancer types

**Fig. 8.**
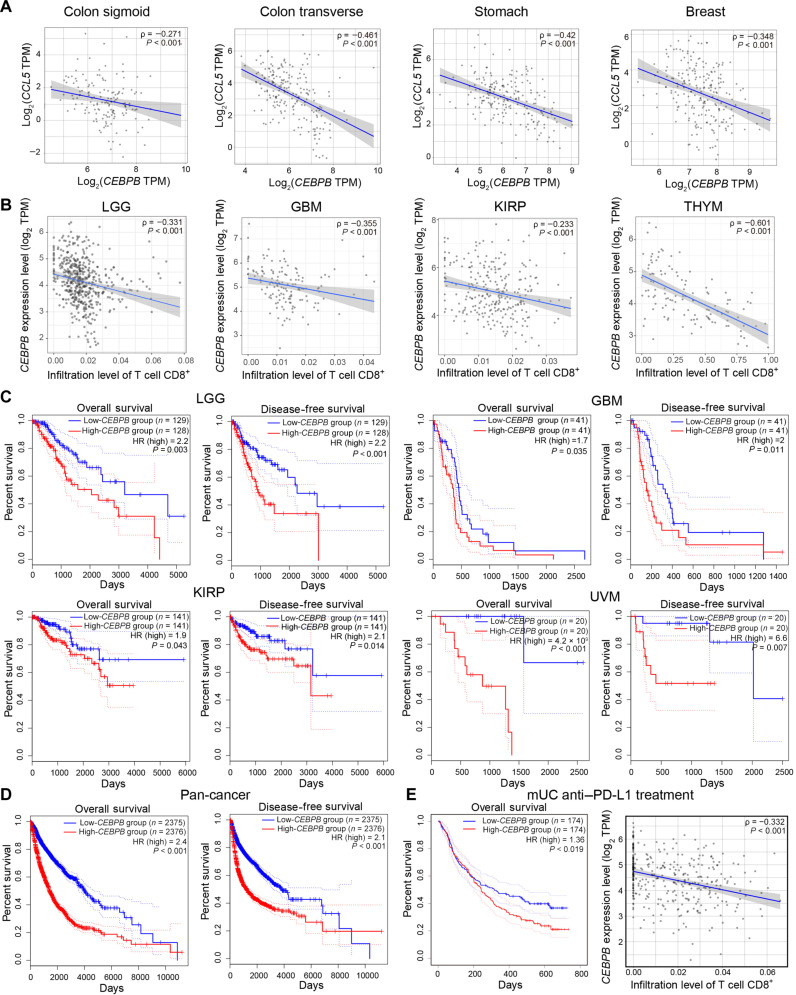
C/EBPβ expression is negatively associated with CCL5 expression, CD8^+^ T cell infiltration, and patient survival across multiple cancer types. (**A**) C/EBPβ expression shows a negative correlation with CCL5 across multiple human tissues. (**B**) C/EBPβ expression negatively correlates with CD8^+^ T cell infiltration across multiple cancer types. (**C**) High C/EBPβ expression is associated with poorer OS and disease-free survival across multiple cancer types. (**D**) High C/EBPβ expression is a risk factor for OS and disease-free survival in pan-cancer analysis. (**E**) C/EBPβ expression is negatively associated with CD8^+^ T cell infiltration and patient survival in patients with metastasis urothelial carcinoma receiving anti–PD-L1 immunotherapy. LGG, lower-grade glioma; GBM, glioblastoma multiforme; KIRP, kidney renal papillary cell carcinoma; THYM, thymoma; UVM, uveal melanoma; mUC, metastatic urothelial carcinoma; HR, hazard ratio.

To evaluate the relevance of our proposed mechanisms in clinical disease conditions, we analyzed patient-derived data from the TCGA (The Cancer Genome Atlas) and Genotype-Tissue Expression (GTEx) databases. Our analysis revealed that C/EBPβ and STAT3 expression negatively correlates with CCL5 across several tissues, including the colon, stomach, breast, and liver, supporting our mechanistic finding that C/EBPβ suppresses chemokine expression (Fig. 8A and fig. S8A). Furthermore, TIMER 2.0 (Tumor Immune Estimation Resource 2.0) analysis demonstrated that high C/EBPβ/STAT3 expression is linked to reduced CD8^+^ T cell infiltration in multiple aggressive cancers (e.g., lower-grade glioma, glioblastoma multiforme, kidney renal papillary cell carcinoma, and testicular germ cell tumors), providing further evidence that the C/EBPβ/STAT3 axis inhibits chemokine expression, thereby limiting cytotoxic T cell recruitment (Fig. 8B and fig. S8B). Elevated C/EBPβ/STAT3 expression correlates with poor disease-free survival and overall survival (OS) across multiple cancer types (Fig. 8C and fig. S8C). Notably, C/EBPβ/STAT3 shows a high pan-cancer hazard ratio for OS, underscoring its broad clinical relevance (Fig. 8D and fig. S8D). These findings suggest that C/EBPβ/STAT3-mediated inhibition of T cell recruitment contributes to poor outcomes in patients with cancer, consistent with our animal experiment results.

Additionally, consistent with our mouse data on immune checkpoint therapy, low C/EBPβ/STAT3 expression correlates with significantly improved survival in patients with metastatic urothelial carcinoma receiving programmed cell death ligand 1 (PD-L1) blockade therapy. This suggests that C/EBPβ may serve as a potential biomarker for predicting response to immune checkpoint inhibitors (Fig. 8E and fig. S8E).

Overall, these findings validate our study with clinical data and highlight the clinical relevance of the C/EBPβ/STAT3-chemokine axis in human disease, connecting it to immune evasion, poor prognosis, and varying treatment responses. While our analysis relies on retrospective datasets, the consistency of these trends across diverse cancer types strengthens their biological and translational significance.

## DISCUSSION

In this study, by using the *Tak1*^Δ*M/*Δ*M*^ mice as a unique CRC-resistant model, we demonstrated that the inhibition of chemokine expression by T_H_17 cytokines (IL-17A and IL-22) and subsequent infiltration of immune cells, particularly neutrophils and CD8^+^ cells, in colon epithelia are the key events for complete resistance to inflammation, tissue damage, and tumorigenesis. Furthermore, we show that the accumulated T_H_17 cells and their cytokine products (IL-17A and IL-22) strongly inhibit DSS-induced expression of multiple chemokine genes, particularly *Ccl5*, *Cxcl5*, and *Cxcl10*, in the colon epithelial cells of the *Tak1*^Δ*M/*Δ*M*^ mice. Mechanistically, we found that C/EBPβ and STAT3 are important regulators for T_H_17 cytokine–mediated control of chemokine expression in the gut epithelial cells. The gut epithelial–specific depletion of C/EBPβ or STAT3 abolishes the protective role of T_H_17 cytokines against colitis progression. To explore a broader utilization of our finding beyond the colon inflammation and cancer models, we identified C/EBPβ as a key regulator of chemokine expression in several types of cancer cells. KO of C/EBPβ in cancer cells markedly reduces tumor growth and enhances immunotherapy of anti–PD-1 treatment in breast cancer models.

Despite of the importance of T_H_17 cells and their cytokines in different tumor models, the key mechanisms that modulate protumor or antitumor immunity in different tumor setting (preventive versus therapeutic tumor models) remain poorly understood. In particular, our knowledge of the downstream of T_H_17 cytokine–mediated signal pathway is limited. Using T_H_17 cell transfer, Ab-mediated cell depletion, and genetic ablation, we sought to dissect the key mechanisms by which T_H_17 cytokines control the downstream signaling events that dictate the fate of immune responses. In this study, we show that the percentages of T_H_17 cells in the intestinal tissues are directly linked to chemokine expression in colon epithelia and the sensitivity of animals to colitis and CRC development. Although the roles of IL-17A and IL-22 in colon cancer are controversial and linked to both tumor-inhibiting and promoting effects, we have provided several lines of evidence that the cytokines protect epithelial barrier function by inhibiting the expression of an array of chemokines, particularly CCL5, CXCL5, and CXCL10. Particularly, the direct administration of exogenous cytokines (IL-17A or IL-22) inhibits chemokine expression in the colon tissues and partially protects the mice against colitis, consistent with the resistant phenotypes observed in the chemokine KO mice.

Inflammatory chemokines mainly function as “gatekeepers” of immunity and inflammation (*45*). In colitis and CRC models, the expression of key chemokines in colon epithelial tissues is critical in directing the trafficking of proinflammatory immune cells. It has been reported that KO of the *Ccr5* gene can ameliorate DSS-induced colitis (*56*), while KO of *Cxcr2* inhibits CRC (*57*). The proinflammatory roles of CCL2 and its key receptor CCR2 have also been reported in colitis models (*58*, *59*). However, these previous studies do not address how these chemokine receptors affect the DSS-induced sensitivity and tumor development. In this study, we first demonstrated that CCL5, CXCL5, and CXCL10 expression was markedly inhibited in colon tissues from T_H_17 cell–transferred WT mice, compared with that in colon tissues from control T cell–transferred mice. Because the *Ccl5^flox/flox^* and *Cxcl10^flox/flox^* mouse strains are not commercially available for epithelial cell–specific depletion, we used traditional KO mice in our further experiments. CXCL5 has been reported to promote tumor progression by recruiting myeloid-derived suppressor cells and neutrophils, which establish an immunosuppressive microenvironment that facilitates angiogenesis and metastasis (*60*). Because *Ccl5^flox/flox^* and *Cxcl5*^−/−^ mice are also not commercially available, we mainly focused on evaluating the function of CCL5 and CXCL10 in our study. We show that both *Ccl5*^−/−^ and *Cxcl10*^−/−^ mice are more resistant to DSS-induced colitis than WT mice by reducing immune cell infiltration, thus maintaining intestinal epithelial barrier function, consistent with previous reports that CCL5 is highly induced and plays a key role in DSS colitis model (*61*, *62*). Therefore, IL-17A– and IL-22–mediated inhibition of chemokine expression in the intestinal epithelia is the key mechanism that blocks the infiltration of immune cells into colon tissues, thus suppressing the tissue damage and maintaining epithelial barrier function and integrity. Thus, our study bridges a critical gap between T_H_17 cytokines and chemokine expression in colon epithelial tissues as a key event, which drives subsequential signaling pathways that control epithelial barrier function and integrity.

Given the essential role of CCL5 and CXCL10 in T_H_17-mediated protection in colitis models, their absence in mice is expected to diminish the therapeutic efficacy of T_H_17 adoptive cell therapy. However, further investigation is needed to determine whether additional chemokines are also required for T_H_17-mediated protection in DSS-induced colitis. Notably, at different models and stages of the disease, the chemokines and subsequent immune cell recruitment have different functional consequences. In AOM/DSS-induced colon cancer model (an early stage of cancer initiation and development model), chemokines recruit immune cells to the colon, which cause inflammation, tissue damage, and eventual carcinogenesis. However, in EO771 breast cancer model (a late stage of established tumor), chemokine-mediated immune cell recruitment may lead to sensitizing cancer cells for immunotherapy.

The CD8^+^ cell levels showed discrepancies between LPL and IEL levels, while other immune cell populations were consistent. At IEL level (by IHC staining), CD8^+^ cell infiltration was significantly decreased in *Tak1*^Δ*M/*Δ*M*^ mice after DSS treatment, consistent with our key findings. However, higher CD8^+^ cells at LPL level (by flow cytometry) in *Tak1*^Δ*M/*Δ*M*^ mice, with or without DSS treatment, suggest that the high levels of CD4^+^ T cells (particularly CD4 T_H_17 cells) within the epithelium have no influence with the CD8^+^ cell at LPL level but may inhibit CD8^+^ T cell infiltration from LP into the epithelial layer, even before DSS treatment. This observation further supports our hypothesis that a potential T_H_17-mediated immunosuppressive environment in *Tak1*^Δ*M/*Δ*M*^ mice restricts CD8^+^ cell migration into the epithelial layer and reduces inflammation during colitis.

It should be noted that the IL-17A–mediated inhibition of chemokine expression is cell-type dependent. For example, IL-17A promotes chemokine expression in many other tissue types, such as skin and lung (*63*–*66*). Anti–IL-17A blockade therapy has shown clinical benefits for psoriasis patients and has recently been approved by the Food and Drug Administration for use in plaque psoriasis (*63*, *64*). By contrast, clinical trials of anti–IL-17A or anti–IL-17RA Abs show no clinical benefits or even exacerbation of Crohn’s disease (*67*). Our findings may explain why anti–IL-17A treatment does not have benefits or even exacerbation of Crohn’s disease, which is caused by excessive proinflammation and immune cell infiltration. Blocking of IL-17A by Ab treatment leads to more chemokine expression, followed by more immune cell infiltration, resulting in exacerbation of Crohn’s disease. Moreover, our findings indicate that both IL-17A and IL-22 mediate the inhibition of chemokine expression in intestinal epithelial cells. IL-22 is produced not only by T_H_17 cells but also by other cell types, such as innate lymphoid cell type 3 (ILC3) and T_H_22 cells (*68*), making its function more complex. Given this broader context, our study focuses on IL-17A signaling. However, future studies using an IL-22 receptor (IL-22R)–deficient mouse model would be valuable in further elucidating the specific role of IL-22 in colitis protection.

To understand the molecular mechanisms by which T_H_17 cells and their cytokines mediate protective function, we generated IL-17RC KO in *Tak1*^Δ*M/*Δ*M*^ mice and observed a completed conversion from resistant to sensitive phenotype upon DSS treatment. This is consistent with a recent report that IL-17RC is a critical determinant of IL-17–mediated response in tumor cells (*69*). IL-17A mediates its cellular effects via a receptor complex composed of IL-17RA and IL-17RC subunits (*38*). Because the cytoplasmic signaling molecule ACT1 is an adaptive molecule to recruit TRAF6 and TRAF3 after IL-17A stimulation (*70*), we generated ACT1 KD HCT116 cells and show that ACT1 KD abolished the ability of IL-17A to inhibit chemokine expression. Further experiments using KO HCT116 cells in the downstream signaling molecules such as C/EBPβ and other molecules revealed that C/EBPβ is a master negative regulator for chemokine expression. IL-17A failed to inhibit chemokine expression in C/EBPβ KO HCT cells. IL-22 performs its functions through a heterodimeric receptor complex containing IL-22R1 and IL-10R2 and a series of downstream signaling molecules including STAT3 (*41*). Here, we identified STAT3 as a key regulator of IL-22 signaling in colon epithelial cells. STAT3 KO HCT116 cells fail to respond to IL-22 for inhibiting chemokine expression. Notably, IL-17R and ACT1 are not required for IL-22 signaling, so the IL-22 signaling in *Tak1*^Δ*M/*Δ*M*^*;Il17rc^−/−^* mice is probably intact. However, we found that, in *Tak1*^Δ*M/*Δ*M*^*;Il17rc^−/−^* mice, IL-22 signaling alone could not compensate for IL-17A signaling deficiency and failed to maintain the protective phenotype. Therefore, IL-17A may play the dominant role in *Tak1*^Δ*M/*Δ*M*^ mice to maintain the resistance against colitis, while IL-22 provides an independent and supportive function.

C/EBPβ protein could not be detected in STAT3 KO HCT116 cells, which is consistent with previous reports that STAT3 interacts with C/EBPβ to stabilize C/EBPβ protein for its inhibition of chemokine expression (*53*). In that study, KD of C/EBPβ also destabilizes STAT3 protein. However, we did not observe any change in STAT3 protein level in the C/EBPβ KO HCT116 cells. Thus, it appears that IL-17A–IL-17R–ACT1 and IL-22–IL-22R–STAT3 signaling pathways converge on the negative master regulator C/EBPβ to inhibit chemokine expression. To further substantiate these finding in physiological conditions, we generated *Cebpb^F/F^;Vil-Cre^+^* and *Stat3^F/F^;Vil-Cre^+^* mice and found that both strains become sensitive to DSS treatment after the administration of IL-17A or IL-22, compared with WT control mice treated with IL-17A or IL-22, suggesting that KO of either C/EBPβ or STAT3 up-regulates chemokine expression, leading to subsequent immune cell infiltration and rendering sensitivity to DSS-induced inflammation. Thus, C/EBPβ and STAT3 function as key negative regulators in T_H_17 cytokine–mediated signaling pathways for inhibiting chemokine expression in colon epithelial cells. Detailed mechanisms of how C/EBPβ and STAT3 control the chemokine axis and further identification of other pivotal downstream factors merit more investigation in our future studies.

PD-1 Ab immunotherapy can enhance the antitumor immunity of T cells in multiple aspects. Besides promoting proliferation and inhibiting exhaustion that are related to the tumor-infiltrated T cell number, PD-1 Ab also enhances T cell activation, cytokine production, and direct tumor-killing ability (*71*). Although T cell number did not significantly increase in PD-1 Ab treated WT tumors than untreated WT tumors, PD-1 Ab may perform its function by enhancing T cell activation and killing ability. Beside T cells, PD-1 Ab therapy can also modulate other key immune cells in the tumor microenvironment, such as natural killer cells and macrophages, and mediate the antitumor immunity (*72*, *73*). Notably, EO771 tumor model is selected because PD-1 Ab therapy can only partially inhibit the tumor growth (*55*). In our study, we show that the chemokine induction by C/EBPβ KO can trigger the immune cell infiltration and markedly increase the efficacy of PD-1 Ab therapy.

Our results suggest that inhibition of chemokine induction by T_H_17 and their cytokines through C/EBPβ-mediated mechanism renders resistance to DSS-induced tissue damage and shows protective role in maintaining tissue barrier and integrity by blocking immune cell infiltration into colon epithelial tissues in the preventive tumor model. However, inhibition of chemokine expression and exclusion of T cell infiltration may have an opposite role for immunotherapy in preexisting or therapeutic tumor models. The ICB therapies, such as PD-1 Ab, have shown impressive clinical benefits in many types of cancer; however, most patients with cancer do not respond to ICB therapy due to lack of T cell infiltration at tumor sites (*74*). Several important molecules and signaling pathways, including β-catenin, phosphatase and tensin homolog (PTEN), and TGF-β, have been shown to play a role in the exclusion of T cell infiltration into tumor tissues (*75*–*77*). A recent study shows that C/EBPΔ, as an important family member of C/EBPβ, suppresses the expression of macrophage-associated chemokines and regulates the immunotherapy efficacy in breast cancer by controlling macrophage infiltration in the tumor microenvironment (*78*). Our study identified STAT3 and C/EBPβ as key regulators to inhibit key chemokine expression, thus excluding T cell infiltration into tumor sites. It has been reported that STAT3 is a key regulator involved in the T cell exclusion (*79*). Based on the analysis of TCGA database with The Human Protein Atlas tool (www.proteinatlas.org/ENSG00000172216-CEBPB/pathology/colorectal+cancer), the high level of C/EBPβ expression is inversely correlated with patient survival in colon cancer. To explore a broader utilization of our finding in other cancer types, particularly ICB-responsive models, we knocked out C/EBPβ in murine EO771 and human MDA-MB-231 breast cancer cells and showed that the KO of C/EBPβ could markedly enhance chemokine expression and promote the tumor infiltration of T cells, which, in turn, enhances the efficacy of PD-1 Ab immunotherapy. Thus, C/EBPβ KO could sensitize tumor cells for ICB immunotherapy.

To further validate our findings in clinical disease conditions, we analyzed patient-derived data from the TCGA and GTEx databases. C/EBPβ and STAT3 expression negatively correlates with CCL5 across multiple human tissues, while elevated C/EBPβ/STAT3 expression is associated with reduced CD8^+^ T cell infiltration and poor survival across various cancer types. Notably, low C/EBPβ/STAT3 expression is linked to significantly improved survival in patients with metastatic urothelial carcinoma receiving PD-L1 blockade therapy, suggesting that C/EBPβ/STAT3 may serve as a potential biomarker for predicting response to ICB therapies. Now, ST101, a peptide antagonist targeting C/EBPβ, is in phase 1/2 clinical trials for advanced solid tumors, including glioblastoma, breast cancer, melanoma, and other refractory malignancies. Additionally, Napabucasin (BBI608), a small-molecule STAT3 inhibitor, has advanced to phase 3 trials (*80*). Recent results from a phase 3 monotherapy trial highlight its potential efficacy in advanced colorectal cancer (*81*). Given the role of C/EBPβ/STAT3 in immune evasion, targeting these pathways could enhance T cell infiltration and improve response rates to PD-1/PD-L1 inhibitors. This combination strategy holds more significant promise as a therapeutic approach for multiple aggressive cancer types.

In summary, our work provides significant scientific advance and illustrates a previously unrecognized mechanism by which T_H_17 cells and their cytokines play a protective role in DSS-induced inflammation and tumor development through inhibition of key chemokines and subsequent immune cell infiltration. Mechanistically, we show that IL-17R–ACT1 and IL-22R–STAT3 signaling pathways converge to the key negative regulator C/EBPβ to inhibit chemokine expression in colon epithelial cells, which prevents the pathologic breakdown of epithelial barrier in inflammatory colitis and colon cancer development. Specific KO of C/EBPβ in the colon tissue results in a sensitive phenotype to DSS treatment. Consistently, C/EBPβ KO in breast cancer cells markedly reduces tumor growth and enhances sensitivity to ICB immunotherapy. Thus, our findings have identified a potential therapeutic target for cancer immunotherapy through regulation of chemokine expression and immune cell infiltration.

## MATERIALS AND METHODS

### Animals and in vivo procedures

As described previously (*16*, *37*), *Tak1^flox/flox^* mice (gift from M. Schneider, Baylor College of Medicine) were crossed with *Lyz2-Cre* mice (the Jackson Laboratory, catalog no. JAX:004781) to generate the *Tak1*^Δ*M/*Δ*M*^ mouse strain. C57BL/6 (catalog no. JAX:000664), *Ccl5^−/−^* (catalog no. JAX:005090), *Cxcl10^−/−^* (catalog no. JAX:006087), *Il1r1^−/−^* (catalog no. JAX:003245), *Il6^−/−^* (catalog no. JAX:002254), and *Rorc^−/−^* (catalog no. JAX:007572) mice were purchased from the Jackson Laboratory. *Il17-GFP^+^* strain is a gift from S. Durum (National Institutes of Health). *Il17rc^−/−^* strain is a gift from X. Li (Cleveland Clinic). *Tak1*^Δ*M/*Δ*M*^ mice were crossed with specific mice to generate *Tak1*^Δ*M/*Δ*M*^*;Il1r1^−/−^*, *Tak1*^Δ*M/*Δ*M*^*;Il6^−/−^*, *Tak1*^Δ*M/*Δ*M*^*;Rorc^−/−^*, *Tak1*^Δ*M/*Δ*M*^*;Il17rc^−/−^*, and *Tak1*^Δ*M/*Δ*M*^*;Il17-GFP^+^* strains. C57BL/6 mice were used as WT controls for *Ccl5^−/−^* and *Cxcl10^−/−^* mice. The littermate control mice (*Tak1^flox/flox^;Lyz2-Cre^−^*) were used as WT controls for *Tak1*^Δ*M/*Δ*M*^ mice; they were weaned at 3 weeks old and housed separately from *Tak1*^Δ*M/*Δ*M*^ mice after genotyping. *Cebpb^flox/flox^* (catalog no. JAX:032282), *Stat3^flox/flox^* (catalog no. JAX:016923), and *Vil-Cre* (catalog no. JAX:004586) mice were purchased from the Jackson Laboratory and crossed to generate *Cebpb^F/F^;Vil-Cre^+/+^* and *Stat3^F/F^;Vil-Cre^+/+^* mice. For EO771 tumor studies, C57BL/6 (catalog no. JAX:000664) and NSG (catalog no. JAX:005557) mice were directly ordered from the Jackson Laboratory for experiments. All the genotyping primers were listed in table S1.

As described previously (*16*), in all experiments, 7- to 9-week-old female mice were used unless specifically described. All the mice were housed under a 12-hour:12-hour light:dark cycle and maintained in specific pathogen–free facilities that are accredited by Association for Assessment and Accreditation of Laboratory Animal Care International. All animal studies were approved by the Institutional Animal Care and Use Committee of Houston Methodist Research Institute and University of Southern California. The animal numbers per group were statistically calculated to achieve >90% power to detect a difference between the groups with a significance level (α) of 0.01, or confidence interval of 99%, using a two-sided, two-sample *t* test.

In acute colitis model, mice were administered with 5% DSS (molecular weight, 36 to 50 kDa; MP Biomedicals, catalog no. 02160110) in drinking water for 5 days, followed by regular water for 2 weeks. In some experiments, the mice were treated with 2.5% DSS, which is not lethal to the mice but will cause a milder colitis with consistent phenotypic changes in different mouse strains as treated with 5% DSS. This allows us to collect the tissues after day 7.

In colon cancer model, mice were intraperitoneally injected with AOM (Sigma-Aldrich, 10 mg/kg body weight, catalog no. A5486) on day 0 and provided with 2% DSS through drinking water on days 5 to 10, 24 to 29, and 43 to 48, then euthanized on day 80 for colon tumor analysis. For colon chemokine expression test by real-time qPCR, WT, *Tak1*^Δ*M/*Δ*M*^, and various DKO mice were treated with water control or 2.5% DSS for 5 days, and the tissues were harvested on day 8.

For the depletion of CD4^+^ cells, Ab against L3T4 [300 μg per injection, catalog no. TIB-207, American Type Culture Collection (ATCC)] was intraperitoneally injected every 3 days starting from day −1. The animals were treated with DSS from days 0 to 5.

For T_H_17 cell transfer experiment, intestinal LPLs were isolated from *Tak1*^Δ*M/*Δ*M*^*;Il17-GFP^+^* mice, enriched with untouched mouse CD4 cell kit, and then stained with PE-CD4 Ab. The control CD4^+^ cells (PE^+^/GFP^−^) and T_H_17 cells (PE^+^/GFP^+^) were sorted and injected intraperitoneally to WT recipients on day −1 (1 million cells per mouse). The mice were treated with DSS from days 0 to 5.

To test the protective function of T_H_17 cytokines in WT mice, control buffer (0.1% BSA), recombinant mouse IL-17A (catalog no. 210-17, PeproTech), and mouse IL-22 (catalog no. 210-22, PeproTech) were intraperitoneally injected every 3 days since day −1 (0.5 μg per injection). The animals were treated with DSS from days 0 to 5.

For EO771 tumor studies, WT and C/EBPβ KO EO771 cells [0.25 million cells in 50 μl of phosphate-buffered saline (PBS) and 50 μl of Matrigel] were injected into the mammary fat pad of WT B6 or NSG mice. For WT B6 mice, mouse PD-1 Ab (BE0146, BioXCell, 300 μg per injection) or isotype control (BE0089, BioXCell, 300 μg per injection) was intraperitoneally injected twice a week since day 5. Tumor sizes were observed twice a week.

### Cell lines

HCT116 (ATCC, catalog no. CCL-247), MDA-MB-231 (ATCC, catalog no. HTB-26), EO771 (ATCC, catalog no. CRL-3461), and RM-1 (ATCC, catalog no. CRL-3310) cells were cultured in Dulbecco’s modified Eagle’s medium (DMEM; Gibco) supplemented with 10% heat-inactivated fetal bovine serum (FBS). CT26.WT cells (ATCC, catalog no. CRL-2638) were cultured in RPMI 1640 medium (Gibco) supplemented with 10% FBS (Hyclone). C3.43 cells (gift from M. Kast, University of Southern California) were cultured in DMEM with 10% FBS and 0.1% 2-mercaptoethanol (Thermo Fisher Scientific, catalog no. 21985023). ID-8 cells (Millipore, catalog no. SCC145) were cultured in DMEM with 4% FBS, insulin (5 μg/ml), transferrin (5 μg/ml), and sodium selenite (5 ng/ml). All these cell lines were supplemented with penicillin (100 U/ml, Thermo Fisher Scientific) and streptomycin (100 μg/ml, Thermo Fisher Scientific) and maintained at 37°C in a humidified atmosphere with 5% CO_2_. All cell lines were regularly tested for mycoplasma and maintained negative. Cell line authenticity was checked regularly on the basis of imagery of morphology and descriptions of growth from ATCC and publications. Human cytokines for cell treatment were ordered from PeproTech: IL-17A (catalog no. 200-17), IL-22 (catalog no. 200-22), IL-1β (catalog no. 200-01B), and IL-6 (catalog no. 200-06).

### Histology, IHC, and microscopy

Fresh colon pieces were fixed with 3.7% formalin for 24 hours and then sent to USC School of Pharmacy Histology Laboratory for further processing and hematoxylin and eosin staining. IHC staining was performed following previously published protocol (*82*). Briefly, unstained tissue sections were deparaffinized in xylene, rehydrated in graded ethanol solutions, and washed in tap water. Antigen retrieval was achieved by boiling the slides in a pressure cooker for 3 min in a citrated buffer (10 mM trisodium citrate, pH 6.0). After 10 min treatment with 3% H_2_O_2_, tissue sections were blocked with 5% normal goat serum in TBST (Tris-buffered saline with 0.1% Tween 20 detergent) for 1 hour at room temperature and incubated with primary Abs at 4°C overnight and with EnVision Polymer–horseradish peroxidase (HRP) secondary Abs (Dako) at room temperature for 30 min. After the application of DAB chromogen (Vector Laboratories, catalog no. SK-4100), tissue sections were stained with hematoxylin, dehydrated, and mounted. Images were acquired using the Olympus BX61 microscope along with DP71 digital camera (Olympus). All the IHC Abs used in this study were listed in table S2.

After the IHC staining of Ki-67, slides were read under microscope, and fields were randomly selected for analysis. Pictures were taken at ×40 magnification. The positive and negative cell numbers were counted in each field. For each mouse, four to five fields were used for quantification, with the animal number of three to five for each group. For immune cells, the cell numbers were calculated, and the positive cell percentages were used for statistical analyses. For chemokines, the H-score was used for quantification, which captures both the staining intensity and the proportion of biomarkers from the IHC images. The staining intensity has four levels: negative (0), weak (1), moderate (2), and strong (3). The H-score was calculated by multiplying the intensity value (0 to 3) with the percentage of positive area (0 to 100), thus comprising values between 0 and 300.

### Real-time qPCR analysis

As described previously (*82*), total RNA was isolated from cultured cells or mouse tissues by TRIzol Reagent (Invitrogen), and the first-strand cDNA was generated from total RNA using SuperScript IV Reverse Transcriptase (Thermo Fisher Scientific), following the manufacturers’ instructions. Real-time qPCR was performed using iTaq Universal SYBR Green Supermix (Bio-Rad) and specific primers on the Applied Biosystems QuantStudio 6 Flex Real-Time PCR system (Thermo Fisher Scientific). The results were analyzed using QuantStudio Software v.1.3 (Thermo Fisher Scientific). Relative expression of target genes was normalized with mouse or human *GAPDH* primers. All the real-time qPCR primers were listed in table S3.

### Protein expression analysis

The whole-cell extracts of cultured cells were isolated in the low-salt lysis and extraction buffer (50 mM Hepes, 150 mM NaCl, 1 mM EDTA, 10% glycerol, 1.5 mM MgCl_2_, and 1% Triton X-100), supplemented with protease inhibitor cocktail (Roche) and PhosSTOP phosphatase inhibitors (Roche), as previously described (*83*). Cell debris was removed by centrifugation for 3 min at 12,000*g* at 4°C in a microcentrifuge. Lysate was mixed with 5× SDS loading buffer and heated for 5 min at 100°C. Prepared samples were resolved by 10% SDS–polyacrylamide gel electrophoresis and transferred to polyvinylidene difluoride membranes (Bio-Rad). The membranes were blocked for 1 hour at room temperature using blocking buffer (5% nonfat dried milk in TBST). Blots were incubated with appropriate primary Abs (diluted at 1:1000) and HRP-conjugated secondary Abs (diluted at 1:3000). Protein expression was normalized to β-actin Ab (diluted at 1:3000). HRP was detected using chemiluminescent HRP substrate (Millipore). Digital images were acquired with the ChemiDoc XRS+ System and analyzed by Image Lab v.5.1 (Bio-Rad). All the Western blotting Abs used in this study were listed in table S2.

### Generation of KD and KO cell lines

Human ACT1 and nonspecific control lentiviral short hairpin RNAs (shRNAs) were obtained from GIPZ lentiviral shRNA library (GE Dharmacon, catalog no. RHS6037). HCT116 cells were transduced with lentivirus harboring different shRNAs. Before use, shRNA-positive cells were selected for 1 week by culturing in medium containing puromycin (2 μg/ml).

Human gene KO (C/EBPβ, STAT3, Akt1, ERK1, ERK2, PI3K-p110γ, and P38) in HCT116 and MDA-MB-231 cells were performed using CRISPR technology. Briefly, cells were transduced with Lenti-CRISPR-V2-RFP (red fluorescent protein) lentivector and cultured for 3 to 5 days. Cells with robust expression of RFP were sorted using a BD FACSAria cell sorter (BD Biosciences) and subjected to generate the Cas9-overexpressing monoclonal cell lines by limited dilution (30 cells per 96-well plate). The Cas9-overexpressing cell clones were picked up and expanded. The lenti-puro-sgRNAs (single guide RNAs) for the target genes were purified from a commercial human sgRNA library (Thermo Fisher Scientific, LentiArray Human CRISPR Library). Cells with highly expressed Cas9 were further transduced with a mixture of four sgRNAs for each gene to generate the KO polyclonal pool of stable cell lines. The sgRNA-positive cells were selected by puromycin (2 μg/ml; Sigma-Aldrich) for 5 days and seeded into 96-well plates for isolating the KO clones by limited dilution. After 2 to 3 weeks, the single clones were picked up for expansion and cultured with puromycin for at least 1 week. The individual gene KO monoclonal cells were identified by Western blotting with the corresponding Ab. The sgRNA sequences for these target genes are listed in table S4.

Mouse C/EBPβ KO in EO771, CT26.WT, RM-1, C3.43, and ID-8 cells were performed by using electroporation. Cas9-sgRNA ribonucleoprotein (RNP) mixture was prepared before electroporation by adding 5 μg of Cas9 protein (TrueCut Cas9 Protein v2, Invitrogen, catalog no. A36498) and a combination of three C/EBPβ sgRNAs (TrueGuide Synthetic sgRNA, 100 pmol per shRNA, CRISPR62684_SGM, CRISPR62681_SGM, CRISPR62691_SGM, Invitrogen, catalog no. A35533) in 50 μl of Opti-MEM medium (Gibco). The mixture was incubated at room temperature for 10 min. Resuspended mouse cancer cells were washed twice with Opti-MEM medium. After cell counting, 0.5 million cells in 50 μl of Opti-MEM were used for each reaction, and mixed with the RNP mixture. Cells were electroporated in the electroporation cuvette with square wave mode using BIO-RAD Gene Pulser Xcell Total System (500 μF, 2 ms, one pulse). The voltage is 160 V for EO771, CT26.WT, RM-1, and C3.43 cells and 260 V for ID-8 cells. Electroporated cells were transferred to medium and cultured at 37°C in a humidified atmosphere with 5% CO_2_.

### Isolation of intestinal LPLs

As described previously (*16*), the fat, connective tissue, Peyer’s patches, and feces were removed from colon. The colons were then cut into ~0.5-cm pieces and incubated in 2 mM EDTA/PBS/FBS for 30 min at 37°C with 200 rpm shaking. After EDTA treatment, the small intestine and colon pieces were washed in PBS/FBS solution and incubated in collagenase-IV (0.5 mg/ml; Sigma-Aldrich, catalog no. C5138)/RPMI 1640/FBS for 90 min at 37°C with shaking. After collagenase digestion, the supernatant that contains LP cells was centrifuged, and the cell pellets were resuspended in 44% Percoll (GE Healthcare, catalog no. 17089101)/RPMI 1640/FBS. The 44% Percoll/cell suspension was slowly added onto the surface of 67% Percoll/RPMI 1640/FBS. After centrifugation, the LP cells were at the 44 to 67% interface.

### Flow cytometry and sorting

As described previously (*16*), for surface marker staining, cells were directly stained in a cocktail of fluorochrome-conjugated Abs (eBioscience or BD Biosciences) for 30 min on ice, washed twice in PBS/FBS, and resuspended in PBS/FBS for flow cytometry. For intracellular staining, cells were activated with phorbol 12-myristate 13-acetate, ionomycin, and protein transport inhibitor for 4 hours at 37°C. Then, the cells were resuspended in Fix/Perm buffer (BD Biosciences, catalog no. 554715) and treated for 20 min on ice, washed with Perm/Wash buffer from the same kit, and stained with the Ab cocktail for 30 min on ice. After washing twice, cells were resuspended in PBS/FBS for flow cytometry. BD LSR II analyzer was used to distinguish different subsets. For the sorting of T_H_17 cells, intestinal LPLs were isolated from *Tak1*^Δ*M/*Δ*M*^*;Il17-GFP^+^* mice, enriched with the Untouched Mouse CD4 Cells Kit (Thermo Fisher Scientific, catalog no. 11415D) following the manufacturer’s instruction and then stained with PE-CD4 Ab for 10 min at room temperature. After washing twice with RPMI 1640/FBS, cells were filtered with 40-μm strainer and resuspended in RPMI 1640/FBS for sorting. BD FACSAria II sorter were used to acquire the control CD4^+^ (PE^+^/GFP^−^) and T_H_17 population (PE^+^/GFP^+^). All the flow cytometry Abs used in this study were listed in table S2.

### Enzyme-linked immunosorbent assay

As described previously (*16*), the plates were coated with capture Abs [eBioscience or Medical & Biological Laboratories Co., Ltd. (MBL)] at 4°C overnight and blocked with PBS/BSA for an hour at room temperature. Samples and standards were incubated in the plates for 2 hours at room temperature. The plates were treated with biotinylated detection Abs (eBioscience or MBL) for 1 hour and HRP chromogen (Thermo Fisher Scientific, catalog no. N200) for 30 min at room temperature. Tetramethylbenzidine (Sigma-Aldrich, T5525) was added to the plates for 5 to 10 min, and the reactions were stopped with an equal volume of 2 M H_2_SO_4_. The absorbance of each well was read at 450 nm on BioTek Synergy 2 microplate reader. The kits for detecting mouse IL-17F (catalog no. 88-7472-88), IL-21 (catalog no. 88-8210-88), and IL-22 (catalog no. 88-7422-88) were ordered from Thermo Fisher Scientific, and the enzyme-linked immunosorbent assay was performed following the manufacturer’s instruction.

### Cell viability assay

As previously reported (*84*), the MTT [3-(4,5-dimethylthiazol-2-yl)-2,5-diphenyltetrazolium bromide] assay was used to determine the in vitro growth rate of cancer cells. Briefly, cells were cultured in 96-well plates at 1000 cells per well and incubated at 37°C with 5% CO_2_. MTT solution (Sigma-Aldrich, catalog no. M2128) was prepared in PBS at 5 mg/ml. At each time points (days 0 to 5), 20 μl of MTT solution was added into the 200 μl of cell culture medium. After 4-hour incubation at 37°C, the medium was discarded and 100 μl of DMSO was added into each well. The 96-well plates were then covered with aluminum foil and shaken for 10 min (360 rpm) at room temperature. The absorbance was read at 600 nm on BioTek Synergy 2 microplate reader. The direct reading values at optical density at 600 nm were shown in Fig. 7C. The data were further normalized with the value on day 0 for the relative fold change in each group, which were shown in fig. S7H.

### Clinical data analysis

To assess the correlation between *CEBPB* and *STAT3* expression and CD8^+^ T cell chemokine *CCL5* expression in vivo, we obtained transcriptome data of normal human tissues from GTEx Analysis V6 (www.gtexportal.org/). Moreover, to assess the prognostic impact of *CEBPB* and *STAT3* expression in tumor patients receiving immunotherapy, we gathered data from the phase 2 IMvigor210 trial (*85*), which included patients with locally advanced or metastatic urothelial carcinoma treated with an anti–PD-L1 agent (atezolizumab). OS and transcriptomic data were obtained via the IMvigor210CoreBiologies R package (*77*). For both datasets, gene expression was normalized to transcripts per million (TPM). CD8^+^ T cell infiltration was inferred from normalized gene expression data using TIMER2.0 (*86*). The correlation between expression of different genes or between gene expression and CD8^+^ T cell infiltration was assessed via Spearman’s rank correlation analysis. Survival analysis was performed after stratifying patients on the basis of median gene TPM levels. Kaplan-Meier survival analysis with a log-rank test assessed the prognostic significance, while Cox proportional hazards regression estimated hazard ratios. The survival analysis of *CEBPB* and *STAT3* expression levels across various cancer types was performed using GEPIA2 (*87*) on the basis of data from TCGA database.

### Statistical analysis

Descriptive statistics, including means, SDs, medians, and ranges, were computed for each group and analyzed with Student’s *t* test or for multiple comparisons, with analysis of variance (ANOVA). Data are presented as means ± SD or means ± SEM, as described in the figure legends. Differences in mice survival were evaluated with Mantel-Cox log-rank test. The sample size for each experiment is included in Results and associated figure legends. All analyses were performed with GraphPad Prism 5 (GraphPad Software, La Jolla, CA). *P* values of <0.05 were considered significant.

## Supplementary Materials

### This PDF file includes:

Figs. S1 to S8 Tables S1 to S4 References
