# Supplementary material for: TH17 cells regulate chemokine expression in epithelial cells through C/EBPβ and dictate host sensitivity to colitis and cancer immunity
Source: Sci Adv. 2025 Aug 1;11(31):eads3530. doi: 10.1126/sciadv.ads3530 (PMC12315988; doi:10.1126/sciadv.ads3530)
Supplement: Supplementary file 1 — Figs. S1 to S8 Tables S1 to S4 References [file sciadv.ads3530_sm.pdf]

Science Advances
AAAS

Supplementary Materials for

T_H17 cells regulate chemokine expression in epithelial cells through C/EBPβ and dictate host sensitivity to colitis and cancer immunity

Changsheng Xing *et al.*

Corresponding author: Rong-Fu Wang, rongfuwa@usc.edu

Sci. Adv. **11**, eads3530 (2025)
DOI: 10.1126/sciadv.ads3530

This PDF file includes:

Figs. S1 to S8
Tables S1 to S4
References

Figure S1

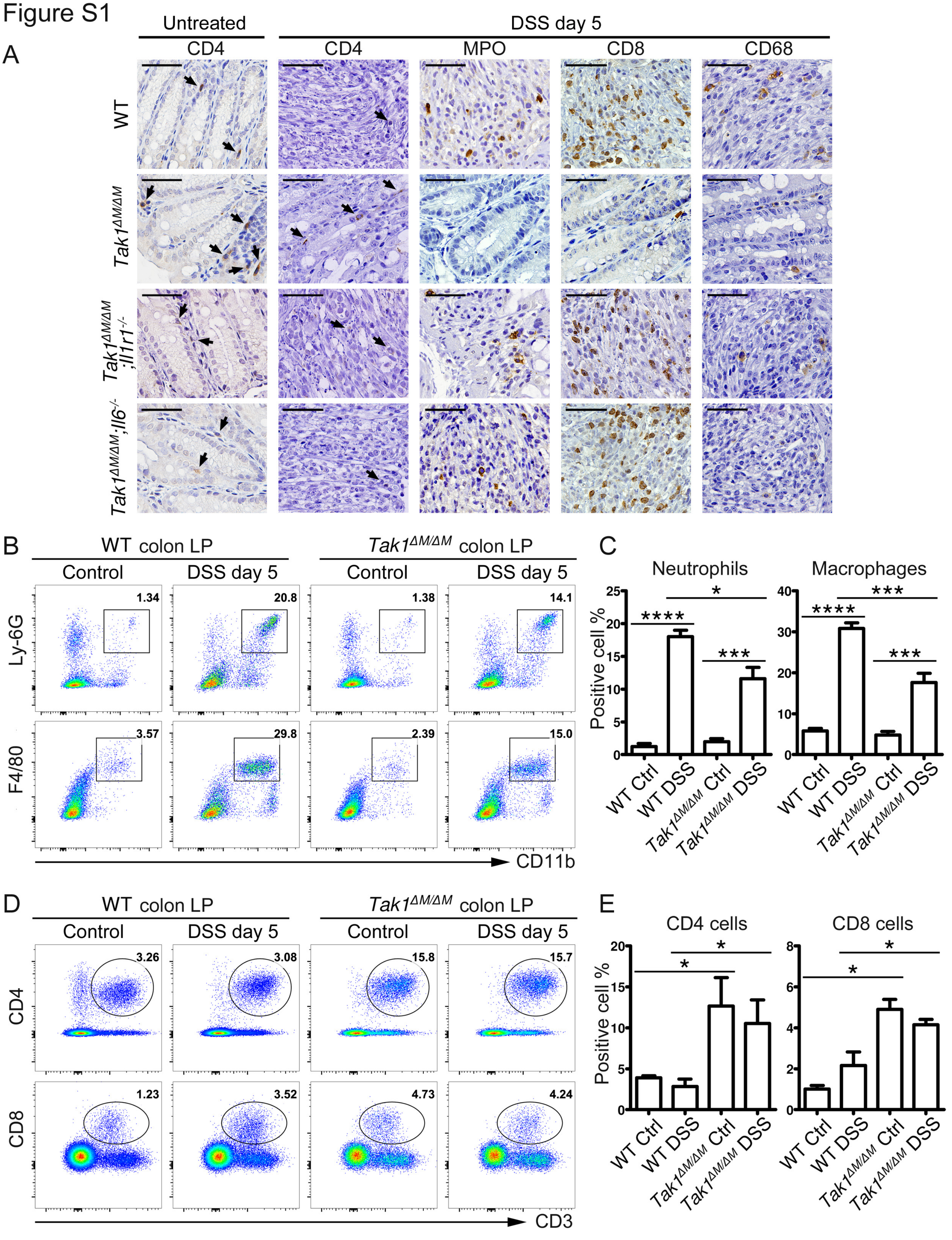
A
Untreated
DSS day 5
CD4
CD4
MPO
CD8
CD68
WT
Tak1ΔM/ΔM
Tak1ΔM/ΔM ;Il1r1-/-
Tak1ΔM/ΔM;Il6-/-
B
WT colon LP
Tak1ΔM/ΔM colon LP
Control
DSS day 5
Control
DSS day 5
Ly-6G
F4/80
CD11b
1.34
20.8
1.38
14.1
3.57
29.8
2.39
15.0
C
Neutrophils
Macrophages
Positive cell %
WT Ctrl
WT DSS
Tak1ΔM/ΔM Ctrl
Tak1ΔM/ΔM DSS
D
WT colon LP
Tak1ΔM/ΔM colon LP
Control
DSS day 5
Control
DSS day 5
CD4
CD8
CD3
3.26
3.08
15.8
15.7
1.23
3.52
4.73
4.24
E
CD4 cells
CD8 cells
Positive cell %
WT Ctrl
WT DSS
Tak1ΔM/ΔM Ctrl
Tak1ΔM/ΔM DSS

Fig. S1. Infiltration of immune cells into colon tissues is closely linked to the sensitivity of mice to DSS treatment. (**A**) Lower magnification IHC images for the data in Figure 1A. IHC staining of CD4, CD8, CD68 (macrophage/monocyte), and MPO (neutrophil) (20X) on colon sections from WT, *Tak1*$^{ΔM/ΔM}$, and double KO mice with or without DSS treatment (scale bar: 100 μm). Arrows were added to indicate the positive $CD4^+$ cells in the IHC images. (**B-C**) WT and *Tak1*$^{ΔM/ΔM}$ mice were treated with water control or DSS for 5 days. On day 7, immune cells were isolated from colon LP. (**B**) For each sample, the granulocyte population was gated from the FSC-A/SSC-A plot, followed by the single cell gating in FSC-A/FSC-H plot. Then the neutrophils ($CD11b^+$/Ly-$6G^+$) and macrophages ($CD11b^+$/F4/80^+) were analyzed by flow cytometry. (**C**) Analyses of positive cell percentage. Representative data from three independent experiments. (**D-E**) WT and *Tak1*$^{ΔM/ΔM}$ mice were treated with water control or DSS for 5 days. On day 7, immune cells were isolated from colon LP. (**D**) For each sample, the lymphocyte population was gated from the FSC-A/SSC-A plot, followed by the single cell gating in FSC-A/FSC-H plot. Then the $CD4^+$ ($CD3^+$/$CD4^+$) and $CD8^+$ ($CD3^+$/$CD8^+$) T lymphocytes were analyzed by flow cytometry. (**E**) Analyses of positive cell percentages. Representative data from three independent experiments. Statistical analyses: Student's unpaired t test (C and E). *$p<0.05$; ***$p<0.001$; ****$p<0.0001$.

Figure S2

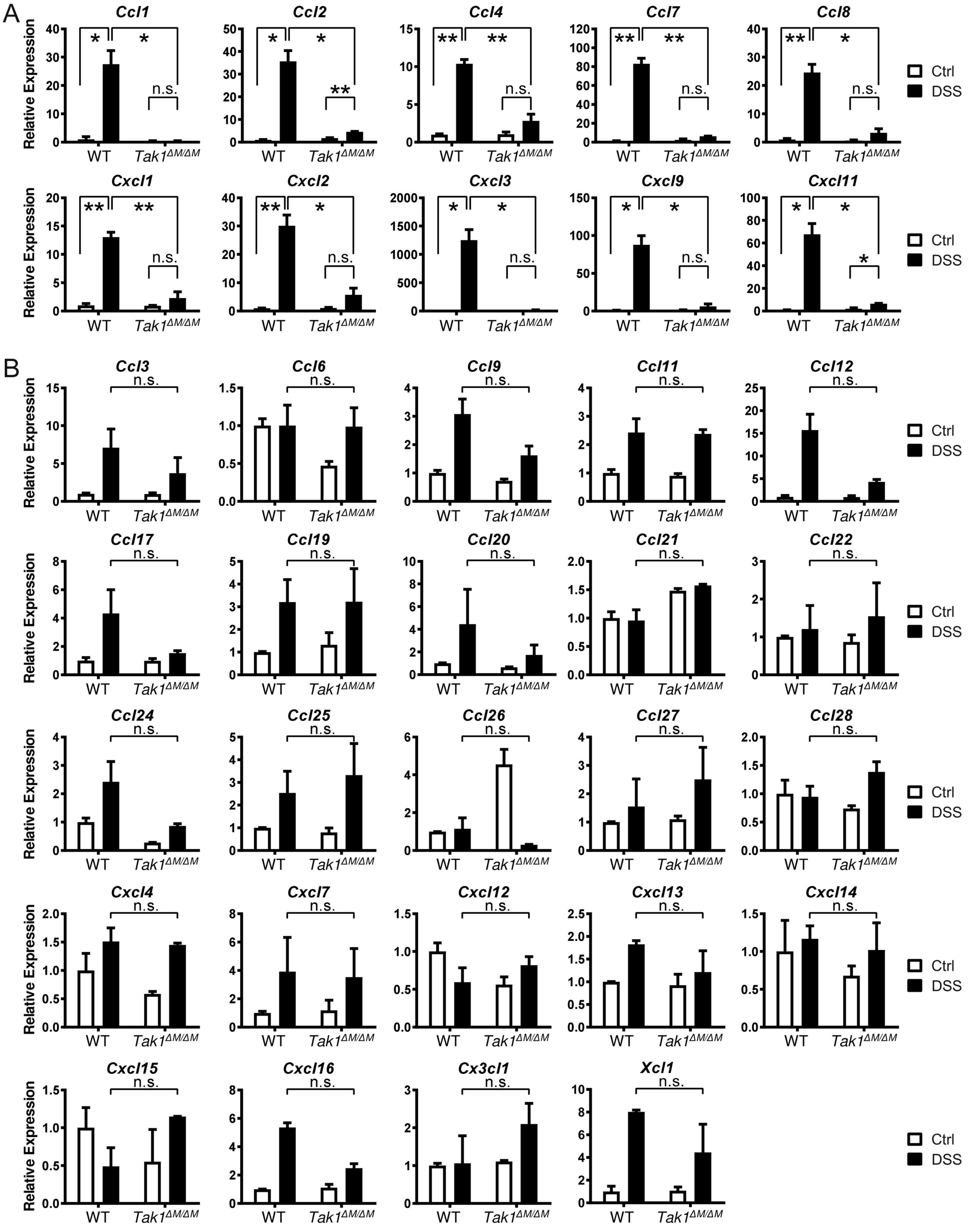

A
Ccl1
Ccl2
Ccl4
Ccl7
Ccl8
Cxcl1
Cxcl2
Cxcl3
Cxcl9
Cxcl11
B
Ccl3
Ccl6
Ccl9
Ccl11
Ccl12
Ccl17
Ccl19
Ccl20
Ccl21
Ccl22
Ccl24
Ccl25
Ccl26
Ccl27
Ccl28
Cxcl4
Cxcl7
Cxcl12
Cxcl13
Cxcl14
Cxcl15
Cxcl16
Cx3cl1
Xcl1
Relative Expression
WT
Tak1ΔM/ΔM
n.s.
Ctrl
DSS

Fig. S2. Chemokine expression is inhibited in the colon tissues of colitis-resistant mice. WT and *Tak1*$^{\Delta M/\Delta M}$ mice were treated with water control or 2.5% DSS for 5 days. On day 8, chemokine expression in the colon was measured by qPCR. (**A**) Selected chemokines with significant differences. (**B**) Other chemokines without significant changes. Representative data from three independent experiments, mean ± SEM. Statistical analyses: Student's unpaired t test (A and B). *p<0.05; **p<0.01; n.s., not significant.

Figure S3

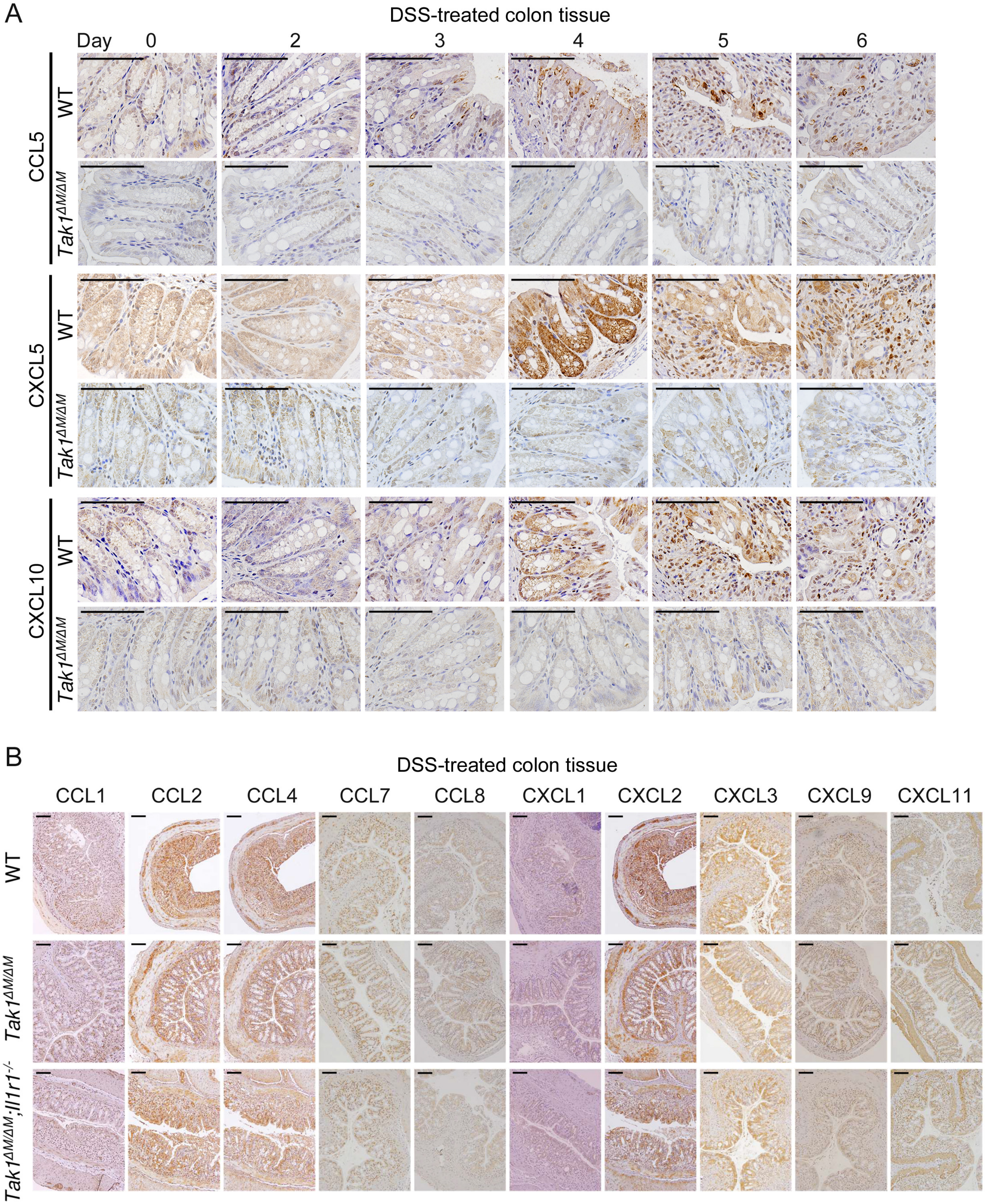
A
DSS-treated colon tissue
Day 0 2 3 4 5 6
CCL5
WT
Tak1ΔM/ΔM
CXCL5
WT
Tak1ΔM/ΔM
CXCL10
WT
Tak1ΔM/ΔM
B
DSS-treated colon tissue
CCL1 CCL2 CCL4 CCL7 CCL8 CXCL1 CXCL2 CXCL3 CXCL9 CXCL11
WT
Tak1ΔM/ΔM
Tak1ΔM/ΔM;Il1r1-/-

Fig. S3. Chemokine expression is inhibited in the colon tissues of colitis-resistant mice. (**A**) IHC staining of CCL5, CXCL5, and CXCL10 (40X) on colon sections collected on different time points from WT and *Tak1*$^{\Delta M/\Delta M}$ mice with 5% DSS treatment for 5 days. Scale bar: 100 µm. (**B**) IHC staining of chemokines (10X) on colon sections collected on day 5 from WT, *Tak1*$^{\Delta M/\Delta M}$, and double KO mice with 5% DSS treatment for 5 days. Scale bar: 100 µm. Representative data from three independent experiments.

Figure S4

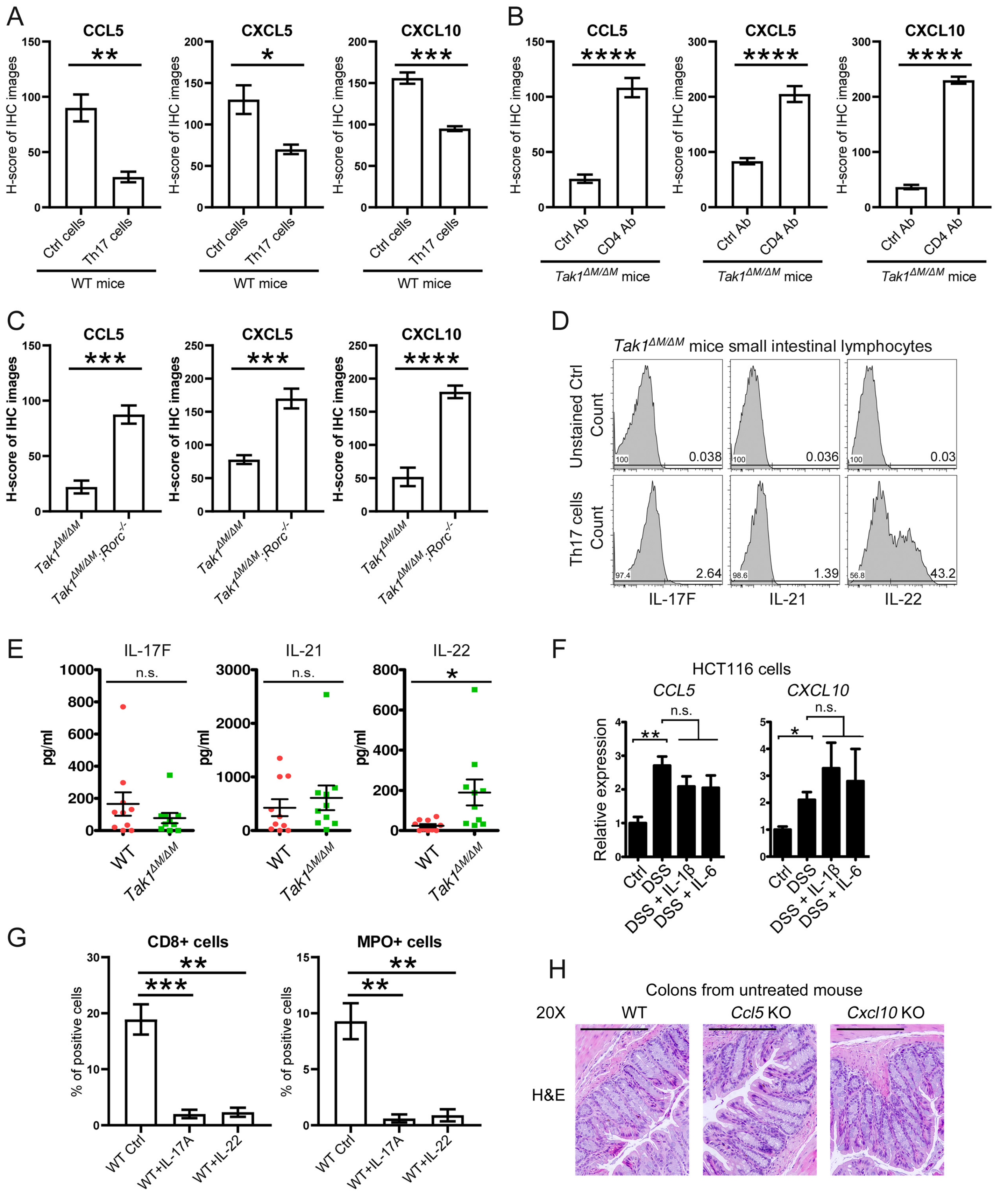

A
CCL5
CXCL5
CXCL10
H-score of IHC images
Ctrl cells
Th17 cells
WT mice
B
CD4 Ab
Ctrl Ab
Tak1ΔM/ΔM mice
C
Tak1ΔM/ΔM
Tak1ΔM/ΔM;Rorc-/-
D
Tak1ΔM/ΔM mice small intestinal lymphocytes
Unstained Ctrl Count
Th17 cells Count
IL-17F
IL-21
IL-22
E
pg/ml
n.s.
WT
F
HCT116 cells
Relative expression
Ctrl
DSS
DSS + IL-1β
DSS + IL-6
G
CD8+ cells
MPO+ cells
% of positive cells
WT Ctrl
WT+IL-17A
WT+IL-22
H
Colons from untreated mouse
20X
H&E
Ccl5 KO
Cxcl10 KO

Fig. S4. Th17 cells and their cytokines inhibit key chemokine expression in the colon after DSS treatment. (**A-C**) Quantification and statistical analyses of chemokine levels by the H-score of the IHC images in Figure 3A-C. Representative data from two independent experiments, mean ± SEM. (**D**) The lymphocyte population was gated from the FSC-A/SSC-A plot, followed by the single cell gating in FSC-A/FSC-H plot. Then the IL-17F, IL-21, and IL-22 expression were tested by flow cytometry in gated Th17 cells ($CD4^+$/IL-$17A^+$) from intestinal LP in *Tak1*$^{\Delta M/\Delta M}$ mice. Unstained cells were recorded as the control group. Representative data from three independent experiments. (**E**) IL-17F, IL-21, and IL-22 production in serum levels from untreated WT and *Tak1*$^{\Delta M/\Delta M}$ mice were tested by ELISA. Representative data from two independent experiments, mean ± SEM. (**F**) HCT116 cells were pre-treated with BSA control or different recombinant cytokines, followed by overnight 2% DSS treatment. Control buffer and cytokines were added when replacing the medium. Chemokine expression was tested by qPCR on day 4. Representative data from two independent experiments, mean ± SEM. (**G**) Statistical analyses of the positive immune cell percentages in Figure 3F. Representative data from two independent experiments, mean ± SEM. (**H**) H&E staining on colon sections from untreated WT, *Ccl5*$^{-/-}$, and *Cxcl10*$^{-/-}$ mice (20X, scale bar: 200 μm). Representative data from two independent experiments. Statistical analyses: Student's unpaired t test (A-C, E-G). *p<0.05; **p<0.01; ***p<0.001; ****p<0.0001; n.s., not significant.

Figure S5

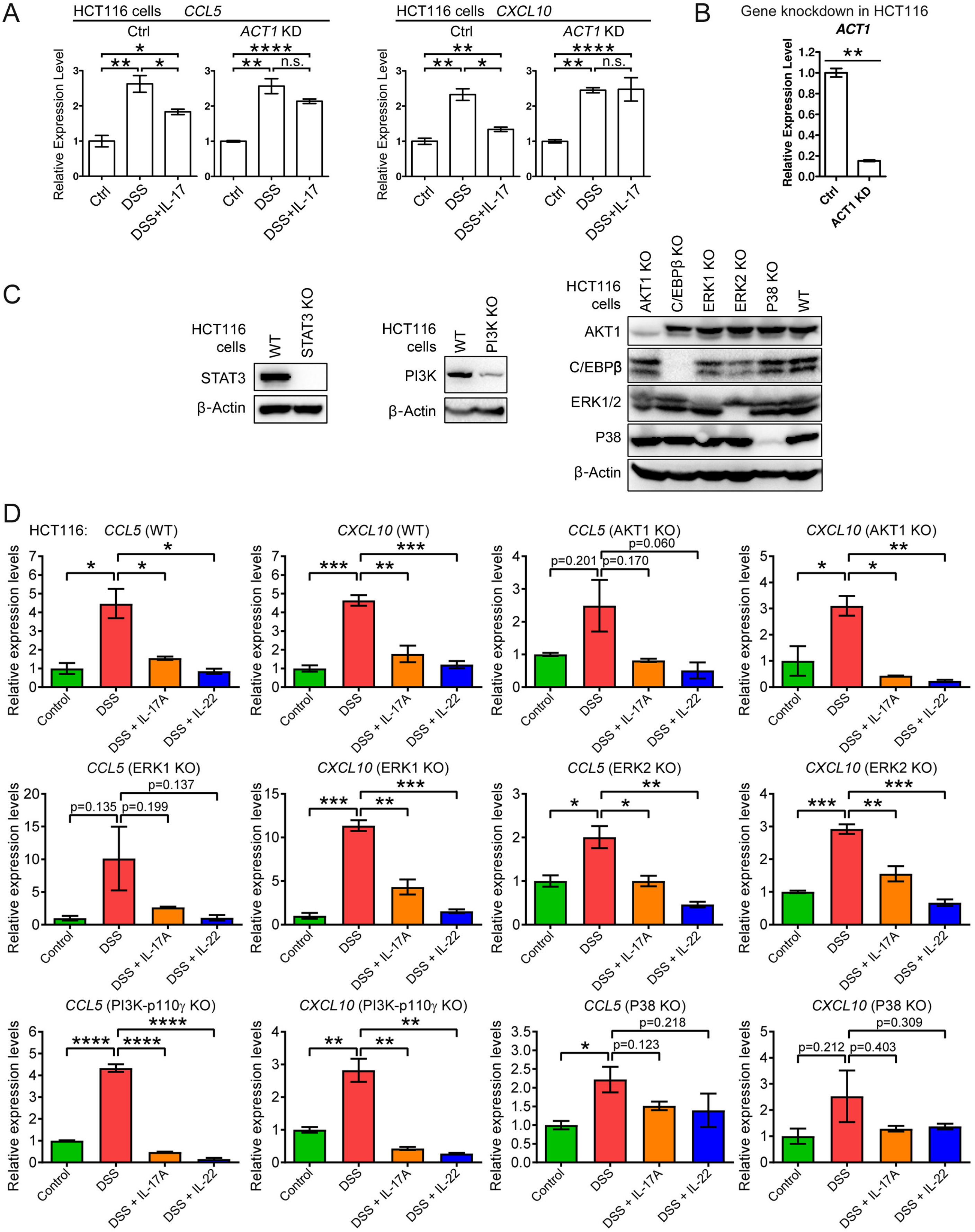

A
HCT116 cells CCL5
Ctrl
ACT1 KD
HCT116 cells CXCL10
Relative Expression Level
Ctrl
DSS
DSS+IL-17
n.s.
B
Gene knockdown in HCT116
ACT1
Relative Expression Level
Ctrl
ACT1 KD
C
HCT116 cells
WT
STAT3 KO
STAT3
β-Actin
PI3K KO
PI3K
AKT1 KO
C/EBPβ KO
ERK1 KO
ERK2 KO
P38 KO
AKT1
C/EBPβ
ERK1/2
P38
D
HCT116: CCL5 (WT)
CXCL10 (WT)
CCL5 (AKT1 KO)
CXCL10 (AKT1 KO)
p=0.201
p=0.170
p=0.060
CCL5 (ERK1 KO)
CXCL10 (ERK1 KO)
CCL5 (ERK2 KO)
CXCL10 (ERK2 KO)
p=0.135
p=0.199
p=0.137
CCL5 (PI3K-p110γ KO)
CXCL10 (PI3K-p110γ KO)
CCL5 (P38 KO)
CXCL10 (P38 KO)
p=0.123
p=0.218
p=0.212
p=0.403
p=0.309
Relative expression levels
Control
DSS
DSS + IL-17A
DSS + IL-22

Fig. S5. Th17 cytokines regulate chemokine expression through C/EBPβ and STAT3 signaling. (**A**) HCT116 cells (WT control and *ACT1* knockdown) were pre-treated with BSA control and recombinant IL-17A, followed by overnight 2% DSS treatment. Control buffer and cytokines were added when replacing the medium. Chemokine expression on day 4 was tested by qPCR. Representative data from two independent experiments, mean ± SEM. (**B**) The knockdown efficacy of *ACT1* was tested in HCT116 cells (WT control and *ACT1* knockdown) by qPCR. Representative data from two independent experiments, mean ± SEM. (**C**) Cell lysates were harvested from untreated HCT116 cells (WT and multiple KO cells). Western blotting was performed to determine the KO efficacy in these KO cells. Representative data from two independent experiments. (**D**) HCT116 cells (WT and multiple KO cells) were pre-treated with BSA control, or recombinant IL-17A or IL-22, followed by overnight 2% DSS treatment. Control buffer and cytokines were added when replacing the medium. Chemokine expression was tested by qPCR on day 4. Representative data from two independent experiments, mean ± SEM. Statistical analyses: Student's unpaired t test (A, B, and D). *$p<0.05$; **$p<0.01$; ***$p<0.001$; ****$p<0.0001$; n.s., not significant.

Figure S6

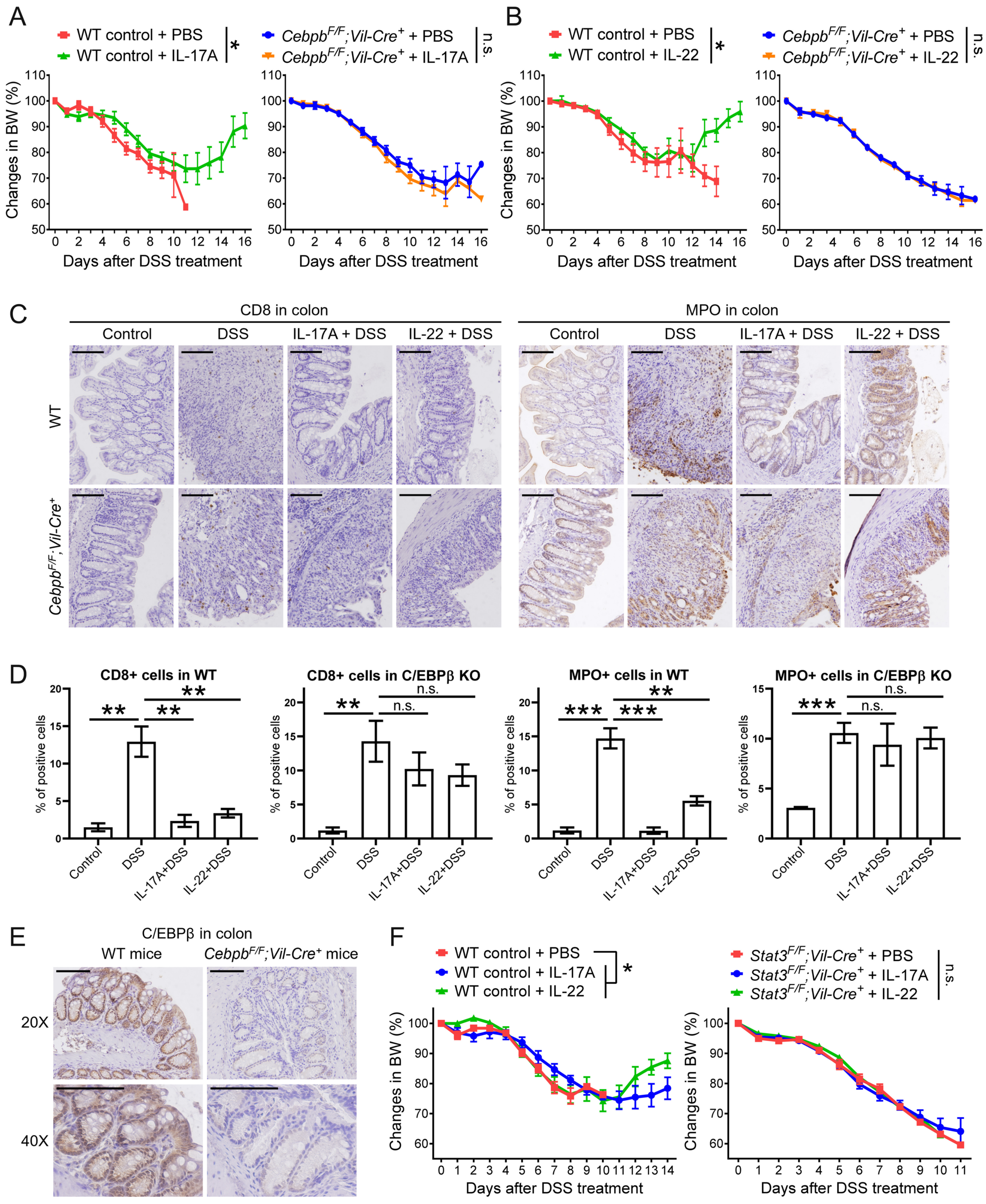

A
WT control + PBS
WT control + IL-17A
*
CebpbF/F;Vil-Cre+ + PBS
CebpbF/F;Vil-Cre+ + IL-17A
n.s.
Changes in BW (%)
Days after DSS treatment
B
WT control + PBS
WT control + IL-22
*
CebpbF/F;Vil-Cre+ + PBS
CebpbF/F;Vil-Cre+ + IL-22
n.s.
C
CD8 in colon
MPO in colon
Control
DSS
IL-17A + DSS
IL-22 + DSS
WT
CebpbF/F; Vil-Cre+
D
CD8+ cells in WT
CD8+ cells in C/EBPβ KO
MPO+ cells in WT
MPO+ cells in C/EBPβ KO
% of positive cells
Control
DSS
IL-17A+DSS
IL-22+DSS
n.s.
E
C/EBPβ in colon
WT mice
CebpbF/F;Vil-Cre+ mice
20X
40X
F
WT control + PBS
WT control + IL-17A
WT control + IL-22
*
Stat3F/F;Vil-Cre+ + PBS
Stat3F/F;Vil-Cre+ + IL-17A
Stat3F/F;Vil-Cre+ + IL-22
n.s.
Changes in BW (%)
Days after DSS treatment

Fig. S6. Th17 cytokines fail to protect C/EBPβ or STAT3 KO mice against colitis. (**A**) WT and *Cebpb*$^{F/F}$*;Vil-Cre*$^{+/+}$ mice were i.p. injected with BSA buffer as control (WT: n=12; KO: n=12) or recombinant IL-17A (WT: n=12; KO: n=13), and treated with 5% DSS for 5 days. Body weight changes were observed. Combined data from three independent experiments, mean ± SEM. (**B**) WT and *Cebpb*$^{F/F}$*;Vil-Cre*$^{+/+}$ mice were i.p. injected with BSA buffer as control or recombinant IL-22, and treated with 5% DSS for 5 days. Body weight changes were observed (n=10 for all groups). Combined data from two independent experiments, mean ± SEM. (**C-D**) IHC staining of CD8 and MPO (20X) on colon sections collected on day 7 after 2.5% DSS treatment for 5 days (scale bar: 100 μm), and the statistical analyses of positive immune cell percentages. Representative data from two independent experiments, mean ± SEM. (**E**) IHC staining of C/EBPβ on colon sections from untreated WT and *Cebpb*$^{F/F}$*;Vil-Cre*$^{+/+}$ mice (20X and 40X, scale bar: 100 μm). (**F**) WT and *Stat3*$^{F/F}$*;Vil-Cre*$^{+/+}$ mice were i.p. injected with BSA buffer as control (WT: n=8; KO: n=12), recombinant IL-17A (WT: n=8; KO: n=11), or IL-22 (WT: n=7; KO: n=12), and treated with 5% DSS for 5 days. Body weight changes were observed. Combined data from two independent experiments, mean ± SEM. Statistical analyses: ANOVA and student's unpaired t test (A, B, and F). Student's unpaired t test (D). *p<0.05; n.s., not significant.

Figure S7

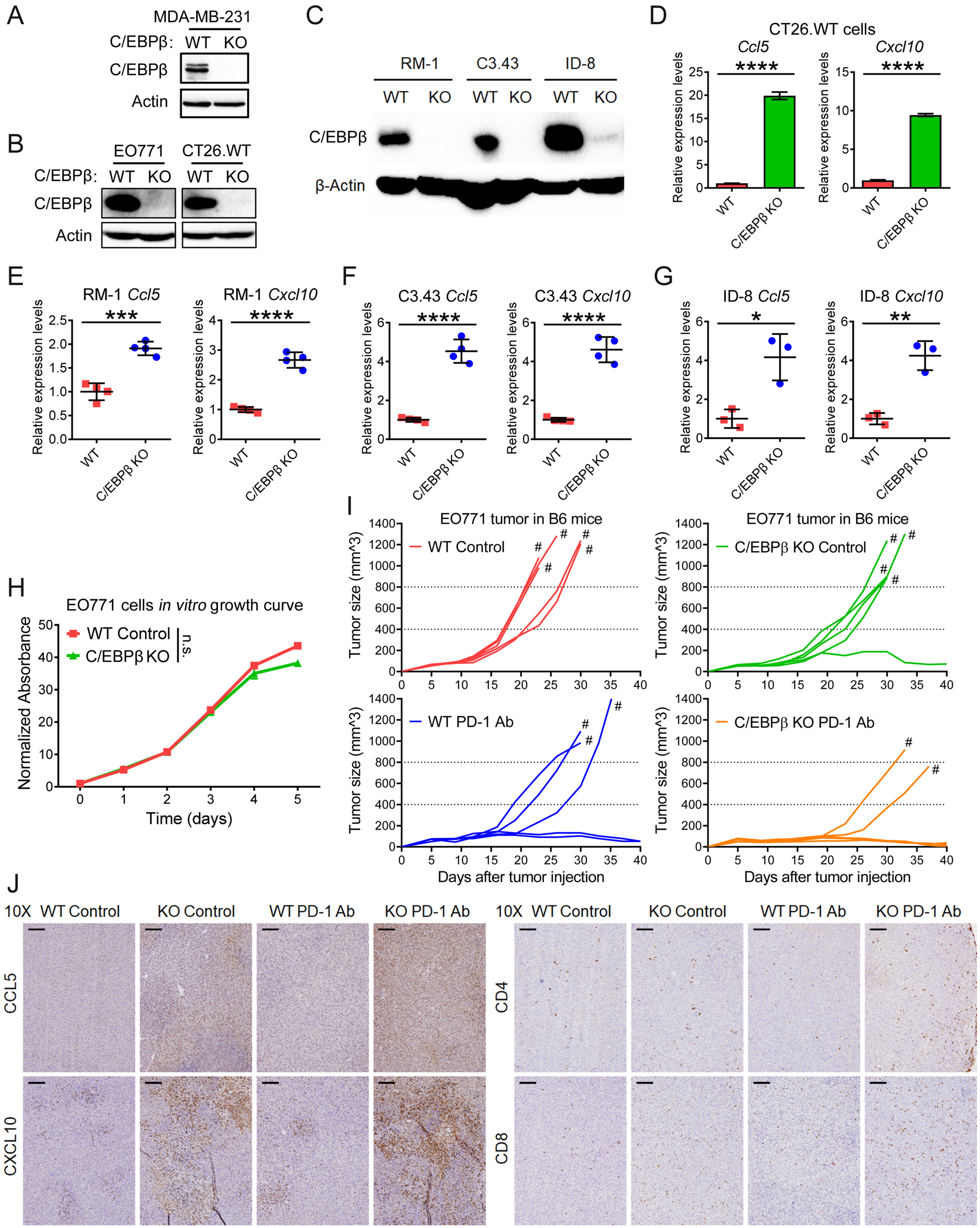

A
MDA-MB-231
C/EBPβ: WT KO
C/EBPβ
Actin
B
EO771
CT26.WT
C/EBPβ: WT KO WT KO
C/EBPβ
Actin
C
RM-1
C3.43
ID-8
WT KO WT KO WT KO
C/EBPβ
β-Actin
D
CT26.WT cells
Ccl5
Cxcl10
Relative expression levels
WT
C/EBPβ KO
E
RM-1 Ccl5
RM-1 Cxcl10
F
C3.43 Ccl5
C3.43 Cxcl10
G
ID-8 Ccl5
ID-8 Cxcl10
H
EO771 cells in vitro growth curve
WT Control
C/EBPβ KO
n.s.
Normalized Absorbance
Time (days)
I
EO771 tumor in B6 mice
WT Control
C/EBPβ KO Control
WT PD-1 Ab
C/EBPβ KO PD-1 Ab
Tumor size (mm^3)
Days after tumor injection
J
10X WT Control
KO Control
WT PD-1 Ab
KO PD-1 Ab
CCL5
CXCL10
CD4
CD8

Fig. S7. C/EBPβ KO triggers chemokine expression in cancer cells and promotes the efficacy of cancer immunotherapy. (**A**) C/EBPβ was knocked out in MDA-MB-231 human cancer cells. The KO efficacy was detected by western blotting. Representative data from two independent experiments. (**B**) C/EBPβ was knocked out in EO771 and CT26.WT cells. The KO efficacy was detected by western blotting. Representative data from two independent experiments. (**C**) C/EBPβ was knocked out in RM-1, C3.43, and ID-8 cancer cells. The KO efficacy was detected by western blotting. Representative data from two independent experiments. (**D**) C/EBPβ was knocked out in CT26.WT cells. The chemokine expression on RNA levels was detected in WT and KO cells by qPCR. Representative data from two independent experiments, mean ± SD. (**E-G**) C/EBPβ was knocked out in multiple cancer cells. The chemokine expression on RNA levels in RM-1 (**E**), C3.43 (**F**), and ID-8 (**G**) was detected in WT and KO cells by qPCR. Representative data from two independent experiments, mean ± SD. (**H**) Normalized *in vitro* growth curves of WT and C/EBPβ KO EO771 cells. Data from Fig. 7C were normalized with the value on day 0 for the relative fold changes. Representative data from two independent experiments, mean ± SEM. (**I**) WT and C/EBPβ KO EO771 cells were injected into the mammary fat pad of WT B6 mice (n=5 for all groups), with i.p. injected isotype control or PD-1 antibody twice a week since day 5. The tumor growth curve for each individual mouse from Fig. 7E was shown separately. #: Early euthanasia of tumor-bearing mice at the humane endpoint when the tumors reached 1.5 cm in diameter, per USC IACUC policy. Representative data from two independent experiments. (**J**) IHC staining of key chemokines (CCL5 and CXCL10) and tumor-infiltrated T cells (CD4 and CD8) on WT and C/EBPβ KO EO771 tumor sections from WT B6 mice with or without PD-1 antibody treatment (10X, scale bar: 100 μm). Representative data from two independent experiments. Statistical analyses: Student's unpaired t test (D, E, F, and G). ANOVA and student's unpaired t test (H). *$p<0.05$; **$p<0.01$; ***$p<0.001$; ****$p<0.0001$; n.s., not significant.

Figure S8

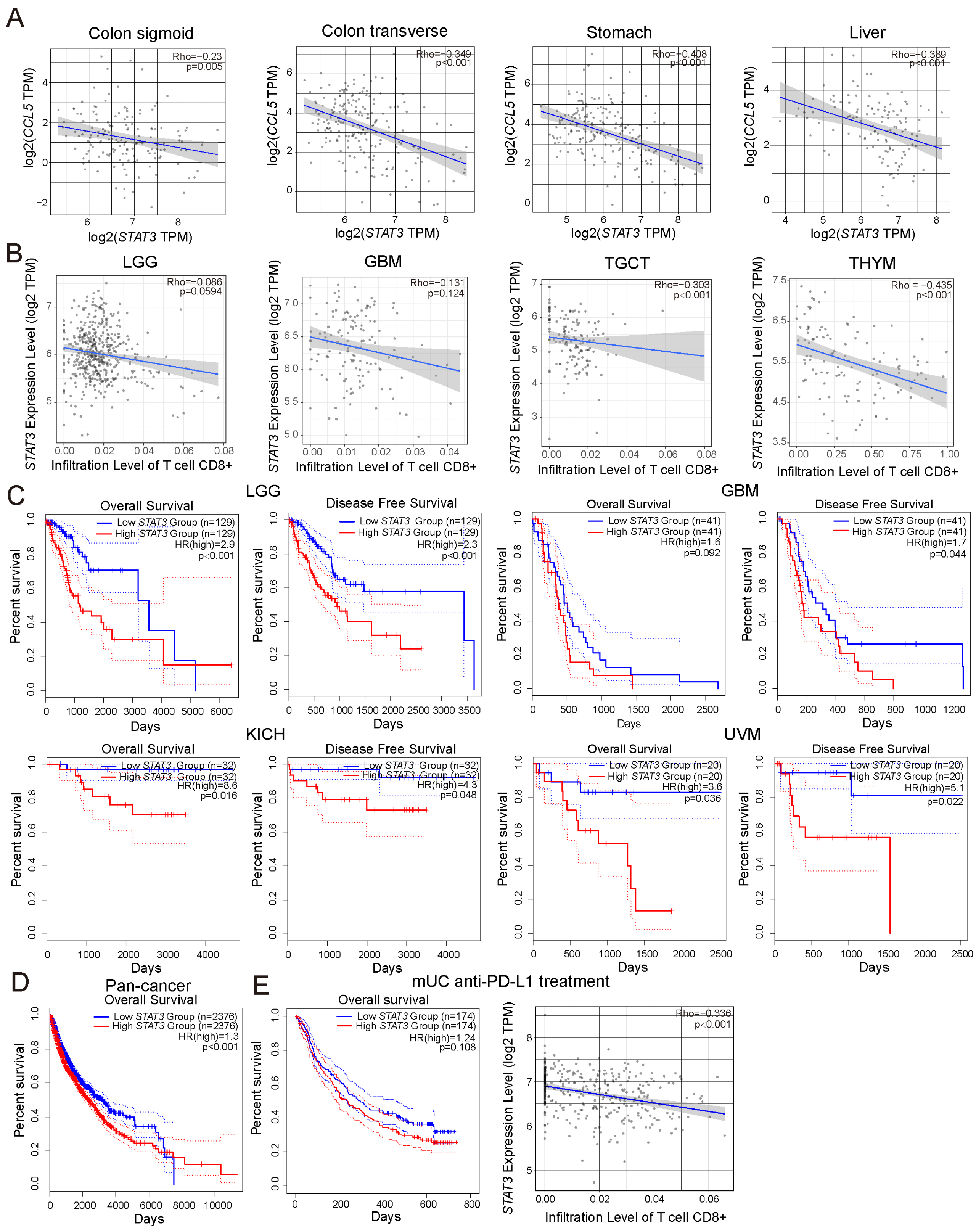
A
Colon sigmoid
Colon transverse
Stomach
Liver
log2(CCL5 TPM)
log2(STAT3 TPM)
Rho=−0.23 p=0.005
Rho=−0.349 p<0.001
Rho=−0.408 p<0.001
Rho=−0.389 p<0.001
B
LGG
GBM
TGCT
THYM
STAT3 Expression Level (log2 TPM)
Infiltration Level of T cell CD8+
Rho=−0.086 p=0.0594
Rho=−0.131 p=0.124
Rho=−0.303 p<0.001
Rho = −0.435 p<0.001
C
LGG
GBM
KICH
UVM
Overall Survival
Disease Free Survival
Percent survival
Days
Low STAT3 Group (n=129)
High STAT3 Group (n=129)
HR(high)=2.9
p<0.001
HR(high)=2.3
p<0.001
Low STAT3 Group (n=41)
High STAT3 Group (n=41)
HR(high)=1.6
p=0.092
HR(high)=1.7
p=0.044
Low STAT3 Group (n=32)
High STAT3 Group (n=32)
HR(high)=8.6
p=0.016
HR(high)=4.3
p=0.048
Low STAT3 Group (n=20)
High STAT3 Group (n=20)
HR(high)=3.6
p=0.036
HR(high)=5.1
p=0.022
D
Pan-cancer
Overall Survival
Low STAT3 Group (n=2376)
High STAT3 Group (n=2376)
HR(high)=1.3
p<0.001
E
mUC anti-PD-L1 treatment
Overall survival
Low STAT3 Group (n=174)
High STAT3 Group (n=174)
HR(high)=1.24
p=0.108
Rho=−0.336 p<0.001
STAT3 Expression Level (log2 TPM)
Infiltration Level of T cell CD8+

Fig. S8. STAT3 expression is negatively associated with CCL5 expression, $CD8^+$ T cell infiltration, and patient survival across multiple cancer types. (**A**) STAT3 expression shows a negative correlation with CCL5 across multiple human tissues. (**B**) STAT3 expression negatively correlates with $CD8^+$ T cell infiltration across multiple cancer types. (**C**) High STAT3 expression is associated with poorer overall survival and disease-free survival across multiple cancer types. (**D**) High STAT3 expression is a risk factor for overall survival in pan-cancer analysis. (**E**) STAT3 expression is negatively associated with $CD8^+$ T cell infiltration and patient survival in metastasis urothelial carcinoma patients receiving anti-PD-L1 immunotherapy. LGG: lower-grade glioma; GBM: glioblastoma multiforme; TGCT: testicular germ cell tumors; KICH: kidney chromophobe; THYM: thymoma; UVM: uveal melanoma; mUC: metastatic urothelial carcinoma.

Table S1. Genotyping primers used in this study

Mouse Strain and Sequence	Source
(*Tak1*$^{flox/flox}$) Common: 5'-GCACAGAAAATGCACAGTGCTC-3'	(*88*)
(*Tak1*$^{flox/flox}$) WT: 5'-GCTTGGGACAGGCTGGTAAAG-3'	(*88*)
(*Tak1*$^{flox/flox}$) Mutant: 5'-CTTACAAGCCGAATTCCAGCA-3'	(*88*)
(*Il1r1*) F-WT: 5'-GGTGCAACTTCATAGAGAGATGA-3'	Jackson Laboratory
(*Il1r1*) F-Mutant: 5'-CTCGTGCTTTACGGTATCGC-3'	Jackson Laboratory
(*Il1r1*) R-Common: 5'-TTCTGTGCATGCTGGAAAAC-3'	Jackson Laboratory
(*Il6*) F-Common: 5'-TTCCATCCAGTTGCCTTCTTGG-3'	Jackson Laboratory
(*Il6*) R-WT: 5'-TTCTCATTTCCACGATTTCCCAG-3'	Jackson Laboratory
(*Il6*) R-Mutant: 5'-CCGGAGAACCTGCGTGCAATCC-3'	Jackson Laboratory
(*Lyz2-Cre*) Mutant: 5'-CCCAGAAATGCCAGATTACG-3'	Jackson Laboratory
(*Lyz2-Cre*) Common: 5'-CTTGGGCTGCCAGAATTTCTC-3'	Jackson Laboratory
(*Lyz2-Cre*) WT: 5'-TTACAGTCGGCCAGGCTGAC-3'	Jackson Laboratory
(*Rorc*) Common F: 5'-CCCCCTGCCCAGAAACACT-3'	Jackson Laboratory
(*Rorc*) WT R: 5'-GGATGCCCCCATTCACTTACTTCT-3'	Jackson Laboratory
(*Rorc*) Mutant R: 5'-CGGACACGCTGAACTTGTGG-3'	Jackson Laboratory
(*Il17-GFP*) F: 5'-GACTTCAAGGAGGACGGCAACAT-3'	Lab of Scott Durum
(*Il17-GFP*) R: 5'-GAGAGACCATGATGGTCACTGGA-3'	Lab of Scott Durum
(*Il17rc*) WT F: 5'-GAGCCTGAAGAAGCTGGAAA-3'	Lab of Xiaoxia Li
(*Il17rc*) Mutant F: 5'-TCGCCTTCTTGACGAGTTCT-3'	Lab of Xiaoxia Li
(*Il17rc*) Common R: 5'-CAAGTGTTGGCAGAGATGGA-3'	Lab of Xiaoxia Li
(*Ccl5*) Common F: 5'-TTGGAAAGAAGGGGAGGTCT-3'	Jackson Laboratory
(*Ccl5*) WT R: 5'-ATGCATCTCCCACAGCCTCT-3'	Jackson Laboratory
(*Ccl5*) Mutant R: 5'-TGGATGTGGAATGTGTGCGAG-3'	Jackson Laboratory
(*Cxcl10*) WT: 5'-TGCCACGATGAAAAAGAATG-3'	Jackson Laboratory
(*Cxcl10*) Common: 5'-GGGACAGAATGGGTTTGTGT-3'	Jackson Laboratory
(*Cxcl10*) Mutant: 5'-GCCAGAGGCCACTTGTGTAG-3'	Jackson Laboratory
(*Cebpb*$^{flox/flox}$) F: 5'-GTCGCATCTGGGCTTTTCT-3'	Jackson Laboratory
(*Cebpb*$^{flox/flox}$) R: 5'-CTGACCCAGCTGTCTGGACT-3'	Jackson Laboratory
(*Stat3*$^{flox/flox}$) F: 5'-TTGACCTGTGCTCCTACAAAAA-3'	Jackson Laboratory
(*Stat3*$^{flox/flox}$) R: 5'-CCCTAGATTAGGCCAGCACA-3'	Jackson Laboratory
(*Vil-Cre*) F: 5'-TTCTCCTCTAGGCTCGTCCA-3'	Jackson Laboratory
(*Vil-Cre*) R: 5'-CATGTCCATCAGGTTCTTGC-3'	Jackson Laboratory

Table S2. Antibodies used in this study

Assay and Reagent	Source	Identifier
FC: Anti-mouse CD3	Thermo Fisher	Clone: 17A2
FC: Anti-mouse CD4	Thermo Fisher	Clone: RM4-5
FC: Anti-mouse CD8	Thermo Fisher	Clone: 53-6.7
FC: Anti-mouse IL-17A	Thermo Fisher	Clone: eBio17B7
FC: Anti-mouse IL-17F	Thermo Fisher	Clone: eBio18F10
FC: Anti-mouse IL-21	Thermo Fisher	Clone: FFA21
FC: Anti-mouse IL-22	Thermo Fisher	Clone: IL22JOP
FC: Anti-mouse CD11b	Thermo Fisher	Clone: M1/70
FC: Anti-mouse Ly-6G	Thermo Fisher	Clone: RB6-8C5
FC: Anti-mouse F4/80	Thermo Fisher	Clone: BM8
FC: Anti-mouse CD45	Thermo Fisher	Clone: 30-F11
IHC: Anti-mouse Claudin 3	Thermo Fisher	Cat#: 34-1700
IHC: Anti-mouse MPO	Abcam	Cat#: AB45977
IHC: Anti-mouse CD68	Abcam	Cat#: AB955
IHC: Anti-mouse CD4	Abcam	Cat#: AB183685
IHC: Anti-mouse CD8	Cell Signaling	Cat#: 98941
IHC: Anti-mouse CCL5	Bioss	Cat#: bs-1324R
IHC: Anti-mouse CXCL5	Bioss	Cat#: bs-2549R
IHC: Anti-mouse CXCL10	Bioss	Cat#: bs-1502R
IHC: Anti-mouse CCL1	Bioss	Cat#: bs-2450R
IHC: Anti-mouse CCL7	Bioss	Cat#: bs-1987R
IHC: Anti-mouse CCL8	Bioss	Cat#: bs-1984R
IHC: Anti-mouse CXCL1	Bioss	Cat#: bs-0863R
IHC: Anti-mouse CXCL3	Bioss	Cat#: bs-2547R
IHC: Anti-mouse CXCL9	Bioss	Cat#: bs-2551R
IHC: Anti-mouse CXCL11	Bioss	Cat#: bs-2552R
IHC: Anti-mouse Ki-67	Cell Signaling	Cat#: 12202
IHC: Anti-mouse C/EBPβ	Thermo Fisher	Cat#: PA5-27244
IHC: HRP-linked rabbit 2nd antibody	DAKO	Cat#: K4003
IHC: HRP-linked mouse 2nd antibody	DAKO	Cat#: K4001
WB: Anti-C/EBPβ for mouse cells	Cell Signaling	Cat#: 3087
WB: Anti-C/EBPβ for human cells	Thermo Fisher	Cat#: PA5-27244
WB: Anti-Phosphorylated C/EBPβ	Thermo Fisher	Cat#: PA5-17686
WB: Anti-STAT3	Cell Signaling	Cat#: 9139
WB: Anti-Phosphorylated STAT3	Cell Signaling	Cat#: 9145
WB: Anti-AKT1	Cell Signaling	Cat#: 4691
WB: Anti-ERK1/2	Cell Signaling	Cat#: 9102
WB: Anti-P38	Cell Signaling	Cat#: 9212
WB: Anti-PI3K-p110γ	Cell Signaling	Cat#: 5405
WB: Anti-β-Actin	Sigma-Aldrich	Cat#: A1978
WB: HRP-linked rabbit 2nd antibody	Thermo Fisher	Cat#: 31460
WB: HRP-linked mouse 2nd antibody	Thermo Fisher	Cat#: 31430

Table S3. Real-time qPCR primers used in this study

Target and Sequence	Source
(mouse *Ccl1*) F: 5'-CCCAGCTGTGGTATTCAGGC-3'	(*89*)
(mouse *Ccl1*) R: 5'-GTGATTTTGAACCCACGTTTTG-3'	(*89*)
(mouse *Ccl2*) F: 5'-GCTGGAGCATCCACGTGTT-3'	(*89*)
(mouse *Ccl2*) R: 5'-ATCTTGCTGGTGAATGAGTAGCA-3'	(*89*)
(mouse *Ccl3*) F: 5'-CCAAGTCTTCTCAGCGCCAT-3'	(*89*)
(mouse *Ccl3*) R: 5'-GAATCTTCCGGCTGTAGGAGAAG-3'	(*89*)
(mouse *Ccl4*) F: 5'-TCTGCGTGTCTGCCCTCTC-3'	(*89*)
(mouse *Ccl4*) R: 5'-TGCTGAGAACCCTGGAGCA-3'	(*89*)
(mouse *Ccl5*) F: 5'-GCAAGTGCTCCAATCTTGCA-3'	(*89*)
(mouse *Ccl5*) R: 5'-CTTCTCTGGGTTGGCACACA-3'	(*89*)
(mouse *Ccl6*) F: 5'-CTTGGGTCCCAGGCTGG-3'	(*89*)
(mouse *Ccl6*) R: 5'-AGTGT CTTGAAAGCCTTGATGAATT-3'	(*89*)
(mouse *Ccl7*) F: 5'-GGGAA GCTGTTATCTTCAAGACAAA-3'	(*89*)
(mouse *Ccl7*) R: 5'-CTCCTCGACCCACTTCTGATG-3'	(*89*)
(mouse *Ccl8*) F: 5'-GCTACGAGAGAATCAACAATATCCAGT-3'	(*89*)
(mouse *Ccl8*) R: 5'-CAGAGAGACATACCCTGCTTGGT-3'	(*89*)
(mouse *Ccl9*) F: 5'-GGTTCCAGGTCTGTGCCAA-3'	(*89*)
(mouse *Ccl9*) R: 5'-GTGGT TGTGAGTTTTCTCCAATCT-3'	(*89*)
(mouse *Ccl11*) F: 5'-CCAGGCTCCATCCCAACTT-3'	(*89*)
(mouse *Ccl11*) R: 5'-TGGTGATTCTTTTGTAGCTCTTCAGT-3'	(*89*)
(mouse *Ccl12*) F: 5'-AATCACAAGCAGCCAGTGTCC-3'	(*89*)
(mouse *Ccl12*) R: 5'-TCAGCACAGATCTCCTTATCCAGT-3'	(*89*)
(mouse *Ccl17*) F: 5'-GGATGCCATCGTGTTTCTGA-3'	(*89*)
(mouse *Ccl17*) R: 5'-GCCTTCTTCACATGTTTGTCTTTG-3'	(*89*)
(mouse *Ccl19*) F: 5'-ATGCGGAAGACTGCTGCC-3'	(*89*)
(mouse *Ccl19*) R: 5'-AGCGGAAGGCTTTCACGAT-3'	(*89*)
(mouse *Ccl20*) F: 5'-GTACTGCTGGCTCACCTCTG-3'	This paper
(mouse *Ccl20*) R: 5'-GCTTCATCGGCCATCTGTCT-3'	This paper
(mouse *Ccl21*) F: 5'-CCCCGGCTGCAGGAA-3'	(*89*)
(mouse *Ccl21*) R: 5'-TGTTCAGTTCTCTTGCAGCCC-3'	(*89*)
(mouse *Ccl22*) F: 5'-TGCCAGGACTACATCCGTCA-3'	(*89*)
(mouse *Ccl22*) R: 5'-GGCAGGATTTTGAGGTCCAG-3'	(*89*)
(mouse *Ccl24*) F: 5'-TGGTAGCCTGCGCGTGTT-3'	(*89*)
(mouse *Ccl24*) R: 5'-AAGGACGTGCAGCAAGATGA-3'	(*89*)
(mouse *Ccl25*) F: 5'-TGAAAGGAAGAAGTCAAACCATATGA-3'	(*89*)
(mouse *Ccl25*) R: 5'-AGGGTGGCACTCCTCACG-3'	(*89*)
(mouse *Ccl26*) F: 5'-GACAAGAGCTGCACCAGTGA-3'	This paper
(mouse *Ccl26*) R: 5'-CTGGCTGGACACAGAATTGC-3'	This paper
(mouse *Ccl27*) F: 5'-CAGGCTGCTGAGGAGGATTG-3'	(*89*)
(mouse *Ccl27*) R: 5'-CACGACAGCCTGGAGGTGA-3'	(*89*)
(mouse *Ccl28*) F: 5'-CATACTTCCCATGGCCTCC-3'	(*89*)
(mouse *Ccl28*) R: 5'-GAGAGGCTTCGTGCCTGTG-3'	(*89*)

(mouse *Cxcl1*) F: 5’-AATGAGCTGCGCTGTCAGTG-3’	(*89*)
(mouse *Cxcl1*) R: 5’-TGAGGGCAACACCTTCAAGC-3’	(*89*)
(mouse *Cxcl2*) F: 5’-CCTGCCAAGGGTTGACTTCA-3’	(*89*)
(mouse *Cxcl2*) R: 5’-TTCTGTCTGGGCGCAGTG-3’	(*89*)
(mouse *Cxcl3*) F: 5’-AAGTTTGCCTCAACCCCCAA-3’	This paper
(mouse *Cxcl3*) R: 5’-GTGAGGGGCTTCCTCCTTTC-3’	This paper
(mouse *Cxcl4*) F: 5’-GCCTGGAGGTGATCAAGGC-3’	(*89*)
(mouse *Cxcl4*) R: 5’-GGCAAATTTTCCTCCCATTCT-3’	(*89*)
(mouse *Cxcl5*) F: 5’-CATCCCCAGCGGTTCCA-3’	(*89*)
(mouse *Cxcl5*) R: 5’-CGTGAACAGCAACAGAAATGC-3’	(*89*)
(mouse *Cxcl7*) F: 5’-CGTGCCTGGACCCAAATG-3’	(*89*)
(mouse *Cxcl7*) R: 5’-GCTGGTCAGTAACCTTCCAAGATT-3’	(*89*)
(mouse *Cxcl9*) F: 5’-TGCACGATGCTCCTGCA-3’	(*89*)
(mouse *Cxcl9*) R: 5’-AGGTCTTTGAGGGATTTGTAGTGG-3’	(*89*)
(mouse *Cxcl10*) F: 5’-GACGGTCCGCTGCAACTG-3’	(*89*)
(mouse *Cxcl10*) R: 5’-GCTTCCCTATGGCCCTCATT-3’	(*89*)
(mouse *Cxcl11*) F: 5’-CGGGATGAAAGCCGTCAA-3’	(*89*)
(mouse *Cxcl11*) R: 5’-AACTTTGTCGCAGCCGTTACTC-3’	(*89*)
(mouse *Cxcl12*) F: 5’-GCTCCTCGACAGATGCCTTG-3’	(*89*)
(mouse *Cxcl12*) R: 5’-GACCCTGGCACTGAACTGGA-3’	(*89*)
(mouse *Cxcl13*) F: 5’-CATAGATCGGATTCAAGTTACGCC-3’	(*89*)
(mouse *Cxcl13*) R: 5’-TCTTGGTCCAGATCACAACTTCA-3’	(*89*)
(mouse *Cxcl14*) F: 5’-AGCACTGCCTGCACCCTAAG-3’	(*89*)
(mouse *Cxcl14*) R: 5’-TCTCGTTCCAGGCATTATACCA-3’	(*89*)
(mouse *Cxcl15*) F: 5’-GCTCCTGCTGGCTGTCCTTA-3’	(*89*)
(mouse *Cxcl15*) R: 5’-CACAGACATCGTAGCTCTTGAGTGT-3’	(*89*)
(mouse *Cxcl16*) F: 5’-AGCACACCAGCTTGGGTACC-3’	(*89*)
(mouse *Cxcl16*) R: 5’-CATGGCTGCAGTGAGGAAGA-3’	(*89*)
(mouse *Cx3cl1*) F: 5’-CAGCAGTGACCGGATCATCTC-3’	(*89*)
(mouse *Cx3cl1*) R: 5’-TGCTCTGAGGCTTAGCCGTAA-3’	(*89*)
(mouse *Gapdh*) F: 5’-AGGTCGGTGTGAACGGATTTG-3’	(*90*)
(mouse *Gapdh*) R: 5’-TGTAGACCATGTAGTTGAGGTCA-3’	(*90*)
(human *CCL5*) F: 5’-GCTGTCATCCTCATTGCTACTG-3’	(*91*)
(human *CCL5*) R: 5’-TGGTG TAGAAATACTCCTTGATGTG-3’	(*91*)
(human *CXCL5*) F: 5’-TGGACGGTGGAAACAAGG-3’	(*92*)
(human *CXCL5*) R: 5’-CTTCCCTGGGTTCAGAGAC-3’	(*92*)
(human *CXCL10*) F: 5’-GAACTGTACGCTGTACCTGCA-3’	(*93*)
(human *CXCL10*) R: 5’-TTGATGGCCTTCGATTCTGGA-3’	(*93*)
(human *ACT1*) F: 5’-GCGCTACCTTAGGGATAAGACCGT-3’	(*94*)
(human *ACT1*) R: 5’-CAGCTGCGACTCAGCGCCTT-3’	(*94*)
(human *GAPDH*) F: 5’-TCAAGAAGGTGGTGAAGCAG-3’	(*95*)
(human *GAPDH*) R: 5’-GAGGGGAGATTCAGTGTGGT-3’	(*95*)

Table S4. sgRNAs for gene knockout in cell lines

Target and Sequence	Source	Identifier
sgRNA #1 for human C/EBPβ KO: GGCCAACTTCTACTACGAGG	Thermo Fisher	Cat#: A32174
sgRNA #2 for human C/EBPβ KO: GACCTCTTCTCCGACGACTA	Thermo Fisher	Cat#: A32174
sgRNA #3 for human C/EBPβ KO: CATCGACTTCAGCCCGTACC	Thermo Fisher	Cat#: A32174
sgRNA #4 for human C/EBPβ KO: GTAGAAGTTGGCCACTTCCA	Thermo Fisher	Cat#: A32174
sgRNA #1 for human STAT3KO: CATTCGACTCTTGCAGGAAG	Thermo Fisher	Cat#: A32169
sgRNA #2 for human STAT3KO: ACAATCCGGGCAATCTCCAT	Thermo Fisher	Cat#: A32169
sgRNA #3 for human STAT3KO: GAAACTGCCGCAGCTCCATT	Thermo Fisher	Cat#: A32169
sgRNA #4 for human STAT3KO: GAAGGCGTGATTCTTCCCAC	Thermo Fisher	Cat#: A32169
sgRNA #1 for human AKT1 KO: TCACGTTGGTCCACATCCTG	Thermo Fisher	Cat#: A32172
sgRNA #2 for human AKT1 KO: GAGCGACGTGGCTATTGTGA	Thermo Fisher	Cat#: A32172
sgRNA #3 for human AKT1 KO: GCGCCACAGAGAAGTTGTTG	Thermo Fisher	Cat#: A32172
sgRNA #4 for human AKT1 KO: GAAGGTGCGTTCGATGACAG	Thermo Fisher	Cat#: A32172
sgRNA #1 for human ERK1 KO: TCGGCATCCGAGACATTCTG	Thermo Fisher	Cat#: A32182
sgRNA #2 for human ERK1 KO: TACACGCAGTTGCAGTACAT	Thermo Fisher	Cat#: A32182
sgRNA #3 for human ERK1 KO: AGTCTTGCGCACGTGGTCAT	Thermo Fisher	Cat#: A32182
sgRNA #4 for human ERK1 KO: TTCCGCCATGAGAATGTCAT	Thermo Fisher	Cat#: A32182
sgRNA #1 for human ERK2 KO: AACCTCTCGTACATCGGCGA	Thermo Fisher	Cat#: A32182
sgRNA #2 for human ERK2 KO: GCAGTAGGTCTGGTGCTCAA	Thermo Fisher	Cat#: A32182
sgRNA #3 for human ERK2 KO: TCTTTCATTTGCTCGATGGT	Thermo Fisher	Cat#: A32182
sgRNA #4 for human ERK2 KO: TACGAGAGGTTGGTGTAGCG	Thermo Fisher	Cat#: A32182
sgRNA #1 for human PI3K-p110γ KO: TTGCATTTGCGCTGGCTGGT	Thermo Fisher	Cat#: A32167

sgRNA #2 for human PI3K-p110γ KO: CAAATGCAAGAGCCCCGAAA	Thermo Fisher	Cat#: A32167
sgRNA #3 for human PI3K-p110γ KO: TGAGAGAGGACAACTGCCGA	Thermo Fisher	Cat#: A32167
sgRNA #1 for human P38 KO: AAGTAACCGCAGTTCTCTGT	Thermo Fisher	Cat#: A32182
sgRNA #2 for human P38 KO: TGGACGTTTTTACACCTGCA	Thermo Fisher	Cat#: A32182
sgRNA #3 for human P38 KO: GACAGGTTCTGGTAACGCTC	Thermo Fisher	Cat#: A32182
sgRNA #4 for human P38 KO: CACAAAAACGGGGTTACGTG	Thermo Fisher	Cat#: A32182
TrueGuide™ Synthetic Mouse C/EBPβ sgRNAs: CRISPR62684_SGM, CRISPR62681_SGM, CRISPR62691_SGM.	Thermo Fisher	Cat#: A35533

REFERENCES AND NOTES

1. C. Dong, TH17 cells in development: An updated view of their molecular identity and genetic programming. *Nat. Rev. Immunol.* **8**, 337–348 (2008).

2. E. Esplugues, S. Huber, N. Gagliani, A. E. Hauser, T. Town, Y. Y. Wan, W. O'Connor, A. Rongvaux, N. Van Rooijen, A. M. Haberman, Y. Iwakura, V. K. Kuchroo, J. K. Kolls, J. A. Bluestone, K. C. Herold, R. A. Flavell, Control of TH17 cells occurs in the small intestine. *Nature* **475**, 514–518 (2011).

3. C. Xing, Y. Du, T. Duan, K. Nim, J. Chu, H. Y. Wang, R. F. Wang, Interaction between microbiota and immunity and its implication in colorectal cancer. *Front. Immunol.* **13**, 963819 (2022).

4. K. Ghoreschi, A. Laurence, X.-P. Yang, C. M. Tato, M. J. McGeachy, J. E. Konkel, H. L. Ramos, L. Wei, T. S. Davidson, N. Bouladoux, J. R. Grainger, Q. Chen, Y. Kanno, W. T. Watford, H.-W. Sun, G. Eberl, E. M. Shevach, Y. Belkaid, D. J. Cua, W. Chen, J. J. O'Shea, Generation of pathogenic TH17 cells in the absence of TGF-β signalling. *Nature* **467**, 967–971 (2010).

5. J.-Y. Lee, J. A. Hall, L. Kroehling, L. Wu, T. Najar, H. H. Nguyen, W.-Y. Lin, S. T. Yeung, H. M. Silva, D. Li, A. Hine, P. Loke, D. Hudesman, J. C. Martin, E. Kenigsberg, M. Merad, K. M. Khanna, D. R. Littman, Serum amyloid a proteins induce pathogenic Th17 cells and promote inflammatory disease. *Cell* **180**, 79–91.e16 (2020).

6. W.-J. Chae, T. F. Gibson, D. Zelterman, L. Hao, O. Henegariu, A. L. M. Bothwell, Ablation of IL-17A abrogates progression of spontaneous intestinal tumorigenesis. *Proc. Natl. Acad. Sci. U.S.A.* **107**, 5540–5544 (2010).

7. S. I. Grivennikov, K. Wang, D. Mucida, C. A. Stewart, B. Schnabl, D. Jauch, K. Taniguchi, G. Y. Yu, C. H. Osterreicher, K. E. Hung, C. Datz, Y. Feng, E. R. Fearon, M. Oukka, L. Tessarollo, V. Coppola, F. Yarovinsky, H. Cheroutre, L. Eckmann, G. Trinchieri, M. Karin, Adenoma-linked barrier defects and microbial products drive IL-23/IL-17-mediated tumour growth. *Nature* **491**, 254–258 (2012).

8. K. Wang, M. K. Kim, G. Di Caro, J. Wong, S. Shalapour, J. Wan, W. Zhang, Z. Zhong, E. Sanchez-Lopez, L. W. Wu, K. Taniguchi, Y. Feng, E. Fearon, S. I. Grivennikov, M. Karin, Interleukin-17 receptor a signaling in transformed enterocytes promotes early colorectal tumorigenesis. *Immunity* **41**, 1052–1063 (2014).

9. W. O'Connor Jr., M. Kamanaka, C. J. Booth, T. Town, S. Nakae, Y. Iwakura, J. K. Kolls, R. A. Flavell, A protective function for interleukin 17A in T cell-mediated intestinal inflammation. *Nat. Immunol.* **10**, 603–609 (2009).

10. A. Ogawa, A. Andoh, Y. Araki, T. Bamba, Y. Fujiyama, Neutralization of interleukin-17 aggravates dextran sulfate sodium-induced colitis in mice. *Clin. Immunol.* **110**, 55–62 (2004).

11. X. O. Yang, S. H. Chang, H. Park, R. Nurieva, B. Shah, L. Acero, Y. H. Wang, K. S. Schluns, R. R. Broaddus, Z. Zhu, C. Dong, Regulation of inflammatory responses by IL-17F. *J. Exp. Med.* **205**, 1063–1075 (2008).

12. E. Limagne, R. Euvrard, M. Thibaudin, C. Rebe, V. Derangere, A. Chevriaux, R. Boidot, F. Vegran, N. Bonnefoy, J. Vincent, L. Bengrine-Lefevre, S. Ladoire, D. Delmas, L. Apetoh, F. Ghiringhelli, Accumulation of MDSC and Th17 cells in patients with metastatic colorectal cancer predicts the efficacy of a FOLFOX-bevacizumab drug treatment regimen. *Cancer Res.* **76**, 5241–5252 (2016).

13. M. Tosolini, A. Kirilovsky, B. Mlecnik, T. Fredriksen, S. Mauger, G. Bindea, A. Berger, P. Bruneval, W. H. Fridman, F. Pages, J. Galon, Clinical impact of different classes of infiltrating T cytotoxic and helper cells (Th1, Th2, Treg, Th17) in patients with colorectal cancer. *Cancer Res.* **71**, 1263–1271 (2011).

14. J. Y. Lee, E. H. Seo, C. S. Oh, J. H. Paik, D. Y. Hwang, S. H. Lee, S. H. Kim, Impact of circulating T helper 1 and 17 cells in the blood on regional lymph node invasion in colorectal cancer. *J. Cancer* **8**, 1249–1254 (2017).

15. F. Amicarella, M. G. Muraro, C. Hirt, E. Cremonesi, E. Padovan, V. Mele, V. Governa, J. Han, X. Huber, R. A. Droeser, M. Zuber, M. Adamina, M. Bolli, R. Rosso, A. Lugli, I. Zlobec, L.

Terracciano, L. Tornillo, P. Zajac, S. Eppenberger-Castori, F. Trapani, D. Oertli, G. Iezzi, Dual role of tumour-infiltrating T helper 17 cells in human colorectal cancer. *Gut* **66**, 692–704 (2017).

16. C. Xing, M. Wang, A. A. Ajibade, P. Tan, C. Fu, L. Chen, M. Zhu, Z. Z. Hao, J. Chu, X. Yu, B. Yin, J. Zhu, W. J. Shen, T. Duan, H. Y. Wang, R. F. Wang, Microbiota regulate innate immune signaling and protective immunity against cancer. *Cell Host Microbe* **29**, 959–974.e7 (2021).

17. C. G. Hurtado, F. Wan, F. Housseau, C. L. Sears, Roles for interleukin 17 and adaptive immunity in pathogenesis of colorectal cancer. *Gastroenterology* **155**, 1706–1715 (2018).

18. S. Wu, K. J. Rhee, E. Albesiano, S. Rabizadeh, X. Wu, H. R. Yen, D. L. Huso, F. L. Brancati, E. Wick, F. McAllister, F. Housseau, D. M. Pardoll, C. L. Sears, A human colonic commensal promotes colon tumorigenesis via activation of T helper type 17 T cell responses. *Nat. Med.* **15**, 1016–1022 (2009).

19. X. Song, D. Dai, X. He, S. Zhu, Y. Yao, H. Gao, J. Wang, F. Qu, J. Qiu, H. Wang, X. Li, N. Shen, Y. Qian, Growth factor FGF2 cooperates with interleukin-17 to repair intestinal epithelial damage. *Immunity* **43**, 488–501 (2015).

20. J. S. Lee, C. M. Tato, B. Joyce-Shaikh, F. Gulan, C. Cayatte, Y. Chen, W. M. Blumenschein, M. Judo, G. Ayanoglu, T. K. McClanahan, X. Li, D. J. Cua, Interleukin-23-independent IL-17 production regulates intestinal epithelial permeability. *Immunity* **43**, 727–738 (2015).

21. J. R. Maxwell, Y. Zhang, W. A. Brown, C. L. Smith, F. R. Byrne, M. Fiorino, E. Stevens, J. Bigler, J. A. Davis, J. B. Rottman, A. L. Budelsky, A. Symons, J. E. Towne, Differential roles for interleukin-23 and interleukin-17 in intestinal immunoregulation. *Immunity* **43**, 739–750 (2015).

22. S. Rutz, X. Wang, W. Ouyang, The IL-20 subfamily of cytokines—From host defence to tissue homeostasis. *Nat. Rev. Immunol.* **14**, 783–795 (2014).

23. S. Huber, N. Gagliani, L. A. Zenewicz, F. J. Huber, L. Bosurgi, B. Hu, M. Hedl, W. Zhang, W. O'Connor Jr., A. J. Murphy, D. M. Valenzuela, G. D. Yancopoulos, C. J. Booth, J. H. Cho, W.

Ouyang, C. Abraham, R. A. Flavell, IL-22BP is regulated by the inflammasome and modulates tumorigenesis in the intestine. *Nature* **491**, 259–263 (2012).

24. K. Sugimoto, A. Ogawa, E. Mizoguchi, Y. Shimomura, A. Andoh, A. K. Bhan, R. S. Blumberg, R. J. Xavier, A. Mizoguchi, IL-22 ameliorates intestinal inflammation in a mouse model of ulcerative colitis. *J. Clin. Invest.* **118**, 534–544 (2008).

25. L. A. Zenewicz, G. D. Yancopoulos, D. M. Valenzuela, A. J. Murphy, S. Stevens, R. A. Flavell, Innate and adaptive interleukin-22 protects mice from inflammatory bowel disease. *Immunity* **29**, 947–957 (2008).

26. Z. Deng, S. Wang, C. Wu, C. Wang, IL-17 inhibitor-associated inflammatory bowel disease: A study based on literature and database analysis. *Front. Pharmacol.* **14**, 1124628 (2023).

27. D. Wu, P. Wu, Q. Huang, Y. Liu, J. Ye, J. Huang, Interleukin-17: A promoter in colorectal cancer progression. *J. Immunol. Res.* **2013**, 436307 (2013).

28. B. C. Lo, S. B. Shin, D. Canals Hernaez, I. Refaeli, H. B. Yu, V. Goebeler, A. Cait, W. W. Mohn, B. A. Vallance, K. M. McNagny, IL-22 preserves gut epithelial integrity and promotes disease remission during chronic *Salmonella* infection. *J. Immunol.* **202**, 956–965 (2019).

29. P. Pavlidis, A. Tsakmaki, E. Pantazi, K. Li, D. Cozzetto, J. Digby- Bell, F. Yang, J. W. Lo, E. Alberts, A. C. C. Sa, U. Niazi, J. Friedman, A. K. Long, Y. Ding, C. D. Carey, C. Lamb, M. Saqi, M. Madgwick, L. Gul, A. Treveil, T. Korcsmaros, T. T. Macdonald, G. M. Lord, G. Bewick, N. Powell, Interleukin-22 regulates neutrophil recruitment in ulcerative colitis and is associated with resistance to ustekinumab therapy. *Nat. Commun.* **13**, 5820 (2022).

30. R. F. Wang, H. Y. Wang, Immune targets and neoantigens for cancer immunotherapy and precision medicine. *Cell Res.* **27**, 11–37 (2017).

31. J. Galon, D. Bruni, Approaches to treat immune hot, altered and cold tumours with combination immunotherapies. *Nat. Rev. Drug Discov.* **18**, 197–218 (2019).

32. L. Guo, C. Wang, X. Qiu, X. Pu, P. Chang, Colorectal cancer immune infiltrates: Significance in patient prognosis and immunotherapeutic efficacy. *Front. Immunol.* **11**, 1052 (2020).

33. K. Kohli, V. G. Pillarisetty, T. S. Kim, Key chemokines direct migration of immune cells in solid tumors. *Cancer Gene Ther.* **29**, 10–21 (2022).

34. A. J. Ozga, M. T. Chow, A. D. Luster, Chemokines and the immune response to cancer. *Immunity* **54**, 859–874 (2021).

35. A. A. Ajibade, H. Y. Wang, R. F. Wang, Cell type-specific function of TAK1 in innate immune signaling. *Trends Immunol.* **34**, 307–316 (2013).

36. Y. Y. Wan, H. Chi, M. Xie, M. D. Schneider, R. A. Flavell, The kinase TAK1 integrates antigen and cytokine receptor signaling for T cell development, survival and function. *Nat. Immunol.* **7**, 851–858 (2006).

37. A. A. Ajibade, Q. Wang, J. Cui, J. Zou, X. Xia, M. Wang, Y. Tong, W. Hui, D. Liu, B. Su, H. Y. Wang, R. F. Wang, TAK1 negatively regulates NF-κB and p38 MAP kinase activation in Gr-1^{+}CD11b^{+} neutrophils. *Immunity* **36**, 43–54 (2012).

38. S. L. Gaffen, Structure and signalling in the IL-17 receptor family. *Nat. Rev. Immunol.* **9**, 556–567 (2009).

39. M. R. Simpson-Abelson, G. Hernandez-Mir, E. E. Childs, J. A. Cruz, A. C. Poholek, A. Chattopadhyay, S. L. Gaffen, M. J. McGeachy, CCAAT/enhancer-binding protein β promotes pathogenesis of EAE. *Cytokine* **92**, 24–32 (2017).

40. L. Zhou, J. E. Lopes, M. M. W. Chong, I. I. Ivanov, R. Min, G. D. Victora, Y. Shen, J. Du, Y. P. Rubtsov, A. Y. Rudensky, S. F. Ziegler, D. R. Littman, TGF-β-induced Foxp3 inhibits TH17 cell differentiation by antagonizing RORγt function. *Nature* **453**, 236–240 (2008).

41. R. Sabat, W. Ouyang, K. Wolk, Therapeutic opportunities of the IL-22-IL-22R1 system. *Nat. Rev. Drug Discov.* **13**, 21–38 (2014).

42. M. Niehof, K. Streetz, T. Rakemann, S. C. Bischoff, M. P. Manns, F. Horn, C. Trautwein, Interleukin-6-induced tethering of STAT3 to the LAP/C/EBPbeta promoter suggests a new mechanism of transcriptional regulation by STAT3. *J. Biol. Chem.* **276**, 9016–9027 (2001).

43. S. Wirtz, C. Neufert, B. Weigmann, M. F. Neurath, Chemically induced mouse models of intestinal inflammation. *Nat. Protoc.* **2**, 541–546 (2007).

44. J. Terzic, S. Grivennikov, E. Karin, M. Karin, Inflammation and colon cancer. *Gastroenterology* **138**, 2101–2114.e5 (2010).

45. J. W. Griffith, C. L. Sokol, A. D. Luster, Chemokines and chemokine receptors: Positioning cells for host defense and immunity. *Annu. Rev. Immunol.* **32**, 659–702 (2014).

46. E. M. Disteldorf, C. F. Krebs, H. J. Paust, J. E. Turner, G. Nouailles, A. Tittel, C. Meyer-Schwesinger, G. Stege, S. Brix, J. Velden, T. Wiech, U. Helmchen, O. M. Steinmetz, A. Peters, S. B. Bennstein, A. Kaffke, C. Llanto, S. A. Lira, H. W. Mittrucker, R. A. Stahl, C. Kurts, S. H. Kaufmann, U. Panzer, CXCL5 drives neutrophil recruitment in T_H17-mediated GN. *J. Am. Soc. Nephrol.* **26**, 55–66 (2015).

47. A. Crawford, J. M. Angelosanto, K. L. Nadwodny, S. D. Blackburn, E. J. Wherry, A role for the chemokine RANTES in regulating CD8 T cell responses during chronic viral infection. *PLOS Pathog* **7**, e1002098 (2011).

48. H. D. Hickman, G. V. Reynoso, B. F. Ngudiankama, S. S. Cush, J. Gibbs, J. R. Bennink, J. W. Yewdell, CXCR3 chemokine receptor enables local $CD8^+$ T cell migration for the destruction of virus-infected cells. *Immunity* **42**, 524–537 (2015).

49. J. W. Lee, P. Wang, M. G. Kattah, S. Youssef, L. Steinman, K. DeFea, D. S. Straus, Differential regulation of chemokines by IL-17 in colonic epithelial cells. *J. Immunol.* **181**, 6536–6545 (2008).

50. X. Guo, X. Jiang, Y. Xiao, T. Zhou, Y. Guo, R. Wang, Z. Zhao, H. Xiao, C. Hou, L. Ma, Y. Lin, X. Lang, J. Feng, G. Chen, B. Shen, G. Han, Y. Li, IL-17A signaling in colonic epithelial

cells inhibits pro-inflammatory cytokine production by enhancing the activity of ERK and PI3K. *PLOS ONE* **9**, e89714 (2014).

51. S. Eyerich, K. Eyerich, A. Cavani, C. Schmidt-Weber, IL-17 and IL-22: Siblings, not twins. *Trends Immunol.* **31**, 354–361 (2010).

52. H. Zhang, H. Nguyen-Jackson, A. D. Panopoulos, H. S. Li, P. J. Murray, S. S. Watowich, STAT3 controls myeloid progenitor growth during emergency granulopoiesis. *Blood* **116**, 2462–2471 (2010).

53. L. L. Lee, S. J. Kim, Y. I. Hahn, J. H. Jang, S. Saeidi, Y. J. Surh, Stabilization of C/EBPβ through direct interaction with STAT3 in H-*Ras* transformed human mammary epithelial cells. *Biochem. Biophys. Res. Commun.* **546**, 130–137 (2021).

54. A. Swoboda, R. Nanda, "Immune checkpoint blockade for breast cancer," in *Optimizing Breast Cancer Management*, W. J. Gradishar, Ed. (Springer International Publishing, 2018), pp. 155–165.

55. C. Kasikara, V. Davra, D. Calianese, K. Geng, T. E. Spires, M. Quigley, M. Wichroski, G. Sriram, L. Suarez-Lopez, M. B. Yaffe, S. V. Kotenko, M. S. De Lorenzo, R. B. Birge, Pan-TAM tyrosine kinase inhibitor BMS-777607 enhances anti-PD-1 mAb efficacy in a murine model of triple-negative breast cancer. *Cancer Res.* **79**, 2669–2683 (2019).

56. P. G. Andres, P. L. Beck, E. Mizoguchi, A. Mizoguchi, A. K. Bhan, T. Dawson, W. A. Kuziel, N. Maeda, R. P. MacDermott, D. K. Podolsky, H. C. Reinecker, Mice with a selective deletion of the CC chemokine receptors 5 or 2 are protected from dextran sodium sulfate-mediated colitis: Lack of CC chemokine receptor 5 expression results in a NK1.1$^+$ lymphocyte-associated Th2-type immune response in the intestine. *J. Immunol.* **164**, 6303–6312 (2000).

57. H. Katoh, D. Wang, T. Daikoku, H. Sun, S. K. Dey, R. N. Dubois, CXCR2-expressing myeloid-derived suppressor cells are essential to promote colitis-associated tumorigenesis. *Cancer Cell* **24**, 631–644 (2013).

58. B. K. Popivanova, F. I. Kostadinova, K. Furuichi, M. M. Shamekh, T. Kondo, T. Wada, K. Egashira, N. Mukaida, Blockade of a chemokine, CCL2, reduces chronic colitis-associated carcinogenesis in mice. *Cancer Res.* **69**, 7884–7892 (2009).

59. S. El Sayed, I. Patik, N. S. Redhu, J. N. Glickman, K. Karagiannis, E. S. Y. El Naenaeey, G. A. Elmowalid, A. M. Abd El Wahab, S. B. Snapper, B. H. Horwitz, CCR2 promotes monocyte recruitment and intestinal inflammation in mice lacking the interleukin-10 receptor. *Sci. Rep.* **12**, 452 (2022).

60. J. Deng, R. Jiang, E. Meng, H. Wu, CXCL5: A coachman to drive cancer progression. *Front. Oncol.* **12**, 944494 (2022).

61. E. Elinav, T. Strowig, A. L. Kau, J. Henao-Mejia, C. A. Thaiss, C. J. Booth, D. R. Peaper, J. Bertin, S. C. Eisenbarth, J. I. Gordon, R. A. Flavell, NLRP6 inflammasome regulates colonic microbial ecology and risk for colitis. *Cell* **145**, 745–757 (2011).

62. D. Demon, A. Kuchmiy, A. Fossoul, Q. Zhu, T. D. Kanneganti, M. Lamkanfi, Caspase-11 is expressed in the colonic mucosa and protects against dextran sodium sulfate-induced colitis. *Mucosal Immunol.* **7**, 1480–1491 (2014).

63. P. R. Burkett, V. K. Kuchroo, IL-17 blockade in psoriasis. *Cell* **167**, 1669 (2016).

64. M. Sanford, K. McKeage, Secukinumab: First global approval. *Drugs* **75**, 329–338 (2015).

65. Y. Liu, J. Mei, L. Gonzales, G. Yang, N. Dai, P. Wang, P. Zhang, M. Favara, K. C. Malcolm, S. Guttentag, G. S. Worthen, IL-17A and TNF-α exert synergistic effects on expression of CXCL5 by alveolar type II cells in vivo and in vitro. *J. Immunol.* **186**, 3197–3205 (2011).

66. Y. Ge, M. Huang, Y.-M. Yao, Biology of interleukin-17 and its pathophysiological significance in sepsis. *Front. Immunol.* **11**, 1558 (2020).

67. W. Hueber, B. E. Sands, S. Lewitzky, M. Vandemeulebroecke, W. Reinisch, P. D. Higgins, J. Wehkamp, B. G. Feagan, M. D. Yao, M. Karczewski, J. Karczewski, N. Pezous, S. Bek, G. Bruin, B. Mellgard, C. Berger, M. Londei, A. P. Bertolino, G. Tougas, S. P. Travis,

Secukinumab in Crohn's Disease Study Group, Secukinumab, a human anti-IL-17A monoclonal antibody, for moderate to severe Crohn’s disease: Unexpected results of a randomised, double-blind placebo-controlled trial. *Gut* **61**, 1693–1700 (2012).

68. J. A. Dudakov, A. M. Hanash, M. R. M. van den Brink, Interleukin-22: Immunobiology and pathology. *Annu. Rev. Immunol.* **33**, 747–785 (2015).

69. C. Rodriguez, C. L. Araujo Furlan, J. Tosello Boari, S. N. Bossio, S. Boccardo, L. Fozzatti, F. P. Canale, C. G. Beccaria, N. G. Nuñez, D. G. Ceschin, E. Piaggio, A. Gruppi, C. L. Montes, E. V. Acosta Rodríguez, Interleukin-17 signaling influences $CD8^+$ T cell immunity and tumor progression according to the IL-17 receptor subunit expression pattern in cancer cells. *Oncoimmunology* **12**, 2261326 (2023).

70. Y. Qian, C. Liu, J. Hartupee, C. Z. Altuntas, M. F. Gulen, D. Jane-Wit, J. Xiao, Y. Lu, N. Giltiay, J. Liu, T. Kordula, Q. W. Zhang, B. Vallance, S. Swaidani, M. Aronica, V. K. Tuohy, T. Hamilton, X. Li, The adaptor Act1 is required for interleukin 17-dependent signaling associated with autoimmune and inflammatory disease. *Nat. Immunol.* **8**, 247–256 (2007).

71. Y. Ren, J. Song, X. Li, N. Luo, Rationale and clinical research progress on PD-1/PD-L1-based immunotherapy for metastatic triple-negative breast cancer. *Int. J. Mol. Sci.* **23**, 8878 (2022).

72. J. Hsu, J. J. Hodgins, M. Marathe, C. J. Nicolai, M.-C. Bourgeois-Daigneault, T. N. Trevino, C. S. Azimi, A. K. Scheer, H. E. Randolph, T. W. Thompson, L. Zhang, A. Iannello, N. Mathur, K. E. Jardine, G. A. Kirn, J. C. Bell, M. W. McBurney, D. H. Raulet, M. Ardolino, Contribution of NK cells to immunotherapy mediated by PD-1/PD-L1 blockade. *J. Clin. Invest.* **128**, 4654–4668 (2018).

73. D. Lu, Z. Ni, X. Liu, S. Feng, X. Dong, X. Shi, J. Zhai, S. Mai, J. Jiang, Z. Wang, H. Wu, K. Cai, Beyond T cells: Understanding the role of PD-1/PD-L1 in tumor-associated macrophages. *J. Immunol. Res.* **2019**, 1919082 (2019).

74. J. A. Marin-Acevedo, E. O. Kimbrough, Y. Lou, Next generation of immune checkpoint inhibitors and beyond. *J. Hematol. Oncol.* **14**, 45 (2021).

75. S. Spranger, R. Bao, T. F. Gajewski, Melanoma-intrinsic β-catenin signalling prevents anti-tumour immunity. *Nature* **523**, 231–235 (2015).

76. W. Peng, J. Q. Chen, C. Liu, S. Malu, C. Creasy, M. T. Tetzlaff, C. Xu, J. A. McKenzie, C. Zhang, X. Liang, L. J. Williams, W. Deng, G. Chen, R. Mbofung, A. J. Lazar, C. A. Torres-Cabala, Z. A. Cooper, P. L. Chen, T. N. Tieu, S. Spranger, X. Yu, C. Bernatchez, M. A. Forget, C. Haymaker, R. Amaria, J. L. McQuade, I. C. Glitza, T. Cascone, H. S. Li, L. N. Kwong, T. P. Heffernan, J. Hu, R. L. Bassett Jr., M. W. Bosenberg, S. E. Woodman, W. W. Overwijk, G. Lizee, J. Roszik, T. F. Gajewski, J. A. Wargo, J. E. Gershenwald, L. Radvanyi, M. A. Davies, P. Hwu, Loss of PTEN promotes resistance to T cell-mediated immunotherapy. *Cancer Discov.* **6**, 202–216 (2016).

77. S. Mariathasan, S. J. Turley, D. Nickles, A. Castiglioni, K. Yuen, Y. Wang, E. E. Kadel III, H. Koeppen, J. L. Astarita, R. Cubas, S. Jhunjhunwala, R. Banchereau, Y. Yang, Y. Guan, C. Chalouni, J. Ziai, Y. Senbabaoglu, S. Santoro, D. Sheinson, J. Hung, J. M. Giltnane, A. A. Pierce, K. Mesh, S. Lianoglou, J. Riegler, R. A. D. Carano, P. Eriksson, M. Hoglund, L. Somarriba, D. L. Halligan, M. S. van der Heijden, Y. Loriot, J. E. Rosenberg, L. Fong, I. Mellman, D. S. Chen, M. Green, C. Derleth, G. D. Fine, P. S. Hegde, R. Bourgon, T. Powles, TGFβ attenuates tumour response to PD-L1 blockade by contributing to exclusion of T cells. *Nature* **554**, 544–548 (2018).

78. X. Wang, C. Tokheim, S. S. Gu, B. Wang, Q. Tang, Y. Li, N. Traugh, Z. Zeng, Y. Zhang, Z. Li, B. Zhang, J. Fu, T. Xiao, W. Li, C. A. Meyer, J. Chu, P. Jiang, P. Cejas, K. Lim, H. Long, M. Brown, X. S. Liu, In vivo CRISPR screens identify the E3 ligase Cop1 as a modulator of macrophage infiltration and cancer immunotherapy target. *Cell* **184**, 5357–5374.e22 (2021).

79. L. M. Jones, M. L. Broz, J. J. Ranger, J. Ozcelik, R. Ahn, D. Zuo, J. Ursini-Siegel, M. T. Hallett, M. Krummel, W. J. Muller, STAT3 establishes an immunosuppressive microenvironment during the early stages of breast carcinogenesis to promote tumor growth and metastasis. *Cancer Res.* **76**, 1416–1428 (2016).

80. S. Zou, Q. Tong, B. Liu, W. Huang, Y. Tian, X. Fu, Targeting STAT3 in cancer immunotherapy. *Mol. Cancer* **19**, 145 (2020).

81. D. J. Jonker, L. Nott, T. Yoshino, S. Gill, J. Shapiro, A. Ohtsu, J. Zalcberg, M. M. Vickers, A. C. Wei, Y. Gao, N. C. Tebbutt, B. Markman, T. Price, T. Esaki, S. Koski, M. Hitron, W. Li, Y. Li, N. M. Magoski, C. J. Li, J. Simes, D. Tu, C. J. O'Callaghan, Napabucasin versus placebo in refractory advanced colorectal cancer: A randomised phase 3 trial. *Lancet Gastroenterol. Hepatol.* **3**, 263–270 (2018).

82. L. Chen, H. Yi, Q. Li, T. Duan, X. Liu, L. Li, H. Y. Wang, C. Xing, R. F. Wang, T-bet regulates ion channels and transporters and induces apoptosis in intestinal epithelial cells. *Adv. Sci.* **11**, e2401654, (2024).

83. T. Duan, C. Xing, J. Chu, X. Deng, Y. Du, X. Liu, Y. Hu, C. Qian, B. Yin, H. Y. Wang, R. F. Wang, ACE2-dependent and -independent SARS-CoV-2 entries dictate viral replication and inflammatory response during infection. *Nat. Cell Biol.* **26**, 628–644 (2024).

84. Q. Ma, W. Long, C. Xing, C. Jiang, J. Su, H. Y. Wang, Q. Liu, R. F. Wang, PHF20 promotes glioblastoma cell malignancies through a *WISP1/BGN*-dependent pathway. *Front. Oncol.* **10**, 573318 (2020).

85. J. E. Rosenberg, J. Hoffman-Censits, T. Powles, M. S. van der Heijden, A. V. Balar, A. Necchi, N. Dawson, P. H. O'Donnell, A. Balmanoukian, Y. Loriot, S. Srinivas, M. M. Retz, P. Grivas, R. W. Joseph, M. D. Galsky, M. T. Fleming, D. P. Petrylak, J. L. Perez-Gracia, H. A. Burris, D. Castellano, C. Canil, J. Bellmunt, D. Bajorin, D. Nickles, R. Bourgon, G. M. Frampton, N. Cui, S. Mariathasan, O. Abidoye, G. D. Fine, R. Dreicer, Atezolizumab in patients with locally advanced and metastatic urothelial carcinoma who have progressed following treatment with platinum-based chemotherapy: A single-arm, multicentre, phase 2 trial. *Lancet* **387**, 1909–1920 (2016).

86. T. Li, J. Fu, Z. Zeng, D. Cohen, J. Li, Q. Chen, B. Li, X. S. Liu, TIMER2.0 for analysis of tumor-infiltrating immune cells. *Nucleic Acids Res.* **48**, W509–W514 (2020).

87. Z. Tang, B. Kang, C. Li, T. Chen, Z. Zhang, GEPIA2: An enhanced web server for large-scale expression profiling and interactive analysis. *Nucleic Acids Res.* **47**, W556–W560 (2019).

88. H. H. Liu, M. Xie, M. D. Schneider, Z. J. Chen, Essential role of TAK1 in thymocyte development and activation. *Proc. Natl. Acad. Sci. U.S.A.* **103**, 11677–11682 (2006).

89. A. P. Martin, J. M. Alexander-Brett, C. Canasto-Chibuque, A. Garin, J. S. Bromberg, D. H. Fremont, S. A. Lira, The chemokine binding protein M3 prevents diabetes induced by multiple low doses of streptozotocin. *J. Immunol.* **178**, 4623–4631 (2007).

90. I. Godinez, M. Raffatellu, H. Chu, T. A. Paixão, T. Haneda, R. L. Santos, C. L. Bevins, R. M. Tsolis, A. J. Bäumler, Interleukin-23 orchestrates mucosal responses to *Salmonella enterica* serotype Typhimurium in the intestine. *Infect. Immun.* **77**, 387–398 (2009).

91. R. W. Jenkins, C. J. Clarke, D. Canals, A. J. Snider, C. R. Gault, L. Heffernan-Stroud, B. X. Wu, F. Simbari, P. Roddy, K. Kitatani, L. M. Obeid, Y. A. Hannun, Regulation of CC ligand 5/RANTES by acid sphingomyelinase and acid ceramidase. *J. Biol. Chem.* **286**, 13292–13303 (2011).

92. A. Li, J. King, A. Moro, M. D. Sugi, D. W. Dawson, J. Kaplan, G. Li, X. Lu, R. M. Strieter, M. Burdick, V. L. Go, H. A. Reber, G. Eibl, O. J. Hines, Overexpression of CXCL5 is associated with poor survival in patients with pancreatic cancer. *Am. J. Pathol.* **178**, 1340–1349 (2011).

93. Y. Wang, H. Yu, Y. Shan, C. Tao, F. Wu, Z. Yu, P. Guo, J. Huang, J. Li, Q. Zhu, F. Yu, Q. Song, H. Shi, M. Zhou, G. Chen, EphA1 activation promotes the homing of endothelial progenitor cells to hepatocellular carcinoma for tumor neovascularization through the SDF-1/CXCR4 signaling pathway. *J. Exp. Clin. Cancer Res.* **35**, 65 (2016).

94. Q. Xiang, L. Chen, S. Hou, J. Fang, Y. Zhou, L. Bai, Y. Liu, A. Kijlstra, P. Yang, TRAF5 and TRAF3IP2 gene polymorphisms are associated with Behcet’s disease and Vogt-Koyanagi-Harada syndrome: A case-control study. *PLOS ONE* **9**, e84214 (2014).

95. Y. Du, T. Duan, Y. Feng, Q. Liu, M. Lin, J. Cui, R. F. Wang, LRRC25 inhibits type I IFN signaling by targeting ISG15-associated RIG-I for autophagic degradation. *EMBO J.* **37**, 351–366 (2018).
